# Predictive equation derived from 6,497 doubly labelled water measurements enables the detection of erroneous self-reported energy intake

**DOI:** 10.1038/s43016-024-01089-5

**Published:** 2025-01-13

**Authors:** Rania Bajunaid, Chaoqun Niu, Catherine Hambly, Zongfang Liu, Yosuke Yamada, Heliodoro Aleman-Mateo, Liam J. Anderson, Lenore Arab, Issad Baddou, Linda Bandini, Kweku Bedu-Addo, Ellen E. Blaak, Carlijn V. C. Bouten, Soren Brage, Maciej S. Buchowski, Nancy F. Butte, Stefan G. J. A. Camps, Regina Casper, Graeme L. Close, Jamie A. Cooper, Richard Cooper, Sai Krupa Das, Peter S. W. Davies, Prasangi Dabare, Lara R. Dugas, Simon Eaton, Ulf Ekelund, Sonja Entringer, Terrence Forrester, Barry W. Fudge, Melanie Gillingham, Annelies H. Goris, Michael Gurven, Asmaa El Hamdouchi, Hinke H. Haisma, Daniel Hoffman, Marije B. Hoos, Sumei Hu, Noorjehan Joonas, Annemiek M. Joosen, Peter Katzmarzyk, Misaka Kimura, William E. Kraus, Wantanee Kriengsinyos, Rebecca Kuriyan, Robert F. Kushner, Estelle V. Lambert, Pulani Lanerolle, Christel L. Larsson, William R. Leonard, Nader Lessan, Marie Löf, Corby K. Martin, Eric Matsiko, Anine C. Medin, James C. Morehen, James P. Morton, Aviva Must, Marian L. Neuhouser, Theresa A. Nicklas, Christine D. Nyström, Robert M. Ojiambo, Kirsi H. Pietiläinen, Yannis P. Pitsiladis, Jacob Plange-Rhule, Guy Plasqui, Ross L. Prentice, Susan B. Racette, David A. Raichlen, Eric Ravussin, Leanne M. Redman, John J. Reilly, Rebecca Reynolds, Susan B. Roberts, Dulani Samaranayakem, Luis B. Sardinha, Analiza M. Silva, Anders M. Sjödin, Marina Stamatiou, Eric Stice, Samuel S. Urlacher, Ludo M. Van Etten, Edgar G. A. H. van Mil, George Wilson, Jack A. Yanovski, Tsukasa Yoshida, Xueying Zhang, Alexia J. Murphy-Alford, Srishti Sinha, Cornelia U. Loechl, Amy H. Luke, Herman Pontzer, Jennifer Rood, Hiroyuki Sagayama, Dale A. Schoeller, Klaas R. Westerterp, William W. Wong, John R. Speakman

**Affiliations:** 1 School of Biological Sciences, University of Aberdeen, Aberdeen, UK; 2 Food and Nutrition Department, King Abdulaziz University, Jeddah, Saudi Arabia; 3 Shenzhen Key Laboratory of Metabolic Health, Center for Energy Metabolism and Reproduction, Shenzhen Institute of Advanced Technology, Chinese Academy of Sciences, Shenzhen, China; 4 State Key Laboratory of Molecular Developmental Biology, Institute of Genetics and Developmental Biology, Chinese Academy of Sciences, Beijing, China; 5 Graduate School of Biomedical Engineering, Tohoku University, Sendai, Japan; 6 Department of Medicine and Science in Sports and Exercise, Graduate School of Medicine, Tohoku University, Sendai, Japan; 7 Department of Nutrition and Metabolism, Nutrition Coordination, Research Center for Food and Development (CIAD), Hermosillo, Mexico; 8 School of Sport, Exercise and Rehabilitation Sciences, University of Birmingham, Birmingham, UK; 9 David Geffen School of Medicine, University of California, Los Angeles, CA USA; 10 Unité Mixte de Recherche en Nutrition et Alimentation, CNESTEN-Université Ibn Tofail URAC39, Regional Designated Center of Nutrition Associated with AFRA/IAEA, Rabat, Morocco; 11 University of Massachusetts Chan Medical School, Worcester, MA USA; 12 Department of Physiology, Kwame Nkrumah University of Science and Technology, Kumasi, Ghana; 13 Department of Human Biology, NUTRIM, School for Metabolism and Translational Research in Metabolism, Maastricht University Medical Centre+, Maastricht, The Netherlands; 14 MRC Epidemiology Unit, University of Cambridge, Cambridge, UK; 15 Division of Gastroenterology, Hepatology and Nutritiion, Department of Medicine, Vanderbilt University, Nashville, TN USA; 16 Department of Pediatrics, Baylor College of Medicine, USDA/ARS Children’s Nutrition Research Center, Houston, TX USA; 17 Department of Psychiatry and Behavioral Sciences, Stanford University School of Medicine, Stanford, CA USA; 18 Research Institute for Sport and Exercise Sciences, Liverpool John Moores University, Liverpool, UK; 19 Nutritional Sciences, University of Wisconsin, Madison, WI USA; 20 Department of Public Health Sciences, Parkinson School of Health Sciences and Public Health, Loyola University, Maywood, IL USA; 21 USDA Human Nutrition Research Center on Aging at Tufts University, Boston, MA USA; 22 Child Health Research Centre, Centre for Children’s Health Research, University of Queensland, South Brisbane, Queensland Australia; 23 Department of Physiotherapy, Faculty of Allied Health Sciences, General Sir John Kotelawala Defence University, Kandawala, Sri Lanka; 24 Division of Epidemiology and Biostatistics, School of Public Health, University of Cape Town, Cape Town, South Africa; 25 Developmental Biology and Cancer Department, UCL Great Ormond Street Institute of Child Health, London, UK; 26 Department of Sport Medicine, Norwegian School of Sport Sciences, Oslo, Norway; 27 Charité–Universitätsmedizin Berlin, corporate member of Freie Universität Berlin and HumboldtUniversität zu Berlin, Institute of Medical Psychology, Berlin, Germany; 28 Department of Pediatrics, School of Medicine, University of California Irvine, Irvine, CA USA; 29 Solutions for Developing Countries, University of the West Indies, Kingston, Jamaica; 30 Department of Biomedical and Life Sciences, University of Glasgow, Glasgow, UK; 31 Department of Molecular and Medical Genetics, Oregon Health and Science University, Portland, OR USA; 32 Imec, OnePlanet Research Center, Wageningen, The Netherlands; 33 Department of Anthropology, University of California Santa Barbara, Santa Barbara, CA USA; 34 Population Research Centre, Faculty of Spatial Sciences, University of Groningen, Groningen, The Netherlands; 35 Department of Nutritional Sciences, Program in International Nutrition, Rutgers University, New Brunswick, NJ USA; 36 Central Health Laboratory, Ministry of Health and Wellness, Candos, Mauritius; 37 Pennington Biomedical Research Center, Baton Rouge, LA USA; 38 Institute for Active Health, Kyoto University of Advanced Science, Kyoto, Japan; 39 Department of Medicine, Duke University, Durham, NC USA; 40 Institute of Nutrition, Mahidol University, Nakhon-Pathom, Thailand; 41 Division of Nutrition, St. John’s Research Institute, Bangalore, India; 42 Feinberg School of Medicine, Northwestern University, Chicago, IL USA; 43 Health through Physical Activity, Lifestyle and Sport Research Centre (HPALS), Division of Exercise Science and Sports Medicine (ESSM), FIMS International Collaborating Centre of Sports Medicine, Department of Human Biology, Faculty of Health Sciences, University of Cape Town, Cape Town, South Africa; 44 Department of Biochemistry and Molecular Biology, Faculty of Medicine, University of Colombo, Colombo, Sri Lanka; 45 Department of Food and Nutrition and Sport Science, University of Gothenburg, Gothenburg, Sweden; 46 Department of Anthropology, Northwestern University, Evanston, IL USA; 47 Imperial College London Diabetes Centre, Abu Dhabi, United Arab Emirates; 48 Department of Metabolism, Digestion and Reproduction, Imperial College London, London, UK; 49 Department of Health, Medicine and Caring Sciences, Linköping University, Linköping, Sweden; 50 Department of Biosciences and Nutrition, Karolinska Institute, Stockholm, Sweden; 51 UR Sweden Program, University of Rwanda, Kigali, Rwanda; 52 Department of Nutrition, Institute of Basic Medical Sciences, University of Oslo, Oslo, Norway; 53 Department of Nutrition and Public Health, Faculty of Health and Sport Sciences, University of Agder, Kristiansand, Norway; 54 Tufts University School of Medicine, Boston, MA USA; 55 Division of Public Health Sciences, Fred Hutchinson Cancer Center and School of Public Health, University of Washington, Seattle, WA USA; 56 Kenya School of Medicine, Moi University, Eldoret, Kenya; 57 Rwanda Division of Basic Sciences, University of Global Health Equity, Kigali, Rwanda; 58 Obesity Research Unit, Research Program for Clinical and Molecular Metabolism, Faculty of Medicine, University of Helsinki and Abdominal Center, Obesity Center, HealthyWeightHub, Helsinki University Hospital and University of Helsinki, Helsinki, Finland; 59 School of Sport and Service Management, University of Brighton, Eastbourne, UK; 60 Department of Nutrition and Movement Sciences, Maastricht University, Maastricht, The Netherlands; 61 College of Health Solutions, Arizona State University, Phoenix, AZ USA; 62 Biological Sciences and Anthropology, University of Southern California, California, CA USA; 63 School of Psychological Sciences and Health, University of Strathclyde, Glasgow, UK; 64 Centre for Cardiovascular Sciences, Queen’s Medical Research Institute, University of Edinburgh, Edinburgh, UK; 65 Department of Medicine and Department of Epidemiology, Geisel School of Medicine at Dartmouth College, Hanover, NH USA; 66 Department of Community Medicine, Faculty of Medicine, University of Colombo, Colombo, Sri Lanka; 67 Exercise and Health Laboratory, CIPER, Faculdade Motricidade Humana, Universidade de Lisboa, Lisbon, Portugal; 68 Department of Nutrition, Exercise and Sports, Copenhagen University, Copenhagen, Denmark; 69 Department of Psychiatry and Behavioural Sciences, Stanford University, Stanford, CA USA; 70 Department of Anthropology, Baylor University, Waco, TX USA; 71 Child and Brain Development Program, CIFAR, Toronto, Ontario Canada; 72 Maastricht University, Brightlands Campus Greenport Venlo and Lifestyle Medicine Center for Children, Jeroen Bosch Hospital, ’s-Hertogenbosch, The Netherlands; 73 Section on Growth and Obesity, Division of Intramural Research, Eunice Kennedy Shriver National Institute of Child Health and Human Development, National Institutes of Health Bethesda, Bethesda, MD USA; 74 National Institute of Health and Nutrition, National Institutes of Biomedical Innovation, Health and Nutrition, Osaka, Japan; 75 Department of Nutritional Sciences, International Atomc Energy Agency, Vienna, Austria; 76 Duke Global Health Institute, Duke University, Durham, NC USA; 77 Department of Evolutionary Anthropology, Duke University, Durham, NC USA; 78 Faculty of Health and Sport Sciences, University of Tsukuba, Ibaraki, Japan; 79 Biotechnology Center and Department of Nutritional Sciences, University of Wisconsin, Madison, WI USA; 80 Institute of Health Sciences, China Medical University, Shenyang, China

**Keywords:** Obesity, Epidemiology

## Abstract

Nutritional epidemiology aims to link dietary exposures to chronic disease, but the instruments for evaluating dietary intake are inaccurate. One way to identify unreliable data and the sources of errors is to compare estimated intakes with the total energy expenditure (TEE). In this study, we used the International Atomic Energy Agency Doubly Labeled Water Database to derive a predictive equation for TEE using 6,497 measures of TEE in individuals aged 4 to 96 years. The resultant regression equation predicts expected TEE from easily acquired variables, such as body weight, age and sex, with 95% predictive limits that can be used to screen for misreporting by participants in dietary studies. We applied the equation to two large datasets (National Diet and Nutrition Survey and National Health and Nutrition Examination Survey) and found that the level of misreporting was 27.4%. The macronutrient composition from dietary reports in these studies was systematically biased as the level of misreporting increased, leading to potentially spurious associations between diet components and body mass index.

## Abstract (web summary)

This study presents a predictive equation for total energy expenditure derived from doubly labelled water measurements. Applying this equation to two large datasets (the National Diet and Nutrition Survey and National Health and Nutrition Examination Survey) shows that the misreporting of total energy intake is greater than 50%, with important implications for macronutrient availability.

## Main

Diet is a major modifiable factor implicated in many chronic diseases. A persistent problem, however, is accurate quantification of what people eat. Without this information, it is impossible to link nutritional exposures to disease outcomes^1^. The commonest tool for assessing diet is the food frequency questionnaire, which asks individuals to recall frequencies of intake of various foods over protracted periods. Shorter-term instruments used to identify detailed dietary intake require individuals to estimate and record the amount of food that they are eating (for example, food intake diaries) or recall what they ate in the recent past (for example, 24 h recall)^2^. All these methods are prone to ‘misreporting’ because people cannot accurately estimate the amount of food they are eating, have fallible memories for their intake^3,4^ and may, in some cases, deliberately falsify reports^4–7^. In addition, for food intake diaries, people may react during the period of recording by changing their intake^7^. ‘Misreporting’ also includes a range of other issues, such as how dietary intake reported by participants is converted into energy and nutrients by the investigator, for example, by assuming that all apples are the same size. Moreover, because food intake varies enormously on a day-to-day basis^8,9^, individuals may faithfully report what they eat on a given day, but that day may be unrepresentative of what they routinely eat (often called ‘under- or overeating’)^10^. Making repeated measures using the same instrument on different days may minimize this last problem, but given the variability in daily intake, the number of days that would be required to reduce the variation to a reasonable level is unrealistic for most population survey studies^9,11^. The problems of misreporting and under- or overeating likely occur simultaneously in many situations. Henceforth, for brevity, we will refer to the phenomena of misreporting and under- or overeating as misreporting.

Misreporting has real negative consequences. For example, the failure to recognize these problems led to decades of thinking that people with obesity had very low energy intakes, and hence the positive energy balance leading to their obesity must be a defect in energy expenditure. It later turned out that measured energy expenditures among people with obesity are not low^12^. The problem of misreporting is so ubiquitous and severe that there have been calls for journals to stop publishing studies based on methods that depend on participants estimating their own dietary intake^13^. Yet, such studies continue to proliferate in the literature. This popularity is perhaps because these tools continue to be endorsed by various government bodies, such as the National Cancer Institute (https://epi.grants.cancer.gov/asa24/respondent/validation.html), people have been convinced by arguments that they do have utility^14^ and because there are no other feasible, affordable and practical ways to assess total dietary intake.

When the problem of misreporting was first recognized in the late 1980s^15–17^, an attempt was made to define cut-off limits by which intake records could be screened for credibility^18^. This was initially done by predicting a person’s basal energy expenditure (BEE) using prediction equations based on height, body weight, sex and age. The estimated BEE was then multiplied by 1.35 on the presumption that a daily total energy expenditure (TEE) lower than 1.35 × BEE would be incompatible with survival. This limit is generally referred to as the ‘Goldberg cut-off’. However, this approach is susceptible to two major problems: error in the predicted resting metabolic rate and the arbitrary nature of the 1.35 multiplier. Accordingly, the method can only detect and exclude very low reported intakes^19^ and many other inaccurate estimates may evade detection. These problems were detailed by Black^9^, who modified the cut-off taking into account levels of physical activity and measurement errors in the BEE. This led to the ‘modified Goldberg cut-off’. Nevertheless, despite these improvements, the method still relies on the estimated BEE and requires some unverified assumption of the expected physical activity level (PAL).

The doubly labelled water (DLW) technique measures energy expenditure directly from the elimination of isotopes of oxygen and hydrogen introduced into the body in water^20^. The method has an analytical error of about 7% depending on the equation that is used^21^. McCrory et al.^22^ introduced a new way to use measurements based on DLW to screen dietary recalls. This method was based on predicting TEE from regression equations based on earlier DLW measurements using age, sex, weight and height as predictors. The TEE estimate used by McCrory et al.^22^ employed the equation of Vinken et al.^23^. The standard deviation (SD) of the prediction was then used to define cut-offs (at 1 and 2 SDs) to identify under- and over-reporters of food intake. Although the approach of McCrory et al.^22^ has many benefits compared with the use of the Goldberg and modified Goldberg cut-offs, it is hampered by its reliance on equations derived from a relatively small sample of 93 individuals (44 males and 49 females), which included no men between the ages of 28 and 60, and no children or adolescents. Moreover, the cut-off limits of 1 and 2 SDs are also arbitrary.

In this context, we have assembled a database of DLW measurements of healthy individuals^24^. The database includes measurements of over 7,500 individuals of diverse ethnicity aged 8 days to 96 years. Hence, energy demands through the life course have been documented in unrivalled detail^25^ and other factors, such as ambient temperature, that may influence TEE have been elucidated^26^. Although we previously published prediction equations for TEE using this database^25^, these equations took as their inputs fat-free mass (FFM) and fat mass (FM), measures that are routinely unavailable in dietary surveys. In this study, we derived prediction equations for TEE and confidence limits based on easily measured input parameters using 6,497 available data divided into 5,899 individuals as the analysis set and 598 as the validation set. These equations allow the identification of individuals who may be under- or over-reporting intake in dietary surveys. We demonstrate their use in two publicly available dietary surveys, namely, the National Diet and Nutrition Survey (NDNS)^27^ and the National Health and Nutrition Examination Survey (NHANES)^28^, which include a total of 18,567 individuals, showing that the level of dietary under-reporting is underestimated by previous tools and that this introduces bias in evaluating dietary composition.

## Results

### Predictive models

We used two main approaches. The first was classical general linear regression modelling including putatively important factors and their two-way interactions as predictor variables. The variables included were body weight, height, age, age × age (age^2^), self-reported ethnicity, sex and elevation above sea level of the measurement site. In the second approach, we used three machine learning models (Random Forest, XGBoost and Support Vector Regression) to derive predictions. These machine learning models did not improve on the classical general linear regression modelling (Supplementary Information and Supplementary Table 1), probably because the predictors in question were linearly related to the output variable. Hence, further treatment was based on only the general linear regression modelling.

The derived significant predictors and their regression coefficients are reported in Table 1. The most significant predictor was the natural logarithm of body weight (ln(BW)). The other primary variables, that is, height, age, age^2^, elevation and sex, were all highly significant (*P* < 10^−6^ in all cases). Females had lower TEE than males. White (non-Hispanic) participants tended to have slightly higher TEE and African participants living outside Africa (AA) slightly lower, and both were highly significant effects (*P* < 10^−9^). The effects of other groups, however, did not reach significance. The final model explained 69.8% of the variation in ln(TEE).

**Table 1:** Significant terms in the general linear model analysis (10 decimal places) predicting TEE

Term	Coefficient	SE coefficient	*T* value	*P* value
Constant	−0.21723930921	0.0757	−2.87	0.0041
ln[BW (kg)]	0.41666419569	0.00958	43.51	<10^−9^
Height (cm)	0.00656496388	0.000618	10.62	<10^−9^
Age (yr)	−0.02054339322	0.00218	−9.41	<10^−9^
Age^2^ (yr^2^)	0.00033079019	0.000037	9.06	<10^−9^
ln[Elevation (m)]	0.09126350903	0.0186	4.89	0.000001
Sex	−0.04091711710	0.00769	−5.32	0.00000011
Ethnicity^a^				
A	0.01939639976	0.00749	2.59	0.0096
AA	−0.03899332615	0.00544	−7.17	<10^−9^
AS	0.00623768257	0.00808	0.77	0.44
W	0.02625775059	0.00397	6.62	<10^−9^
H	−0.01554772302	0.00982	−1.58	0.11
NA	0.00358921276	0.00636	0.56	0.57
Height × ln[Elevation (m)] (cm)	−0.00067594646	0.000136	−4.98	0.00000066
Age × Age^2^ (yr^3^)	−0.00000185178	0.000000	−8.93	<10^−9^
Age × ln[Elevation (m)] (yr)	0.00201815477	0.000383	5.28	0.00000014
Age^2^ × ln[Elevation (m)] (yr^2^)	−0.00002262281	0.000004	−5.89	0.0000000041
ln[Elevation (m)] × Sex	−0.00694699228	0.00179	−3.87	0.00011

^a^A, African; AA, African living outside Africa; AS, Asian; W, White; H, Hispanic; NA, not available. SE, standard deviation.

In this analysis, we were able to derive a predictive equation with each coefficient reduced to four significant figures. The difference between the calculations conducted using this equation and an equation using full precision (10 decimal places) for the coefficients in Table 1 for a random sample of 250 measurements was 0.03%. Reducing the significant figures to three increased the discrepancy by a factor of ten (0.4%).

$$\begin{array}{l}\mathrm{ln}\left({{\rm{TEE}}}\right)=\\\qquad\qquad\quad-0.2172+0.4167\times \mathrm{ln}\left({{\rm{BW}}}\right)+0.006565\times {{\rm{Height}}}\\\qquad\qquad\quad-0.02054\times {{\rm{Age}}}+0.0003308{{{\rm{Age}}}}^{2}-0.000001852\\\qquad\qquad\quad\times{{{\rm{Age}}}}^{3}+0.09126\times \mathrm{ln}\left({{\rm{Elevation}}}\right)-0.04092\times {{\rm{Sex}}}\\\qquad\qquad\quad+0.01940\times {\rm{A}}-0.03899\times {{\rm{AA}}}+0.006238\times {{\rm{AS}}}\\\qquad\qquad\quad+0.02626\times {\rm{W}}-0.0155\times {\rm{H}}+0.003589\times {{\rm{NA}}}\\\qquad\qquad\quad-0.0006759\times {{\rm{Height}}}\times \mathrm{ln}\left({{\rm{Elevation}}}\right)+0.002018\\\qquad\qquad\quad\times {{\rm{Age}}}\times \mathrm{ln}\left({{\rm{Elevation}}}\right)-0.00002262\times {{{\rm{Age}}}}^{2}\\\qquad\qquad\quad\times \mathrm{ln}\left({{\rm{Elevation}}}\right)-0.006947\times {{\rm{Sex}}}\times\mathrm{ln}({{\rm{Elevation}}})\end{array}$$

Here, TEE is in megajoules per day, BW is in kilograms, height is in centimetres, age is in years, sex is coded −1 for males and +1 for females, and the elevation of the measurement location is in metres. For the self-reported ethnicity codes, for African, A was 1 and 0 otherwise, for African individuals living outside Africa, AA was 1 and 0 otherwise, for Asian, AS was 1 and 0 otherwise, for white, W was 1 and 0 otherwise, for Hispanic, H was 1 and 0 otherwise, for not available, NA was 1 and 0 otherwise. Mixed race individuals were coded as NA (see Methods). Two worked examples for the calculation of TEE for two different individuals are provided in Supplementary Table 7.

The residuals of the prediction were well distributed with respect to the major predictors, suggesting that the prediction was not biased (Supplementary Fig. 1). In addition, there was no significant relationship between the residual of the prediction and weight change during the measurement period (*n* = 3,088 with reported weight change, *F* = 0.19 and *P* = 0.665; Supplementary Fig. 2a), suggesting that the energy expenditure from this predictive model is a good proxy for intake in individuals that are not attempting to lose or gain weight or suffering loss of appetite due to illness. That is because the metabolic rate generally declines when individuals are engaged in deliberate weight loss, and the opposite happens during overfeeding. Hence, if individuals were not in energy balance, we would expect a positive relationship between weight change and residual energy expenditure. Ninety-five per cent predictive intervals (95% PI) are the range of values that are 95% likely to contain the true value for a single new observation based on specific values of the predictor variables. The predictive interval depends on the *T*-critical value for the given confidence, the estimated mean and the standard error of the response variable, the sum of squares and the specific and mean values of the predictor variables, and the total sample size on which the prediction equation is based. For all of the test samples, we used standard statistical software (Minitab v19, https://www.minitab.com) to calculate the upper and lower predictive intervals and then defined two additional equations to identify the 95% PI around the predictions.

This gave

$${{\rm{Lower}}}\;95 \%\; {{\rm{PI}}}=\left(\;{p{\rm{TEE}}}\times 0.7466\right)-1.5405$$

$${{\rm{Upper}}}\;95 \%\; {{\rm{PI}}}=\left(\;{p{\rm{TEE}}}\times 1.3395\right)+2.7668$$

where *p*TEE is the predicted mean TEE (MJ d^−1^). This interval provides an objective evaluation of the confidence that can be placed in any given prediction using the derived regression equation. Using this predictive interval to screen observations is a superior approach to previous attempts to screen dietary reports, which were all based on arbitrary cut-off points.

For the 598 individuals in the validation set, we derived the predicted TEE and the upper and lower 95% PI for the mean estimates using the equations derived above (Supplementary Fig. 2). We then counted the number of actual measurements of TEE in the validation set that fell outside the predictive interval for TEE (Supplementary Fig. 2b). In total, from 598 measurements in the validation set, 14 fell below the lower predictive interval (2.3%) and 20 were above the upper predicted limit (3.3%). The validation dataset confirmed that 94.6% of independent TEE measurements were within these 95% predictive limits (Supplementary Fig. 2b). We then explored whether the equations could produce credible predictions for groups that were not included in the original derivation but whose data were available in the database, specifically 246 athletes and individuals engaged in unusual levels of physical activity and 176 females during reproduction. The predictions significantly underestimated the observed expenditures of all these groups (Supplementary Information, Supplementary Fig. 3 and Supplementary Table 6). Therefore, the prediction equation derived here cannot be used for these populations.

### Application to exemplary survey data

Demographic statistics for the individuals used in the comparison are presented in Supplementary Table 2. In total, there were 12,694 records available in NDNS and 5,873 in NHANES. On average, the individuals in NHANES were around 6–10 years older than those in NDNS. Twenty-five per cent of the sample in NHANES self-reported as African living outside Africa and 22% as Hispanic. In contrast, 94% of the participants in NDNS self-reported as white. We compared the net energy intake with the predicted TEE from the above equation. Using the predictive equations developed above, the number and percentage of individuals that fell outside the predicted limits (both over and under) and within the predicted limits are shown in Table 2, stratified by data source, age (adults versus children) and sex.

**Table 2:** Summary of observations inside and outside the tolerance limits in the NDNS and NHANES datasets

	Number underestimated	Percentage of total	Number within range	Percentage of total	Number overestimated	Percentage of total	Total
**NDNS**							
Male children	436	17.40	2,067	82.48	3	0.12	2,506
Female children	371	15.64	2,000	84.32	1	0.04	2,372
Male adults	1,250	37.96	2,039	61.92	4	0.12	3,293
Female adults	1,341	29.65	3,180	70.31	2	0.04	4,523
Male all	1,686	29.07	4,106	70.81	7	0.12	5,799
Female all	1,712	24.83	5,180	75.13	3	0.04	6,895
**NHANES**							
Male children	135	19.01	562	79.15	13	1.83	710
Female children	108	14.32	634	84.08	12	1.59	754
Male adults	691	32.76	1,372	65.05	46	2.18	2,109
Female adults	654	28.43	1,625	70.65	21	0.9	2,300
Male all	826	29.30	1,934	68.61	59	2.09	2,819
Female all	762	24.95	2,259	73.97	33	1.08	3,054

The data show the numbers and percentages of participants that fall inside and outside the tolerance limits in the NDNS dataset (years 1–11) and the NHANES dataset (2017–2018).

For adults in NHANES, approximately 67.9% of dietary reports were within the predictive interval (65.1% for males and 70.7% for females). For children, the percentage within range was considerably higher (by 13–14%) than for adults. A similar pattern was noted for the NDNS data. For adults, 61.9% of males and 70.3% of females were within the prediction interval. The percentage of children in range was about 20% higher than for adults. This means that a large percentage of data fell below the lower predictive interval due to either undereating or misreporting. For NHANES, the figures for adults were 32.8% in males and 28.4% in females. For NDNS, the overall values for adults were 38% in males and 29.7% in females. In both surveys, children were less likely to under-report/undereat by 14–21%. We compared the detection of under-reporting using our equation with the previous models proposed by Goldberg et al.^18^, Black^9^ and McCrory et al.^22^ (Supplementary Table 3). On average, the Goldberg cut-off indicated 16.7% and the Black cut-off 23.4% under-reporting, both far less than is indicated here. The level of under-reporting identified using the McCrory et al.^22^ equation depended very heavily on whether 1 or 2 SDs were used as the cut-off. With 1 SD, the level of under-reporting was greater than we predicted (47–63%), but it was much less using 2 SDs (10.7–21.4%).

### Effects of age and body mass index on under-reporting

**Fig. 1:**
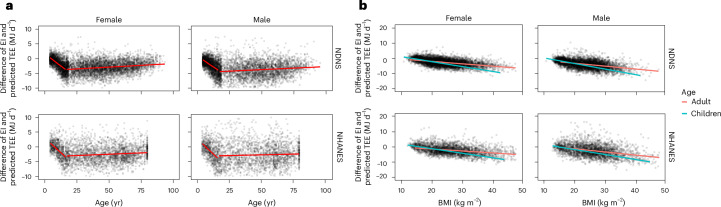
Misreporting in relation to age, BMI and sex. **a**, Comparison of the difference between predicted TEE and self-reported energy intake (EI) in the NDNS (*n* = 12,694) and NHANES (*n* = 5,873) datasets in relation to age for children (≤16 yr) and adults (>16 yr). **b**, Comparison of the difference between predicted TEE and self-reported energy intake in the same datasets in relation to BMI for children (≤16 yr) and adults (>16 yr). Negative values show observations lower than prediction and positive values show prediction higher than observation.

We plotted the difference between the survey estimate of daily energy intake and the predicted TEE as a function of age and body mass index (BMI) for both the NDNS and NHANES datasets (Fig. 1). In adults, the extent of under-reporting was almost independent of age in both datasets, although there was a slight improvement with age in the NDNS dataset (*P* < 0.001). The average discrepancy in the NDNS was 3.5 MJ for both females and males. In NHANES, the average discrepancy for males was 1.8 MJ and for females it was 2.8 MJ. In both the NHANES and NDNS surveys, the data for the very young, whose surveys were generally completed by their care providers, were at or slightly above the expected intakes. There was a strong deterioration in the number of plausible estimates through childhood as the children started to complete their own surveys, until, by age 16, the discrepancies matched the adult levels (Fig. 1a). The deficit between reported intake and predicted expenditure was strongly negatively correlated with individual BMI (Fig. 1b). In both surveys, there was no discrepancy between what adults and children with a BMI of around 15–20 kg m^−2^ reported eating and their predicted expenditure. However, the discrepancy got larger as the BMI increased in both adults and children. The effect in children was greater than in adults. Hence, in NDNS, a child with a BMI of 40 kg m^−2^ had a discrepancy of 9 MJ d^−1^, while an adult with a BMI of 40 kg m^−2^ had a discrepancy on average of only 5 MJ d^−1^. In NHANES, for a BMI of 40 kg m^−2^, the discrepancies were 8 MJ d^−1^ for children and 4 MJ d^−1^ for adults.

### Under-reporting in relation to macronutrient intake

**Fig. 2:**
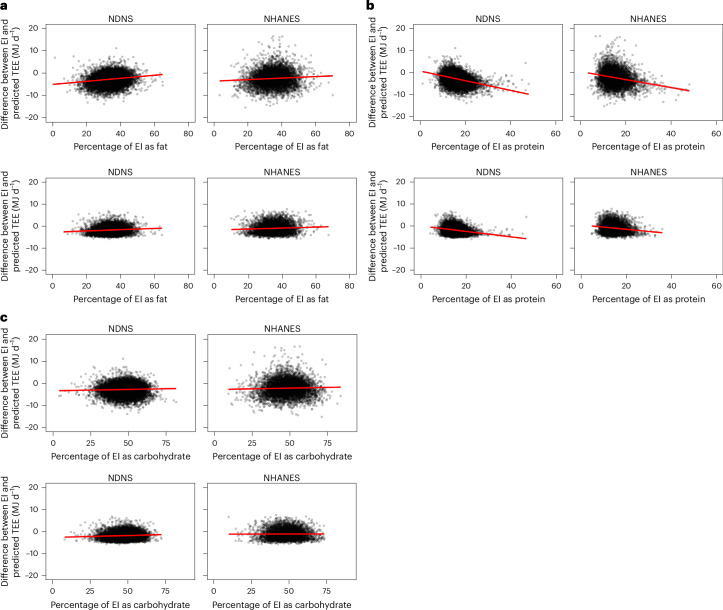
Misreporting and macronutrient intake. **a**–**c**, The discrepancy between the predicted TEE and the reported energy intake in the NHANES and NDNS surveys plotted against the self-reported intakes of fat (**a**), protein (**b**) and carbohydrates (**c**) as a percentage of the total energy. For each macronutrient, the top two plots show data from the whole sample (full data) and the bottom two plots show the data from the sample screened to include only those individuals within the predictive interval of the equation (screened). Significant effects in the whole sample were severely attenuated in the screened sample (see Table 3 for regression details).

Next, we explored the relationship between the discrepancy in energy intake and the proportional macronutrient composition (percentage energy) of the reported diet (Table 3). If there was no bias in the under-reporting, then we would expect no differences in the coefficients with respect to the different macronutrients. Contrasting this prediction in the data that were not screened, there was a strong relationship between the reported percentage of energy as protein in the diet and the absolute size of the energy discrepancy (Fig. 2). As the level of protein in the diet increased, the discrepancy became more negative. For each 1.0% increase in reported protein energy, the difference between reported energy intake and actual intake decreased by around 200 kJ d^−1^ in both NDNS and NHANES (Table 3). Note that as most data fall below the line of equality, this negative relationship means that as the self-reported percentage of protein in the diet increased, the discrepancy between the self-reported total energy intake and the predicted total energy expenditure got larger (Fig. 2). In contrast, as the percentage of fat energy in the diet increased, the discrepancy between the reported and predicted intake became more positive and the discrepancy got smaller (Fig. 2). The effect was smaller than the impact of protein and was different between surveys. The effect in NDNS was approximately twice as large as that in NHANES. In NDNS, there was no significant effect of the percentage of carbohydrate energy in the diet on the discrepancy, but in NHANES, carbohydrates had a similar direction of effect as protein, but the effect size was about a tenth as large (Fig. 2 and Table 3). These differences indicate that the assumption in dietary surveys that diet composition is independent of the extent of misreporting is likely to be false. Individuals who under-reported their total energy intake also reported a greater percentage of protein energy and a reduced percentage of fat in their diets (Fig. 2). These effects are unlikely to be limited to macronutrients as the total energy and macronutrient composition are derived from the self-reported list of food items consumed. The bias in macronutrient reporting found in this study strongly suggests corresponding recall bias in the types of food recalled and thus micronutrient intakes as well. The magnitude of this effect may well depend on the food and nutrient examined.

**Table 3:** Relationships between the discrepancy of intake to expenditure and self-reported dietary macronutrient composition

NDNS (full data)			
Term	Coefficient	SE coefficient	*P* value
Constant	−1,360.8	366.3	0.0002
Percentage carbohydrate	0.64	3.86	0.87
Percentage protein	−207.3	6.42	<0.0001
Percentage fat	53.40	4.49	<0.0001
*R*^2^ (%)			12.24
NDNS (screened)			
Term	Coefficient	SE coefficient	*P* value
Constant	−2,184.56	302.94	<0.0001
Percentage carbohydrate	17.25	3.16	< 0.0001
Percentage protein	−105.67	5.96	<0.0001
Percentage fat	35.97	3.79	<0.0001
*R*^2^ (%)			6.23
NHANES (full data)			
Term	Coefficient	SE coefficient	*P* value
Constant	1,025.15	936.0	0.27
Percentage carbohydrate	−20.94	9.61	0.03
Percentage protein	−207.65	13.77	<0.0001
Percentage fat	25.27	10.47	0.02
*R*^2^ (%)			5.85
NHANES (screened)			
Term	Coefficient	SE coefficient	*P* value
Constant	633.72	734.47	0.39
Percentage carbohydrate	−11.62	7.53	0.12
Percentage protein	−112.42	11.74	<0.0001
Percentage fat	16.42	8.29	0.048
*R*^2^ (%)			3.16

Multiple regression analysis of the discrepancy between intake and predicted expenditure and the self-reported macronutrient composition of the diet in the NDNS and NHANES surveys. In both cases, ‘full data’ refers to the analysis of the whole dataset and ‘screened’ relates to the analysis of the screened data.

Screening the data using the tool presented here to remove those outside the predictive interval (that is, under- and over-reporters) massively attenuated these bias effects (Fig. 2 and Table 3). However, this course of action necessitates the removal of a large percentage of the collected dietary records and this is wasteful of the effort to collect such data. There are several possible alternative approaches. One potential method is to conduct the analysis including or excluding the data outside the predictive interval. If there are no biases, then the outcomes should be the same and in such a case reverting to the full dataset would be appropriate. Another alternative is to model the factors that influence the differences between the data identified as implausible and attempt some form of correction of the problematical dietary records. Whatever the adopted approach, we suggest that by using the tool that we provide here, nutritional epidemiologists may enhance the quality of their work and have greater confidence in their conclusions.

**Fig. 3:**
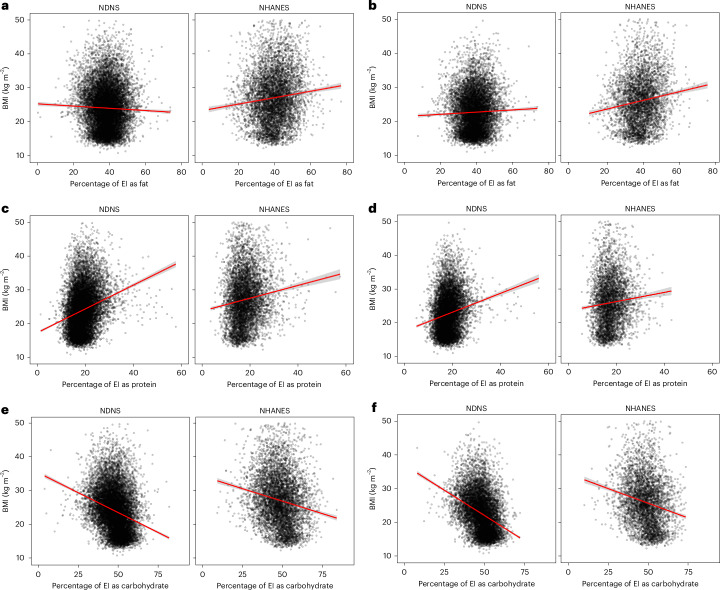
Relationships between the reported dietary intakes of macronutrients and BMI. **a**–**f**, Relationships between BMI and the intakes of fat (**a**,**b**), protein (**c**,**d**) and carbohydrate (**e**,**f**) for the NHANES and NDNS surveys. Panels **a**, **c** and **e** show the data for the whole sample and panels **b**, **d** and **f** show the data for those individuals whose total energy intake was within the predictive interval (that is, excluding under- and over-reporters).

As there is a systematic trend between macronutrient intake and the extent of under-reporting and because under-reporting is related to BMI, there was a strong positive relationship between the reported dietary intakes of protein and BMI in both surveys (Fig. 3 and Table 4). In contrast, there was a strong negative effect for carbohydrate intake (Fig. 3 and Table 4), while the relationship of fat intake to BMI differed between the surveys, being positive in NHANES and negative in NDNS. The strengths and gradients of these effects were significantly impacted by restricting the analysis to only those data within the acceptable range. The gradient and *R*^2^ values of the relationship between BMI and protein were both strongly reduced (Fig. 3 and Table 4), while the negative gradient for the relationship between BMI and carbohydrates became more negative and the *R*^2^ value approximately doubled. Higher carbohydrate intake was therefore strongly associated with a lower BMI. The relationship for fat content also became stronger (*R*^2^ increased) and the gradients, previously showing different trends for the two surveys, were both positive. Higher reported fat and protein intakes were both strongly associated with a higher BMI. All the relationships were highly significant (*P* < 10^−4^, Table 4).

**Table 4:** Relationships between macronutrient intake and BMI in both datasets

Macronutrient	Survey	Whole data			Within 95% PI		
		Gradient	*R*^2^	*P*	Gradient	*R*^2^	*P*
Percentage fat	NHANES	+0.118	0.0109	<10^−15^	+0.156	0.019	<10^−15^
	NDNS	−0.0376	0.0011	0.000099	+0.0355	0.0011	0.0014
Percentage carbohydrate	NHANES	−0.1498	0.0276	<10^−15^	−0.1763	0.038	<10^−15^
	NDNS	−0.2306	0.0709	<10^−15^	−0.2966	0.123	<10^−15^
Percentage protein	NHANES	+0.2309	0.0142	<10^−15^	+0.1591	0.006	<10^−6^
	NDNS	+0.4227	0.0578	<10^−15^	+0.3328	0.030	<10^−15^

Multiple regression analysis of macronutrient intake and BMI in the NHANES and NDNS datasets using the whole data and only the observations where the total dietary intake was within the predictive interval of the regression model. Gradient represents the change in BMI for each 1% change in macronutrient intake.

## Discussion

### Impact of repeated recalls on survey validity

If the problem with misreported intakes reflects undereating rather than under-reporting, then making repeated surveys should alleviate the issue, unless the undereating is a direct response to the survey instrument. This could be an issue for food diaries, but should not be an issue with 24 h recall. However, if participants developed reporting fatigue, one might anticipate that the accuracy of reporting would decline as the number of surveys was increased. In NDNS, some participants completed four surveys, while in NHANES, some participants completed two. The number of individuals who fell within the expected range was independent of the day of survey in NDNS (Supplementary Table 4a). This suggested that there was no survey fatigue across the 4 days. When the average intakes were taken across multiple days, this did not improve the percentage that fell within the predicted range (Supplementary Table 4b). This indicates that the general problem of misreporting is not undereating but under-reporting, and that such under-reporting was consistent across days. The consistent magnitude of misreporting suggests that there is little benefit of completing multiple surveys as a mechanism to eliminate misreporting. Similar patterns were found for the NHANES analysis, where the percentage of individuals in the expected range was not different between the two surveys and accuracy was not improved by taking the average (Supplementary Table 5).

The predictive equation based on general linear modelling explained >69% of the variation in TEE. This is less than was achieved with an equation based on fat-free mass, fat mass and age derived from the same dataset^25^, which explained 83% of the variation (but in a sample restricted only to adults). The significant effects of additional variables beyond body weight, such as height, sex and self-reported ethnicity, therefore likely exert their effects because these traits also impact FFM as a component of body weight. For example, females of a given height and weight tend to have greater fat mass and lower FFM than males. Thus, when body weight rather than FFM is used as a predictor, sex also enters as a significant term; conversely, when FFM is used as a predictor, sex is no longer significant^24^. The effects of elevation were unanticipated and their numerous interactions with other variables suggest that this may also be related to trends in FFM with elevation. On average, it gets colder as the elevation increases. However, it is unlikely that the elevation effect is due to declining ambient temperature, as in a subset of the same data (restricted to the USA), we found no effect of ambient temperature on TEE^26^. Independent of body composition, it is established that elevation affects BEE, which is a major component of TEE^29^. The age effect included squared and cubed terms, also consistent with previous work suggesting nonlinear impacts of age on metabolic rate^25,30^.

If FFM and FM explain more of the variation in TEE, then a valid question is why not use that equation on which to base the screening? The problem with such an approach, however, is the accuracy of the estimates of FFM and FM. In the equations derived previously, the percentage FFM and FM came from isotope dilution estimates of body water, which derive from the DLW method. Performing isotope dilution on all survey participants in large surveys would be challenging and costly. Alternative approaches to measuring FFM in survey settings, however, are less accurate. Thus, the extra predictability of TEE afforded by having estimates of FFM and FM is negated by the reduced accuracy of cheap FFM and FM assessments. A second question is how do the equations take into account different levels of physical activity? The modified Goldberg approach accounts for this by using different levels of PAL (the ratio of TEE to BEE). The main problem with this is equating the PAL level to a level of physical activity^31^ and the inaccuracies involved in people self-reporting how active they are. In the current approach, we included a large sample of individuals who have a diversity of PAL levels that make up the total TEE. By predicting TEE directly, we automatically account for the diverse effects that other factors may have on PAL and hence TEE, such as age, sex and ethnicity. The 95% prediction limits therefore cover the vast majority of individuals. The exceptions are groups who have particularly active lifestyles. We showed that the equations significantly underestimate the expenditure of such groups (Supplementary Fig. 3). In addition, the equations significantly underestimated TEE in pregnant and lactating females.

### Detection of under- or over-reporting and under- or overeating

There was very little change in the level of undereating/under-reporting with age. In contrast, age has previously been identified as a strong factor for under-reporting energy intake^32–34^. In a previous study, 36% of women and 34% men aged 40–69 years underestimated energy intake^35^. Similarly, in a different study, among women and men (*n* = 28) aged between 35 and 67 years, the discrepancy was 19% (ref. ^36^). There was no increase in the level of under-reporting in individuals who were aged >70 years, where one might anticipate that memory functions might impair recall fidelity. In contrast, for young children, where intake diaries are generally completed by an adult, the agreement between expectation from the equation and the estimates from the survey report was much better.

It is often claimed that instruments in dietary survey work were designed to assess the types of food being consumed and not the total energy intake. Hence, reported total energy intake could be incorrect, but that does not necessarily mean that the percentage macronutrient compositions are erroneous because the error may be unbiased. If so, it would mean that dietary survey work might not be as flawed as is often claimed^13^. If misreporting was unbiased, then the discrepancy between the survey intake and the DLW prediction would be unrelated to the macronutrient composition of the reported diet. In other words, each macronutrient would have the same relationship to the level of misreporting. This was not the case. The level of reporting was strongly related to the reported protein intake, with lesser and opposite effects for fat. Carbohydrate had lower and contrasting effects across surveys. When people under-reported their intake, they tended to also report an elevated percentage of protein intake and a lower percentage of fat intake. The relationship between misreporting and macronutrient composition is consistent with previous work showing that under-reporters of total intake also report consuming a greater percentage of protein^37–43^. In these previous studies, only Cook et al.^37^, Bel-Serrat et al.^42^ and Previdelli et al.^43^ observed a contrasting effect for fat consistent with the magnitude of the different fat effects between surveys observed here^44^. Because under-reporting is also strongly linked to BMI (Fig. 1), there is enormous potential to misinterpret associations between dietary survey reports of macronutrient intake and BMI (Fig. 2). BMI-related biases in reporting could affect other analyses as well, for example, the relationship between particular food types and markers of inflammation. We show here that using our tool to identify misreporting individuals, the associations between dietary self-reported macronutrient intake and BMI were significantly modified, indicating the utility of the tool.

### Limitations

We used estimates of TEE derived from the DLW method to infer food energy intake. There are several assumptions in this procedure. Converting CO_2_ production into energy expenditure depends on knowledge of the respiratory quotient (RQ). In general, the RQ was not known in the studies submitted to the database and an assumed value of 0.83 was used. Deviations from that value due, for example, to having a diet particularly rich in fat or carbohydrates adds error into the estimated TEE. That might then complicate comparing the extent of misreporting with dietary composition. For example, if an individual had an RQ of 0.78, reflecting high fat intake, and we assumed an RQ of 0.83, then we would overestimate TEE and make under-reporting more likely to be detected. However, the difference in TEE in this instance would only be 4.5%, and hence this would have only a marginal impact on the detection of misreporting in relation to fat intake. In addition, converting energy expenditure into an estimate of food intake assumes that the individuals are in energy balance over the time course of the measurement. We consider that the individuals in the sample used to derive the equation were likely to be in energy balance because the residual TEE values were not related to weight change over the interval of the measurement. This is not necessarily the case for individuals involved in dietary surveys and one should always be cautious that deviations from the predictions are not due to misreporting, but because the person was under- or overeating. Although we had a large sample of TEE data, the predictive model explained only 69% of the variation in TEE and the resultant absolute error in the predicted values of the test set averaged 11.2%. Because we used the 95% PI around the average to define implausible records, then by definition 5% of such records will be erroneously identified and in fact be valid reports. In the future, this prediction may be improved by integrating independent measures of physical activity, for example, by accelerometry, into the model. However, the utility of this extra information in terms of detecting erroneous food intake reports in dietary survey work may be limited because few such surveys have objective measures of physical activity collected by, for example, accelerometry.

### Implications and future directions

Accurately measuring what people eat is essential for understanding the consequences of components of food intake for health. It also contributes to our understanding of many other areas, including food security and quantifying food waste. The main tools that we currently use to do this were developed more than 50 years ago, they depend on self-report and are widely acknowledged to provide inaccurate information. Tools to identify misreported data already exist. In this study, we developed an enhanced approach to identify potentially erroneous and implausible reports. The tool is not perfect and it will itself misidentify about 5% of reports as wrong when they are in fact correct, but it improves on previous approaches to identify problematical data. Applying the tool to two large surveys suggested that 27.4% of the dietary reports had implausible energy intakes and probably therefore erroneous intake of macro- and micronutrients. Ultimately, the main benefit of this tool is that it may highlight the true level of dietary misreporting when using existing methods and drive us towards innovating radical approaches that do not rely so much (or at all) on self-report.

## Methods

This is a retrospective analysis of cross-sectional data. Data collection started before establishment of the clinical trials registry. The goals of this analysis were pre-registered on the International Atomic Energy Agency (IAEA) DLW Database site in 2020. The original data on which it is based were subject to ethical review at diverse institutions.

### Developing the prediction algorithm

The predictive algorithm was derived from an analysis of measurements submitted to the IAEA DLW Database (version 3.6; dlwdatabase.org). This included data derived from DLW studies in 32 countries with 7,646 male and female participants, compiled from 128 different published and unpublished studies. The measurements relate to individuals who were not engaged in dietary or exercise interventions. The component studies have generally screened out people who have specific diseases, such as type 2 diabetes or cancer, in their recruitment processes. Therefore, these groups are not represented in the data and may have different levels of energy expenditure and food intake from those predicted here. In addition, we further eliminated data relating to individuals engaged in unusual levels of physical activity (for example, participants in the Race Across America^45^ or individuals climbing Mount Everest^46^), measurements of amateur or professional athletes (for example, professional footballers^47^ and jockeys^48^) and females who were pregnant or lactating. We did not eliminate measurements of hunter–gatherer^49^ and subsistence agriculture populations^50^ as evidence suggests that these do not differ from westernized populations in their energy expenditures, once normalized for body weight. However, such measurements comprised less than 1% of the total and their inclusion or exclusion does not materially alter the predictive equations. In total, we had measurements for 7,441 individuals that met all the inclusion criteria.

The data in the database were all recalculated using a common equation that was shown in validation against chamber calorimetry to provide the most accurate and precise measure of CO_2_ production^21^. These estimates were converted to TEE using the modified Weir equation^51^ with either a known food quotient, a measured respiratory quotient derived from 24 h chamber calorimetry or, in the absence of other information, an assumed RQ of 0.85. An initial analysis suggested that deriving a common equation that covered all age classes had a high level of residual error. The structure of the residuals showed that most error was incurred among the youngest participants. We therefore restricted the final analysis to individuals aged ≥4 years. In total, for this age group, we had 6,497 measurements available. We assigned random numbers between 0 and 10,000 to the measurements and then sorted them in order of increasing random number. We then selected the first 90% of measures (*n* = 5,899) as the analysis set and retained the remainder as a validation set (*n* = 598). These data were derived principally from the USA and Western Europe (87.8%), measured mostly since 2000, with lesser contributions from other countries. They are dominated by white (56.5%) and African American (15%) ethnic groups, with lesser contributions by Hispanic, African and Asian ethnic groups (all ethnicities by self-report). We included the elevation of the study location, but did not include ambient temperature during the measurement period because a previous analysis has shown that this is not a significant predictor, at least for data from the USA^26^. Moreover, this is not generally available for survey work. We did not use date of measurement despite recently showing that TEE has declined over time in the USA and Europe in adults^52^ because the current data include children between the ages of 4 and 16 as well as data from additional countries where this relationship to time does not necessarily apply. Moreover, we cannot be sure that this trend will continue into the future.

We combined the TEE measurements with additional information that can be routinely measured in survey work without the need for complex equipment. These extra variables (with the measurement units) were body mass (kg), height without shoes (cm), self-identified sex (m/f), age (yr) and self-reported ethnicity. Ethnicity included African, African living outside Afirca, Asian, white, Hispanic and not-available (10.4%). A small number of individuals identified as mixed race or ‘other’ (2.9%) and these were all coded as ‘not available’ as there were insufficient data to include different combinations separately. We are aware of the discussions regarding the inclusion of ethnicity into analyses of this type and of the history of their misuse in medicine and biology. By including self-reported ethnicity, we do not intend to imply that there is any fundamental physiological or genetic basis to these differences, or that any particular group has ‘superior metabolism’ compared to others. We emphasize that these are self-declared ethnicities and not attributed. If self-declared ethnicity was unavailable in a particular survey or if there were objections for whatever reason to the use of ethnicity as part of the prediction model, then the default was to use ‘not available’, which has a coefficient approximating to 0.

Because the relationship between body mass and TEE follows a power law^25^, we log-converted TEE and body weight before analysis. We log-transformed other variables such as elevation because they were not normally distributed. Moreover, as there is a curvilinear relationship of the normalized TEE with age, we included both age and age^2^ as predictors. We then fitted a generalized linear model to the data using the statistical program Minitab (v19), including all of the primary variables and all of the interaction terms (up to three way). We refined the model by retrospectively deleting non-significant terms, starting with the three-way interactions, and then non-significant two-way interactions. Seventy sets of data were eliminated because of incomplete predictor data (all missing the elevation of the measurement site). We plotted the residual variation against the original predictors to assess whether there was any bias in the predictions (Supplementary Fig. 1). This suggested that the predictors were not biased. Predicted TEE might not be a good estimator of energy intake if individuals are changing weight during the measurement period. That is because when individuals are gaining weight they may be consuming more than they expend, and vice versa when they are losing weight. However, there was also no significant relationship between weight change during the measurement period and the TEE (Supplementary Fig. 2a), suggesting that this did not compromise the predictions. This could be because the majority of weight difference over the 2-week measurement period is not stored energy (for example, most of it is water and perhaps differences in gut fill) and that the remaining energy storage is relatively small compared with total expenditure over a 2-week interval. We did not have information on weight change over longer periods to evaluate whether that influenced the measurements. A recent study found that eliminating individuals who had greater than 5% weight change over the 6 months preceding the TEE measure attenuated the relationship between TEE and all-cause mortality^53^. Childhood growth might also affect the assumption that TEE is equal to energy intake. If we take the extreme example of a rapidly growing adolescent gaining 10 kg per year, that would be equivalent to 0.38 kg over a typical 14-day DLW measurement. If we assume that this mass comprised 65% water, 20% lean tissue and 15% fat, then the extra energy intake above expenditure to deposit this tissue would be about 0.3 MJ, or about 3% of energy expenditure. The direction of this discrepancy would push participants towards over-reporting.

### Validation

We compared the predicted TEE with the observed TEE for the randomly selected 598 data in the validation dataset (Supplementary Fig. 2a). There was a strong correspondence between the observations and the predictions (*R*^2^ = 0.67), and 94.6% of the observations were within the 95% PI of the corresponding predictions. The average absolute deviation between the prediction and observation in this validation set was 11.2%. In addition, we explored whether the predictions from the equation might be valid for other groups not involved in the derivation of the equations, specifically athletes, individuals engaged in unusual activity and reproductive females. In all cases, the observed expenditures of these special groups exceeded the predictions. The average discrepancy across all of the athletes was 8.9 MJ d^−1^ (SD = 1.59) and across all of the reproductive females was 8.04 MJ d^−1^ (SD = 1.50). A more detailed breakdown is provided in Supplementary Table 6. This confirms that the prediction equation cannot be used in these unusual cases.

### Sensitivity analysis

Survey work may not always have all the data available on which to make a prediction. We considered the impact of not having the elevation of the person’s location and not having the person’s self-reported ethnicity. For the sensitivity to elevation effects, we compared the predicted TEE in the validation set with the predicted TEE using a ‘dummy’ elevation of 100 m. The absolute error in the predicted TEE by using the dummy elevation in the validation dataset was 2.3%. We also explored the impact of not knowing the ethnicity on the predicted total energy expenditure (TEE). The ethnic category ‘not available’ was used as a standard to calculate the impact of knowing or not a person’s ethnicity. The change in predicted TEE by knowing the person’s ethnicity compared with ‘not available’ was 2.29% for white, −4.17% for African living in Africa, 1.59% for African living outside Africa, 0.27% for Asian, −1.9% for Hispanic and −0.36% for ‘other’. In general, these errors were small relative to the predictive interval, but clearly having a complete predictor dataset provides a better prediction than incomplete data.

### Machine learning approaches

We used three different machine learning approaches to analyse the data using the same predictor variables: Random Forest, XGBoost and Support Vector Regression. Random Forest is a model that uses multiple trees to train and predict samples. It builds multiple unrelated decision trees by randomly drawing samples and features to obtain predictions in parallel. Each decision tree yields a prediction from the samples and features drawn, and the regression prediction for the whole forest is obtained by combining the results of all the trees and taking the average. Features are randomly selected as the subset of features to be selected when building the tree. Random forests are resistant to overfitting and do not require feature selection. However, as Random Forest does not give continuous output values, it may not be as effective in solving regression problems as it is for solving classification problems. Moreover, if the noise level in the data is high, the performance of Random Forest may decay. XGBoost is a machine learning library that focuses on gradient-boosting algorithms. It was created in 2014 and has attracted much attention for its excellent learning results and efficient training speed. The XGBoost regression that we used is an optimization algorithm for Gradient Boosting Decision Tree (GBDT) regression. GBDT works by training a tree using the training set and the true values, then using this tree to predict the training set and obtain the predicted values for each sample. Hence, we obtained the residual, which was the difference between the true values and prediction. We can then train a second tree, at which point the true value is no longer used, but the residual is used as the standard answer. Once the two trees are trained, the residuals can be obtained again for each sample, then a third tree is further trained, and so on. In short, the GBDT will learn the residual based on previously built trees in each step. We can artificially specify the total number of trees or monitor certain parameters to stop the training procedure. XGBoost improves the GBDT by adding regularization, parallel processing and built-in cross-validation. XGBoost can automatically handle missing values of samples and it is much more stable than Random Forest. It also has the advantages of being highly flexible, efficient in execution and less prone to overfitting. One of its more significant disadvantages from our point of view is the very large number of parameters that can be tuned, making it more challenging to tune parameters in practice to activate the full potential of XGBoost. Support Vector Regression (SVR) is a vital application branch of Support Vector Machines (SVMs) and the basic idea behind it is to find the line of best fit. Here, we used the epsilon Support Vector Regression (Epsilon-SVR) to do the prediction. The advantages of SVR are its low computational complexity, robustness to outliers and excellent generalization ability. However, its disadvantages are that it is not suitable for large datasets and we found in experiments that the preprocessing procedures, such as standardizing, strongly influenced its performance. This makes SVR less easy to use than other methods. See the Code availability statement for details of the source code for the analyses.

### Validation of the machine learning approaches

As detailed above, we used a randomly selected 10% of the original dataset as a validation set. We plotted the predicted energy expenditure from the three machine learning approaches against the actual measured energy expenditure and calculated the summed deviations to evaluate the performance of the different models (Supplementary Fig. 4). In all cases, there was a strong correlation between the predictions and the observations. The summed deviations were very similar between the different approaches, with the average absolute percentage error in the prediction being 11.6% for Random Forest, 11.4% for XGBoost and 11.5% for SVR. These are all very similar to the mean absolute error derived using the classical general linear modelling (11.2%). We then looked at the correlation of the deviations between predicted and actual data for all of the methods (the correlation matrix in Supplementary Table 1). This showed that all the approaches had correlations with the observation that were almost identical (*R* = 0.82) and the deviations between each method and the observation were very strongly correlated with each other (*R* = 0.96–0.99). In effect, the approaches were all extracting the same predictive information from the data. The error of around 11% independent of the approach exceeds the analytical error in the DLW method using the equation that we employed, which is 7.7% (ref. ^21^). There is consequently a gap of unexplained variation that may be possible to explain and refine the predictions. However, the similarities in the different analytical models suggest that additional predictor variables would be required to improve the model predictions.

### Application to previous survey work

The NDNS is a UK government-commissioned rolling programme funded by Public Health England and the UK Food Standards Agency. The rolling programme from 2008 to 2019 is a continuous cross-sectional survey that assesses the diet, nutritional status and nutrient intake of individuals in the UK (England, Scotland, Wales and Northern Ireland) living in private households. The survey aims to collect around 1,000 samples each year, equally divided into 500 children and 500 adults, children aged 1.5–18 years and adults aged 19 years and over. There are two main stages of the survey, namely, interviewer visits and a nurse visit; all nutritional data are collected during the interviewer visits. This study used data from years 1–11 (2008–2009 to 2018–2019) for the population aged 4 and over between April 2008 and August 2019. The total number of eligible individuals included in this study was 12,694.

The NHANES database, used by the Centers for Disease Control and Prevention, contains data from a range of surveys on health and nutrition conducted since the 1960s. These surveys were initially carried out periodically between 1971 and 1994, but since 1999 the surveys have been continuous. Around 5,000 non-institutionalized US civilians are interviewed in their own homes each year and then complete a health examination. The participants fill in a questionnaire regarding their socio-economic, demographic, health-related and dietary information and are then subject to a medical examination that assesses anthropometric and laboratory measurements. A total of 5,873 participants in the NHANES (2017–2018) aged 4–80 were eligible for inclusion in the current study.

In the NDNS, the dietary intake of each participant was assessed through a 4-day food diary that measured their consumption of all foods and beverages during the study period. The respondents filled in information on their diets, including the brands of foods and drinks consumed, portion sizes, ingredients, leftovers, cooking methods and any dietary supplements that they may have taken. A parent or carer was asked to fill in the diary for children under 12 years. Sex refers to the sex of the person eating the food rather than the parent doing the coding. Older children completed the food diary themselves. Editors and coders from the NDNS team were trained to code the dietary intake information and portion sizes, and the 4-day food diaries were analysed using Diet In Nutrient Out, an integrated dietary assessment system, and the Public Health England NDNS Nutrient Databank food composition data^54,55^.

The NHANES nutritional assessment included a 24-hour dietary recall interview with respondents across a range of ages. The assessments were carried out by a trained interviewer who was fluent in English and Spanish. A private room that contained a standard set of measurement guides was used for this first interview. These guides were used to assist the participants in estimating and reporting the portion sizes of their consumed foods. The measuring guides were specially designed to be used in the NHANES setting with a sample population of US civilian participants. A second dietary interview was carried out with all participants via a phone call within 3–10 days of the first interview. Spoons, measuring cups, rulers and food model booklets containing drawings of the measurement guides were provided for the participants to help them more accurately report their portion sizes during the telephone interview. Participants aged 12 years and older were able to record their intake without an assistant. For younger persons, sex refers to the sex of the person eating the food rather than the assistant doing the coding. Food and beverages consumed were coded using the US Department of Agriculture’s Food and Nutrient Database for Dietary Studies to process individuals intake (http://www.ars.usda.gov/nea/bhnrc/fsrg and https://www.cdc.gov).

Both nutritional datasets were screened to remove incomplete participant data and entered into the master spreadsheet so that the DLW equation could be applied. Children under the age of 4 were excluded and the cut-off age for classification as children was 16 years. Ethnicity data were classified according to specific categories: white, African, African living outside Africa, Asian, Hispanic, other and not available. The elevation of location was set as the average of data in the DLW dataset (158.5 m). The equation to calculate predicted TEE was applied. The tolerance interval was determined to calculate the upper and lower level of TEE to provide the accepted range within which daily energy intake must fall. Moreover, differences between energy intake and predicted energy expenditure were calculated and compared with the age and BMI of participants. Data on macronutrients (carbohydrate, protein, fat and alcohol) were converted to kilojoules and percentage of the total energy intake to compare individuals’ consumption with the value of differences between energy intake and expenditure. Dietary intake from NHANES for both the first and second 24-h recall were used separately to determine whether there was an improvement in reporting with greater familiarity of the survey protocol. Then an average for both recalls was calculated and compared with the estimated energy intake. In addition, the participants of NDNS who completed all 4 days of the dietary intake survey were used to assess whether there was an improvement with time when repeating their energy intake survey.

### Statistical analysis of the NDNS and NHANES datasets

Descriptive statistics for socio-demographic variables such as the mean and SD were conducted to describe both included males and females from the NDNS and NHANES participants. Data were further split into adults and children with a cut-off age of 16 years. The full DEE prediction equation will not be disclosed until publication. We calculated the number of participants whose energy intake fell within the expected variation around predicted energy expenditure. We then assessed whether there were differences in the ability to self-report energy intake with increasing age and BMI using linear regression. In addition, multiple regression was used to determine whether different dietary macronutrients (carbohydrate, protein and fat) were more likely to be under-reported. Statistical analyses were conducted using R (v4.1.3)^56^ and Minitab (v19) and *p* ≤ 0.05 was considered statistically significant.

## Supplementary information

Supplementary Information Supplementary Methods, Figs. 1–4, Tables 1–7 and Contributing non-authors.

## Data Availability

All of the data used in the derivation of the regression model are freely available via the IAEA DLW Database at https://doubly-labelled-water-database.iaea.org/home and www.dlwdatabase.org. Access to the full database must be made via an online application, but a subsample is available without restriction for free download. The NDNS data are subject to restrictions and are not available to the public. Requests to access these datasets should be directed to https://ukdataservice.ac.uk/. The NHANES data are freely available at https://wwwn.cdc.gov/nchs/nhanes/continuousnhanes/default.aspx?BeginYear=2017.

## Code availability

The source code for the Python analysis is available in the Supplementary Information.
