# Author Correction: Predictive equation derived from 6,497 doubly labelled water measurements enables the detection of erroneous self-reported energy intake

**DOI:** 10.1038/s43016-025-01175-2

**Published:** 2025-04-23

**Authors:** Rania Bajunaid, Chaoqun Niu, Catherine Hambly, Zongfang Liu, Yosuke Yamada, Heliodoro Aleman-Mateo, Liam J. Anderson, Lenore Arab, Issad Baddou, Linda Bandini, Kweku Bedu-Addo, Ellen E. Blaak, Carlijn V. C. Bouten, Soren Brage, Maciej S. Buchowski, Nancy F. Butte, Stefan G. J. A. Camps, Regina Casper, Graeme L. Close, Jamie A. Cooper, Richard Cooper, Sai Krupa Das, Peter S. W. Davies, Prasangi Dabare, Lara R. Dugas, Simon Eaton, Ulf Ekelund, Sonja Entringer, Terrence Forrester, Barry W. Fudge, Melanie Gillingham, Annelies H. Goris, Michael Gurven, Asmaa El Hamdouchi, Hinke H. Haisma, Daniel Hoffman, Marije B. Hoos, Sumei Hu, Noorjehan Joonas, Annemiek M. Joosen, Peter Katzmarzyk, Misaka Kimura, William E. Kraus, Wantanee Kriengsinyos, Rebecca Kuriyan, Robert F. Kushner, Estelle V. Lambert, Pulani Lanerolle, Christel L. Larsson, William R. Leonard, Nader Lessan, Marie Löf, Corby K. Martin, Eric Matsiko, Anine C. Medin, James C. Morehen, James P. Morton, Aviva Must, Marian L. Neuhouser, Theresa A. Nicklas, Christine D. Nyström, Robert M. Ojiambo, Kirsi H. Pietiläinen, Yannis P. Pitsiladis, Jacob Plange-Rhule, Guy Plasqui, Ross L. Prentice, Susan B. Racette, David A. Raichlen, Eric Ravussin, Leanne M. Redman, John J. Reilly, Rebecca Reynolds, Susan B. Roberts, Dulani Samaranayakem, Luis B. Sardinha, Analiza M. Silva, Anders M. Sjödin, Marina Stamatiou, Eric Stice, Samuel S. Urlacher, Ludo M. Van Etten, Edgar G. A. H. van Mil, George Wilson, Jack A. Yanovski, Tsukasa Yoshida, Xueying Zhang, Alexia J. Murphy-Alford, Srishti Sinha, Cornelia U. Loechl, Amy H. Luke, Herman Pontzer, Jennifer Rood, Hiroyuki Sagayama, Dale A. Schoeller, Klaas R. Westerterp, William W. Wong, John R. Speakman

**Affiliations:** 1 School of Biological Sciences, University of Aberdeen, Aberdeen, UK; 2 Food and Nutrition Department, King Abdulaziz University, Jeddah, Saudi Arabia; 3 Shenzhen Key Laboratory of Metabolic Health, Center for Energy Metabolism and Reproduction, Shenzhen Institute of Advanced Technology, Chinese Academy of Sciences, Shenzhen, China; 4 State Key Laboratory of Molecular Developmental Biology, Institute of Genetics and Developmental Biology, Chinese Academy of Sciences, Beijing, China; 5 Graduate School of Biomedical Engineering, Tohoku University, Sendai, Japan; 6 Department of Medicine and Science in Sports and Exercise, Graduate School of Medicine, Tohoku University, Sendai, Japan; 7 Department of Nutrition and Metabolism, Nutrition Coordination, Research Center for Food and Development (CIAD), Hermosillo, Mexico; 8 School of Sport, Exercise and Rehabilitation Sciences, University of Birmingham, Birmingham, UK; 9 David Geffen School of Medicine, University of California, Los Angeles, CA USA; 10 Unité Mixte de Recherche en Nutrition et Alimentation, CNESTEN-Université Ibn Tofail URAC39, Regional Designated Center of Nutrition Associated with AFRA/IAEA, Rabat, Morocco; 11 University of Massachusetts Chan Medical School, Worcester, MA USA; 12 Department of Physiology, Kwame Nkrumah University of Science and Technology, Kumasi, Ghana; 13 Department of Human Biology, NUTRIM, School for Metabolism and Translational Research in Metabolism, Maastricht University Medical Centre+, Maastricht, The Netherlands; 14 MRC Epidemiology Unit, University of Cambridge, Cambridge, UK; 15 Division of Gastroenterology, Hepatology and Nutritiion, Department of Medicine, Vanderbilt University, Nashville, TN USA; 16 Department of Pediatrics, Baylor College of Medicine, USDA/ARS Children’s Nutrition Research Center, Houston, TX USA; 17 Department of Psychiatry and Behavioral Sciences, Stanford University School of Medicine, Stanford, CA USA; 18 Research Institute for Sport and Exercise Sciences, Liverpool John Moores University, Liverpool, UK; 19 Nutritional Sciences, University of Wisconsin, Madison, WI USA; 20 Department of Public Health Sciences, Parkinson School of Health Sciences and Public Health, Loyola University, Maywood, IL USA; 21 USDA Human Nutrition Research Center on Aging at Tufts University, Boston, MA USA; 22 Child Health Research Centre, Centre for Children’s Health Research, University of Queensland, South Brisbane, Queensland Australia; 23 Department of Physiotherapy, Faculty of Allied Health Sciences, General Sir John Kotelawala Defence University, Kandawala, Sri Lanka; 24 Division of Epidemiology and Biostatistics, School of Public Health, University of Cape Town, Cape Town, South Africa; 25 Developmental Biology and Cancer Department, UCL Great Ormond Street Institute of Child Health, London, UK; 26 Department of Sport Medicine, Norwegian School of Sport Sciences, Oslo, Norway; 27 Charité–Universitätsmedizin Berlin, corporate member of Freie Universität Berlin and HumboldtUniversität zu Berlin, Institute of Medical Psychology, Berlin, Germany; 28 Department of Pediatrics, School of Medicine, University of California Irvine, Irvine, CA USA; 29 Solutions for Developing Countries, University of the West Indies, Kingston, Jamaica; 30 Department of Biomedical and Life Sciences, University of Glasgow, Glasgow, UK; 31 Department of Molecular and Medical Genetics, Oregon Health and Science University, Portland, OR USA; 32 Imec, OnePlanet Research Center, Wageningen, The Netherlands; 33 Department of Anthropology, University of California Santa Barbara, Santa Barbara, CA USA; 34 Population Research Centre, Faculty of Spatial Sciences, University of Groningen, Groningen, The Netherlands; 35 Department of Nutritional Sciences, Program in International Nutrition, Rutgers University, New Brunswick, NJ USA; 36 Central Health Laboratory, Ministry of Health and Wellness, Candos, Mauritius; 37 Pennington Biomedical Research Center, Baton Rouge, LA USA; 38 Institute for Active Health, Kyoto University of Advanced Science, Kyoto, Japan; 39 Department of Medicine, Duke University, Durham, NC USA; 40 Institute of Nutrition, Mahidol University, Nakhon-Pathom, Thailand; 41 Division of Nutrition, St. John’s Research Institute, Bangalore, India; 42 Feinberg School of Medicine, Northwestern University, Chicago, IL USA; 43 Health through Physical Activity, Lifestyle and Sport Research Centre (HPALS), Division of Exercise Science and Sports Medicine (ESSM), FIMS International Collaborating Centre of Sports Medicine, Department of Human Biology, Faculty of Health Sciences, University of Cape Town, Cape Town, South Africa; 44 Department of Biochemistry and Molecular Biology, Faculty of Medicine, University of Colombo, Colombo, Sri Lanka; 45 Department of Food and Nutrition and Sport Science, University of Gothenburg, Gothenburg, Sweden; 46 Department of Anthropology, Northwestern University, Evanston, IL USA; 47 Imperial College London Diabetes Centre, Abu Dhabi, United Arab Emirates; 48 Department of Metabolism, Digestion and Reproduction, Imperial College London, London, UK; 49 Department of Health, Medicine and Caring Sciences, Linköping University, Linköping, Sweden; 50 Department of Biosciences and Nutrition, Karolinska Institute, Stockholm, Sweden; 51 UR Sweden Program, University of Rwanda, Kigali, Rwanda; 52 Department of Nutrition, Institute of Basic Medical Sciences, University of Oslo, Oslo, Norway; 53 Department of Nutrition and Public Health, Faculty of Health and Sport Sciences, University of Agder, Kristiansand, Norway; 54 Tufts University School of Medicine, Boston, MA USA; 55 Division of Public Health Sciences, Fred Hutchinson Cancer Center and School of Public Health, University of Washington, Seattle, WA USA; 56 Kenya School of Medicine, Moi University, Eldoret, Kenya; 57 Rwanda Division of Basic Sciences, University of Global Health Equity, Kigali, Rwanda; 58 Obesity Research Unit, Research Program for Clinical and Molecular Metabolism, Faculty of Medicine, University of Helsinki and Abdominal Center, Obesity Center, HealthyWeightHub, Helsinki University Hospital and University of Helsinki, Helsinki, Finland; 59 School of Sport and Service Management, University of Brighton, Eastbourne, UK; 60 Department of Nutrition and Movement Sciences, Maastricht University, Maastricht, The Netherlands; 61 College of Health Solutions, Arizona State University, Phoenix, AZ USA; 62 Biological Sciences and Anthropology, University of Southern California, California, CA USA; 63 School of Psychological Sciences and Health, University of Strathclyde, Glasgow, UK; 64 Centre for Cardiovascular Sciences, Queen’s Medical Research Institute, University of Edinburgh, Edinburgh, UK; 65 Department of Medicine and Department of Epidemiology, Geisel School of Medicine at Dartmouth College, Hanover, NH USA; 66 Department of Community Medicine, Faculty of Medicine, University of Colombo, Colombo, Sri Lanka; 67 Exercise and Health Laboratory, CIPER, Faculdade Motricidade Humana, Universidade de Lisboa, Lisbon, Portugal; 68 Department of Nutrition, Exercise and Sports, Copenhagen University, Copenhagen, Denmark; 69 Department of Psychiatry and Behavioural Sciences, Stanford University, Stanford, CA USA; 70 Department of Anthropology, Baylor University, Waco, TX USA; 71 Child and Brain Development Program, CIFAR, Toronto, Ontario Canada; 72 Maastricht University, Brightlands Campus Greenport Venlo and Lifestyle Medicine Center for Children, Jeroen Bosch Hospital, ’s-Hertogenbosch, The Netherlands; 73 Section on Growth and Obesity, Division of Intramural Research, Eunice Kennedy Shriver National Institute of Child Health and Human Development, National Institutes of Health Bethesda, Bethesda, MD USA; 74 National Institute of Health and Nutrition, National Institutes of Biomedical Innovation, Health and Nutrition, Osaka, Japan; 75 Department of Nutritional Sciences, International Atomc Energy Agency, Vienna, Austria; 76 Duke Global Health Institute, Duke University, Durham, NC USA; 77 Department of Evolutionary Anthropology, Duke University, Durham, NC USA; 78 Faculty of Health and Sport Sciences, University of Tsukuba, Ibaraki, Japan; 79 Biotechnology Center and Department of Nutritional Sciences, University of Wisconsin, Madison, WI USA; 80 Institute of Health Sciences, China Medical University, Shenyang, China

**Keywords:** Obesity, Epidemiology

Correction to: *Nature Food* 10.1038/s43016-024-01089-5, published online 13 January 2025.

In the version of this article initially published, there was an error in the application of the equation used to predict the total energy expenditure of individuals in the NDNS and NHANES surveys. The output of the equation is in MJ, the two survey intake estimates are in kJ, and while the predicted values were converted to kJ, the value for total energy expenditure, using kJ, was mistakenly inserted into the equations used to derive the extent of over- and underreporting, resulting in an overestimation of underreporting. For instance, the original abstract reported that the level of misreporting was >50%, whereas it is now determined to be 27.4%.

The corrected calculations led to revisions throughout the text, Tables 2–4, Figs. 2 and 3, and the Supplementary Information. Other minor errors were also corrected in this new version. For transparent comparison, the original, uncorrected article is available alongside this amendment. The changes have been made in the HTML and PDF versions of the article. The authors sincerely apologise for these errors and any inconvenience caused. Please direct any correspondence to John R. Speakman.

## Supplementary information

Supplementary Information Original, uncorrected article and Supplementary information.

