# Supplementary material for: Predictive equation derived from 6,497 doubly labelled water measurements enables the detection of erroneous self-reported energy intake
Source: Nat Food. 2025 Jan 13;6(1):58–71. doi: 10.1038/s43016-024-01089-5 (PMC11772230; doi:10.1038/s43016-024-01089-5)
Supplement: Supplementary file 1 — Supplementary Methods, Figs. 1–4, Tables 1–7 and Contributing non-authors. [file 43016_2024_1089_MOESM1_ESM.pdf]

nature food

Supplementary information https://doi.org/10.1038/s43016-024-01089-5

Predictive equation derived from 6,497 doubly labelled water measurements enables the detection of erroneous self-reported energy intake

In the format provided by the authors and unedited

Table of Contents

Supplementary Tables 2

Supplementary Figures 11

Supplementary Methods 15

Contributing non-authors 19

Supplementary Tables

Table S1: Correlation matrix of the predictions against the observed data using the four different analytical approaches and the correlations between the deviations of the predictions from the actual observations across the four different analytical approaches.

	Multi Linear	Random Forest	XGBoost	SVM	Observed
Multi Linear	1	0.953468	0.976156	0.946645	0.815885
Rand Forest	0.953468	1	0.980513	0.904719	0.819497
XGBoost	0.976156	0.980513	1	0.926833	0.820515
SVM	0.946645	0.904719	0.926833	1	0.760951
Observed	0.815885	0.819497	0.820515	0.760951	1

Table S2. Descriptive statistics: sociodemographic characteristics of the study participants

		NDNS		NHANES	
**Age**		**Mean**	**±SD**	**Mean**	**±SD**
	**male**	30.8	23.3	39.5	23.7
	**female**	33.9	23.2	39.1	23.2
**Sex**		**N**	**%**	**N**	**%**
	**male**	5799	45.7	2819	48
	**female**	6895	54.3	3054	52.0
**BMI**		**Mean**	**±SD**	**Mean**	**±SD**
	**male**	23.605	6.135	26.991	7.436
	**female**	24.4	6.5	28.	8.7
**Ethnicity**		**N**	**%**	**N**	**%**
**White**		11914	93.86	2052	34.94
**African heritage persons living outside Africa**		-	-	1444	24.59
**African living in Africa**		-	-	-	-
**Asian**		600	4.73	680	11.58
**Hispanic**		-	-	1310	22.31
**Other**		180	1.42	387	6.59
**Not available**		-	-	-	-

SD= standard deviation, N= total number, %= percentage, BMI (Body Mass Index)

Table S3: The number of participants classified as under and over reporters% in the NDNS dataset years (1-11) and NHANES datasets year (2017-2018) using Goldberg, Black, and McCrory equations.

	NDNS				NHANES			
	Goldberg cut-off1				Goldberg cut-off1			
	Under%				Under%			
sex	M	F			M	F		
Day1	13.52	14.73			14.62	16.35		
Day2	14.97	14.97			21.36	22.54		
Day3	14.97	17.15						
Day 4	16.83	18.16						
	Goldberg cut-off 2				Goldberg cut-off 2			
Average days	33.89	37.9			20.37	22.44		
	Black				Black			
	Under%		Over%		Under%		Over%	
sex	M	F	M	F	M	F	M	F
Day1	19.93	21.81	0.32	0.19	20.19	22.41	3.37	2.10
Day2	21.28	22.83	0.35	0.24	27.86	29.16	2.02	1.41
Day3	22.12	23.94	0.26	0.30				
Day4	23.69	25.42	0.33	0.41				
Average days	29.84	33.33	0.04	0.03	25.87	28.44	2.98	1.80
	McCrory cut-off1 (1sd)				McCrory cut-off1 (1 sd)			
	Under%				Under%			
sex	M	F			M	F		
Day1	57.25	61.09			46.73	52.92		
Day2	57.64	60.62			55.27	60.95		
Day3	58.33	60.66						
Day4	60.52	62.87						
Average days	67.06	70.72			55.65	64.12		
	McCrory cut-off2 (2 sd)				McCrory cut-off2 (2 sd)			
	Under%				Under%			
sex	M	F			M	F		
Day1	11.22	13.26			10.72	14.84		
Day2	11.45	13.9			16.76	21.40		
Day3	11.29	14.52						
Day4	12.86	16.44						
Average days	17.99	21.19			19.3	24.63		

Table S4A: Comparison between DEE calculated using the DLW equation and self-reported DEI in KJ in the NDNS dataset years (1-11) for subjects who completed 4 days. Energy intake refers to the intake on the particular comparison day.

Days		Averaged Energy intake	within range (N)	within range (%)
1 day	Male children	7338.59	2082	84.67
	Male adult	8655.91	2073	63.88
	Female children	6450.81	1861	79.70
	Female adult	6715.27	2702	61.03
2 day	Male children	7168.35	1994	81.09
	Male adult	8660.30	2028	62.50
	Female children	6339.73	1832	78.46
	Female adult	6669.65	2729	61.64
3 day	Male children	7094.99	1970	80.11
	Male adult	8623.95	2043	62.96
	Female children	6186.92	1763	75.50
	Female adult	6670.63	2690	60.76
4 day	Male children	6976.02	1931	78.53
	Male adult	8420.57	1956	60.28
	Female children	6136.06	1740	74.52
	Female adult	6533.40	2546	57.51

Table S4B: Comparison between DEE calculated using the DLW equation and self-reported DEI in KJ in the NDNS dataset years (1-11) for subjects who completed 4 days using averaged intake for days. Average intake refers to the average intake over the given day and all previous survey days.

Averaged days		Averaged Energy intake	within range (N)	within range (%)
1 day	Male children	7338.59	2082	84.67
	Male adult	8655.91	2073	63.88
	Female children	6450.81	1861	79.70
	Female adult	6715.27	2702	61.03
2 days	Male children	7253.47	2110	85.81
	Male adult	8658.10	2149	66.22
	Female children	6395.27	1927	82.53
	Female adult	6692.46	2838	64.10
3 days	Male children	7200.64	2116	86.05
	Male adult	8646.72	2174	67.00
	Female children	6325.82	1918	82.14
	Female adult	6685.19	2880	65.16
4 days	Male children	7144.49	2116	86.05
	Male adult	8590.18	2196	67.67
	Female children	6278.38	1895	81.16
	Female adult	6647.24	2854	64.47

Supplementary Table S5: Comparison between DEE calculated using the DLW equation and self-reported DEI in KJ in the NHANES dataset year (2017-2018) for subjects who completed 24-hour recalls and averaged two recalls.

Days		Averaged Energy intake	within range (N)	within range (%)
1 day	Male children	8754.90	543	76.48
	Male adult	10149.91	1358	64.39
	Female children	7335.42	607	80.50
	Female adult	7668.29	1575	68.47
2 day	Male children	7721.85	500	70.42
	Male adult	9184.49	1205	57.14
	Female children	6846.01	573	75.99
	Female adult	6922.25	1404	61.04
Average 2 days	Male children	8238.38	562	79.15
	Male adult	9667.20	1372	65.05
	Female children	7079.49	634	84.08
	Female adult	7295.27	1625	70.65

Table S6: Deviations between the predicted and observed energy expenditures for specific groups not included in derivation of the equation.

**Athletes and usual activity**	**mean diff (MJ/d)**	**sd**	**n**
Race across America participants	11.3	6.71	18
Cross country skiers	17.6	5.97	8
Flat course jockeys	3.34	1.96	15
Male rugby league players	10.7	2.31	6
Male football (soccer) players	4.31	2.62	8
Adolescent track and field amateurs	6.82	2.52	190
**Reproductive females**			
Pregnancy trimester 2	2.53	2.51	21
Pregnancy trimester 3	3.53	2.52	71
Lactation	5.25	3.28	86

Table S7 Example calculations for two hypothetical subjects

Subject 1: Hispanic male living in Boston, USA (elevation 14m). Age 41, weight 85 kgs, 1.83m tall. Self reported intake 12 MJ/day.

variable		coefficient	variable x coefficient	
constant			-0.2172	
primary variables				
Ln (BW) kg	4.442651	0.4167	1.851253	
Height cm	183	0.006565	1.201395	
age y	41	-0.02054	-0.84214	
age2	1681	0.0003308	0.556075	
age3	68921	-0.000001852	-0.12764	
Ln(elevation) m	2.639057	0.09126	0.24084	
Sex	-1	-0.04092	0.04092	
ethnicity				
A	0	0.0194	0	
AA	0	-0.03899	0	
AS	0	0.006238	0	
C	0	0.02626	0	
H	1	-0.0155	-0.0155	
NA	0	0.003598	0	
interactions				
height x Ln(elevation)	482.9475	-0.0006759	-0.32642	
age x Ln(elevation)	108.2014	0.002018	0.21835	
age2 x Ln(elevation)	4436.255	-0.00002262	-0.10035	
sex x Ln(elevation)	-2.63906	-0.006947	0.018334	
summed values			2.497913	
exponent of summed values			12.15709	MJ/day
			2905.615	kcal/day
lower 95% PI			7.535986	MJ/d
upper 95% PI			19.05123	MJ/d

Person reports eating 12 MJ/day in survey. Since this is inside the 95% predictive interval this person would not be classed as misreporting their food intake.

Subject 2; Asian female, living in Aberdeen UK (elevation 39m), 1.51m tall, 22 years old. Self reported intake 4.15 MJ/d

variable		coefficient	variable x coefficient	
constant			-0.2172	
primary variables				
Ln (BW) kg	3.839452	0.4167	1.5999	
Height cm	151	0.006565	0.991315	
age y	22	-0.02054	-0.45188	
age2	484	0.0003308	0.160107	
age3	10648	-0.000001852	-0.01972	
Ln(elevation) m	3.663562	0.09126	0.334337	
Sex	1	-0.04092	-0.04092	
ethnicity				
A	0	0.0194	0	
AA	0	-0.03899	0	
AS	1	0.006238	0.006238	
C	0	0.02626	0	
H	0	-0.0155	0	
NA	0	0.003598	0	
interactions				
height x Ln(elevation)	553.1978	-0.0006759	-0.37391	
age x Ln(elevation)	80.59836	0.002018	0.162647	
age2 x Ln(elevation)	1773.164	-0.00002262	-0.04011	
sex x Ln(elevation)	3.663562	-0.006947	-0.02545	
summed values			2.085358	
exponent of summed values			8.047471	MJ/d
			1923.392	kcal/d
lower 95% PI			4.467742	MJ/d
upper 95% PI			13.54639	MJ/d

Person reports eating 4.15 MJ/day in survey. This is below the lower 95% predictive interval and so this person would be excluded as an under-reporter.

Supplementary Figures

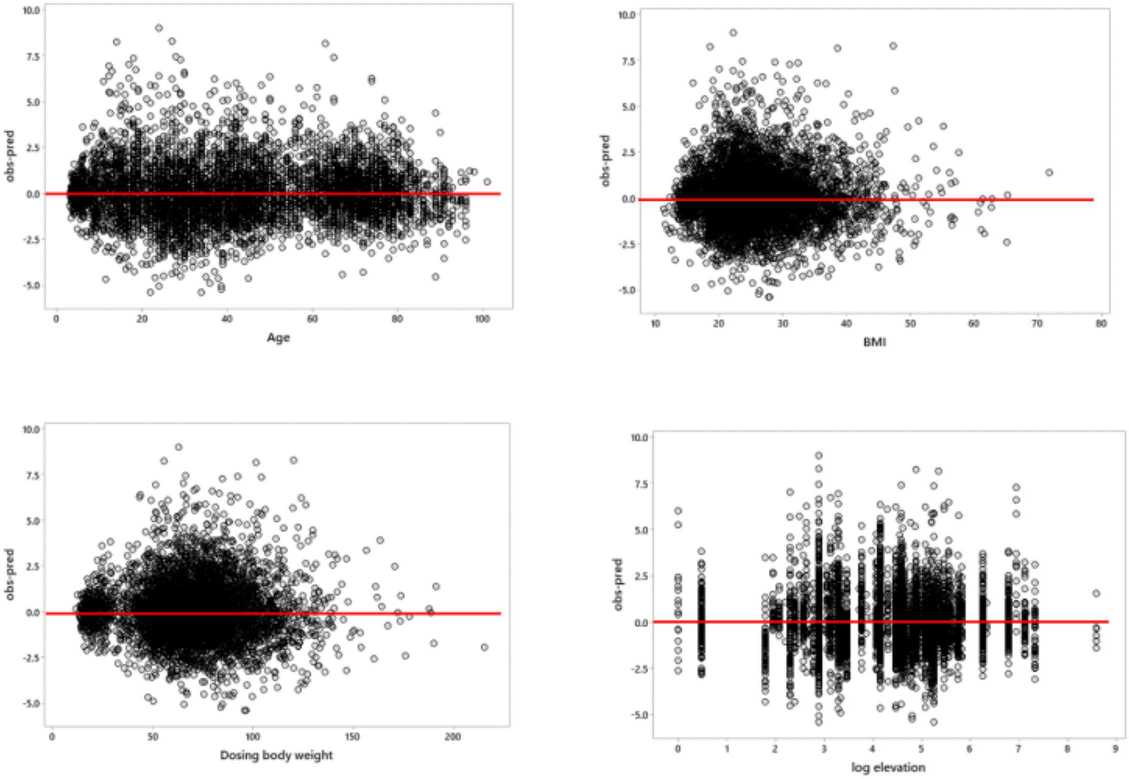

Figure S1 Residual error in the predicted energy expenditure plotted against A: age, B: BMI, C: body weight and D) log elevation for the 5828 data used in the derivation of the prediction. There was no indication of systematic bias with respect to any of the predictor variables. The line of no difference is shown in red.

A

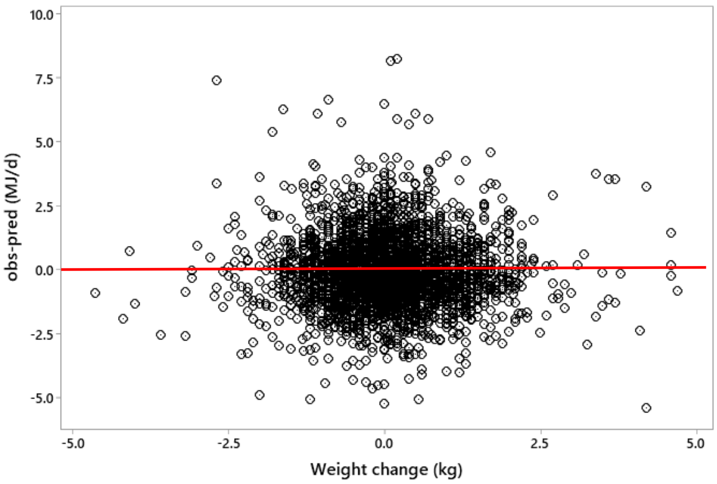

B

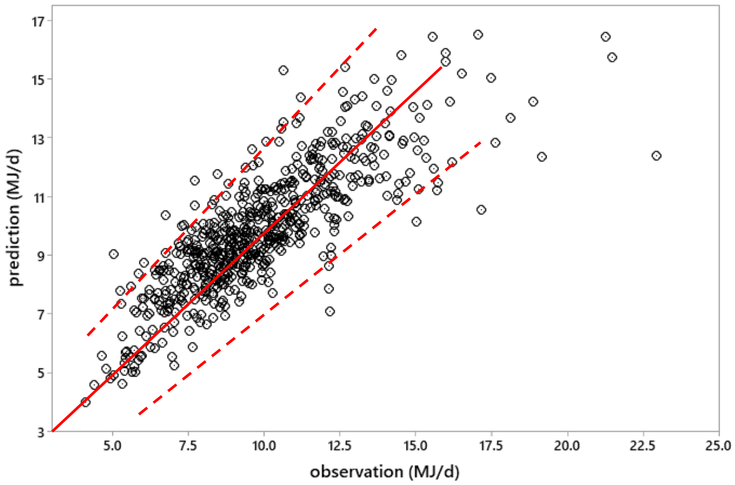

Figure S2: A: Residual error in the predicted energy expenditure plotted against change in body weight over the measurement period for 3088 individuals where weight change was reported. Seventeen individuals changed by more than 5 kgs and are not illustrated in this plot. There was no significant relationship whether these data were included or excluded. Line of no change in weight in red. B: observed TEE (MJ/d) plotted against the predicted TEE from the model for the 598 subjects in the test

data set. The line of identity is shown in red. The upper and lower 95% predictive intervals around the mean are shown dashed. 94.4% of the data fell inside the predictive interval.

'd)

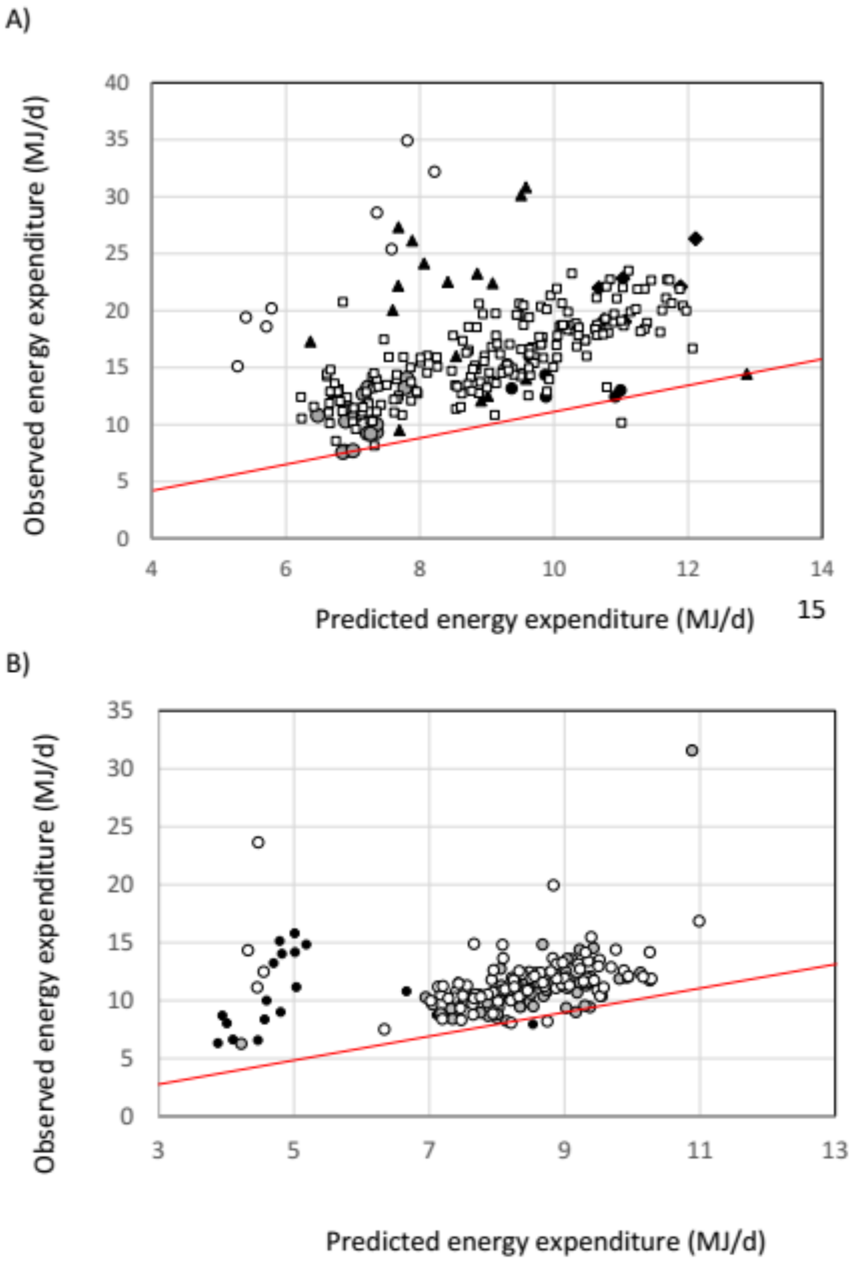

Figure S3: A: predicted and observed TEE for 246 athletes and individuals engaged in usual activities. Open circles cross country skiers, black triangles race across America participants, open squares, Portuguese track and field athletes, black circles male football (soccer) players, black diamonds male rugby league players, grey circles flat course jockeys. Line of identity shown in red. B: predicted and

observed TEE for 176 females in reproduction. Open circles pregnancy trimester 2, grey circles pregnancy trimester 3, black circles, lactation. Lactating sample mostly from rural Africa. Line of identity shown in red.

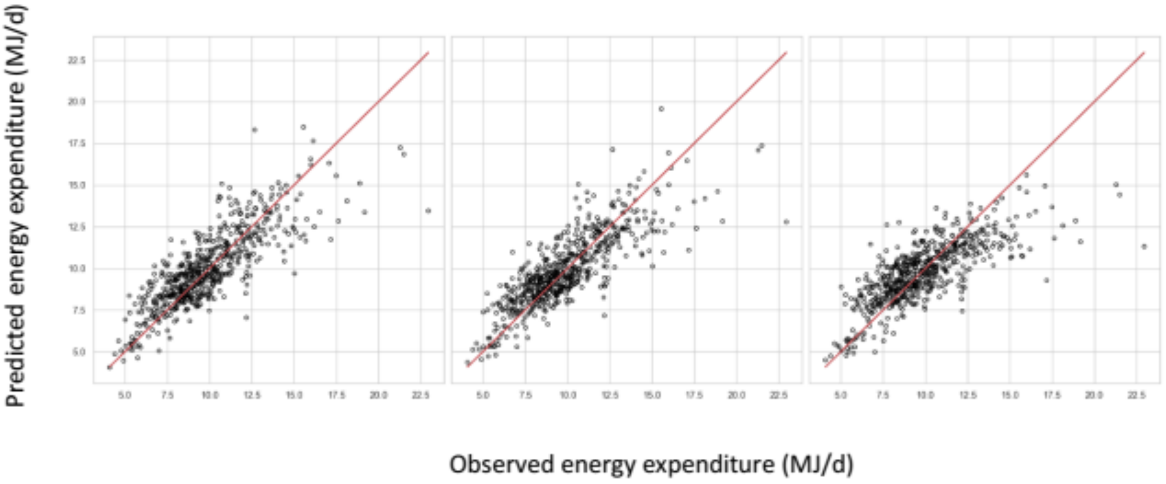

Figure S4: Predicted energy expenditure (MJ/d) from the three machine learning approaches A Random forest, B: XGBoost and C: SVR plotted against the actual observed expenditure (MJ/d) for a sample of 598 randomly selected individuals. The line of identity in each case is shown in red.

Supplementary Methods

Source code for machine learning analysis in Python

Data were pre-processed using the following script

```
#!/usr/bin/env python
# -*- encoding: utf-8 -*-
"""
@File    :   pre_processor.py
@Contact :   zongfangliu00@gmail.com
@License :   (C)Copyright 2022-2023, ZongfangLiu

@Modify Time      @Author    @Version    @Description
------------      -------    --------    -----------
2023/11/14 16:37   zfliu      1.0         None
"""
import pandas as pd


def standardize(all_features):

    numeric_features_index = all_features.dtypes[all_features.dtypes !=
'object'].index

    dic = 15
    for item in all_features[numeric_features_index]:
        dic[item] = (all_features[item].mean(), all_features[item].std())

    all_features[numeric_features_index] =
all_features[numeric_features_index].apply(
        lambda x: (x - x.mean()) / (x.std()))

    all_features[numeric_features_index] =
all_features[numeric_features_index].fillna(0)

    return all_features, dic


def standardize_test(all_features_test, parameter_dic):

    numeric_features_index =
all_features_test.dtypes[all_features_test.dtypes != 'object'].index

    for item in parameter_dic:
        all_features_test[item] = all_features_test[item].apply(
            (lambda x: (x - parameter_dic[item][0]) /
(parameter_dic[item][1])))

    all_features_test[numeric_features_index] =
all_features_test[numeric_features_index].fillna(0)

    return all_features_test
```

```
def one_hot(all_features):

    all_features = pd.get_dummies(all_features, dummy_na=True)

    return all_features


def shuffle(data):
    data = data.sample(frac=1, random_state=42).reset_index(drop=True)
    return data
```

Analysis was completed using the following script.

```
#!/usr/bin/env python
# -*- encoding: utf-8 -*-
"""
@File     :   Tee_data_analysis.py
@Contact :   zongfangliu00@gmail.com
@License :   (C)Copyright 2022-2023, ZongfangLiu

@Modify Time      @Author    @Version    @Description
------------      -------    --------    -----------
2023/11/14 16:37   zfliu      1.0         None
"""
from src.data_processing import pre_processor
import pandas as pd
from sklearn.linear_model import LinearRegression
import xgboost as xgb
from sklearn.ensemble import RandomForestRegressor
from sklearn.svm import SVR
import numpy as np
from matplotlib import pyplot as plt
import seaborn as sns


train_data =
pd.read_csv(r"/Users/liuzongfang/Documents/Codes/TEE_Prediction/codes/TEE_pap
er_exps/data/original_data/train_set.csv",
na_values="NaN")#.values.astype(np.float32)
test_data =
pd.read_csv(r"/Users/liuzongfang/Documents/Codes/TEE_Prediction/codes/TEE_pap
er_exps/data/original_data/test_set.csv",
na_values="NaN")#.values.astype(np.float32)

# Must do one hot
do_shuffle, do_standardize, do_onehot, do_drop = True, False, True, False


if do_drop:
    train_data = train_data.drop(['Sex', 'ethnicity'], axis=1, inplace=False)
    test_data = test_data.drop(['Sex', 'ethnicity'], axis=1, inplace=False)

all_features = train_data.iloc[:, :-1]
TEE = train_data.iloc[:, -1]
all_features_test = test_data.iloc[:, :-1]
TEE_test = test_data.iloc[:, -1]
```

```
if do_standardize:

    all_features, parameter_dic = pre_processor.standardize(all_features)

    all_features_test = pre_processor.standardize_test(all_features_test,
parameter_dic)


if do_onehot:

    all_features = pre_processor.one_hot(all_features)
    all_features_test = pre_processor.one_hot(all_features_test)

# Replace the missing values as mean values
indexes = all_features.columns.values
for item in indexes:
    cur_mean = all_features[item].mean()
    all_features[item].fillna(value=cur_mean, inplace=True)
    cur_mean_test = all_features_test[item].mean()
    all_features_test[item].fillna(value=cur_mean, inplace=True)

train_data = pd.concat((all_features, TEE), axis=1)
test_data = pd.concat((all_features_test, TEE_test), axis=1)

if do_shuffle:

    train_data = pre_processor.shuffle(train_data)
#%%
train_data = train_data.values.astype(np.float32)
test_data = test_data.values.astype(np.float32)
x_train, y_train = train_data[:, :-1], train_data[:, -1]
x_test, y_test = test_data[:, :-1], test_data[:, -1]

random_forest = RandomForestRegressor()
xgboost = xgb.XGBRegressor(
    eta=0.05,
    max_depth=6,
    seed=42,
)
svr = SVR()

random_forest.fit(x_train, y_train)
xgboost.fit(x_train, y_train)
svr.fit(x_train, y_train)

rf_pred = random_forest.predict(x_test)
xgb_pred = xgboost.predict(x_test)
svr_pred = svr.predict(x_test)

sns.set(style='whitegrid')
colors = np.array([0, 0, 0])

fig, [ax0, ax1, ax2] = plt.subplots(1, 3, figsize=(18, 6), sharey=True,
constrained_layout=True)

ax0.scatter(y_test, rf_pred, s=18, facecolors='none', edgecolors=colors)
```

```
ax0.plot(y_test, y_test, 'r')

ax1.scatter(y_test, xgb_pred, s=18, facecolors='none', edgecolors=colors)
ax1.plot(y_test, y_test, 'r')

ax2.scatter(y_test, svr_pred, s=18, facecolors='none', edgecolors=colors)
ax2.plot(y_test, y_test, 'r')

plt.show()

def MAPE(y, y_pred):
    return sum(abs(y - y_pred) / y) / len(y)

mape_values = {}
mape_values["RandomForest"] = MAPE(y_test, rf_pred)
mape_values["Xgboost"] = MAPE(y_test, xgb_pred)
mape_values["SVR"] = MAPE(y_test, svr_pred)
print(mape_values)
```

The IAEA DLW database group authorship (database version 3.7).

This group authorship contains the names of some researchers who did not assent inclusion to the main authorship because they felt their contribution was not sufficient to merit authorship, people whose data were contributed into the IAEA DLW database by the analysis laboratory but they later could not be traced, or they did not respond to emails to assent inclusion among the authorship.

Dr Philip N. Ainslie
Research Institute for Sport and Exercise Sciences, Liverpool John Moores University, Liverpool, UK

Dr Lene F. Andersen
Department of Nutrition, Institute of Basic Medical Sciences, University of Oslo, 0317 Oslo, Norway.

Dr Stephane Blanc
Nutritional Sciences, University of Wisconsin, Madison, WI, USA
Institut Pluridisciplinaire Hubert Curien. CNRS Université de Strasbourg, UMR7178, France.

Dr Alberto G. Bonomi
Maastricht University, Maastricht, The Netherlands

Dr Pascal Bovet
Institute of Social and Preventive Medicine, Lausanne University Hospital, Lausanne, Switzerland.

Dr Stefan Branth
University of Uppsala, Uppsala, Sweden

Dr Niels C. De Bruin
Erasmus University, Rotterdam, The Netherlands

Dr Lisa H. Colbert
Kinesiology, University of Wisconsin, Madison, WI,

Dr Dan Cummings

Dr William Dietz
George Washington University, Washington DC, US

Dr Alice E. Dutman
TNO Quality of Life, Zeist, The Netherlands

Dr Cara B. Ebbeling
Boston Children's Hospital, Boston, Massachusetts, USA

Dr Sölve Elmståhl
Lund University, Lund, Sweden

Dr Mikael Fogelholm
Dept of Food and Nutrition, Helsinki, Finland

Dr Tamara Harris
Aging, NIH, Bethesda, MD,

Dr Rik Heijligenberg
Academic Medical Center of Amsterdam University, Amsterdam, The Netherlands

Dr Hans U. Jorgensen
Bispebjerg Hospital, Copenhagen, Denmark

Dr Kitty P. Kempen
Maastricht University, Maastricht, The Netherlands

Dr David S. Ludwig
Boston Children's Hospital, Boston, Massachusetts, USA

Dr Margaret McCloskey
Royal Belfast Hospital for Sick Children, Belfast, Northern Ireland

Dr Erwin P. Meijer
Phillips Research, Eindhoven, The Netherlands

Dr Gerwin A. Meijer
Maastricht University, Maastricht, The Netherlands

Dr Daphne L. Pannemans
Maastricht University, Maastricht, The Netherlands

Dr Renaat M. Philippaerts
Katholic University Leuven, Leuven, Belgium

Dr Roberto A. Rabinovich
The Queen’s medical research Institute, University of Edinburgh, Edinburgh, UK.

Dr Elisabet M. Rothenberg
Göteborg University, Göteborg, Sweden

Dr Albertine J. Schuit
School of Social and Behavioral Sciences, University of Tilburg, Tilburg, Netherlands.

Dr Sabine Schulz
University of Maastricht, Maastricht, Netherlands

Dr Amy Subar
Epidemiology and Genomics, Division of Cancer Control, NIH, Bethesda, MD,

Dr Minna Tanskanen
University of Jyväskilä, Jyväskilä, Finland

Dr Ricardo Uauy
Institute of Nutrition and Food Technology (INTA), University of Chile, Santiago Chile.

Dr Mauro E. Valencia
Department of Nutrition and Metabolism, Nutrition Coordination, Research Center for Food and Development (CIAD), A.C. Hermosillo, Sonora, Mexico.

Dr Giulio Valenti
NUTRIM, Maastricht University, Maastricht, The Netherlands.
Phillips Research, Eindhoven, The Netherlands.

Dr Rita Van den Berg-Emons
Maastricht University, Maastricht, The Netherlands

Dr Wim G. Van Gemert
Maastricht University, Maastricht, The Netherlands

Dr Erica J. Velthuis-te Wierik
TNO Nutrition and Food Research Institute, Zeist, The Netherlands

Dr Wilhelmine W. Verboeket-van de Venne
Maastricht University, Maastricht, The Netherlands

Dr Jeanine Verbunt
NUTRIM, Maastricht University, Maastricht, The Netherlands.

Dr Jonathan C. K. Wells
Population, Policy and Practice Research and Teaching Department, UCL Great Ormond Street Institute of Child Health, London, UK.

Dr Brian M. Wood

Department of Anthropology, University of California Los Angeles, Los Angeles, USA.
Max Planck Institute for Evolutionary Anthropology, Department of Human Behavior, Ecology, and Culture
