# Supplementary material for: Author Correction: Predictive equation derived from 6,497 doubly labelled water measurements enables the detection of erroneous self-reported energy intake
Source: Nat Food. 2025 Apr 23;6(5):523–4. doi: 10.1038/s43016-025-01175-2 (PMC12116378; doi:10.1038/s43016-025-01175-2)
Supplement: Supplementary file 1 — Original, uncorrected article and Supplementary information. [file 43016_2025_1175_MOESM1_ESM.pdf]

nature food

Supplementary information

Author Correction: Predictive equation derived from 6,497 doubly labelled water measurements enables the detection of erroneous self-reported energy intake

In the format provided by the authors and unedited

Original, uncorrected article

nature food

Article

https://doi.org/10.1038/s43016-024-01089-5

Predictive equation derived from 6,497 doubly labelled water measurements enables the detection of erroneous self-reported energy intake

Received: 8 November 2023

Accepted: 7 November 2024

Published online: 13 January 2025

Check for updates

A list of authors and their affiliations appears at the end of the paper

Nutritional epidemiology aims to link dietary exposures to chronic disease, but the instruments for evaluating dietary intake are inaccurate. One way to identify unreliable data and the sources of errors is to compare estimated intakes with the total energy expenditure (TEE). In this study, we used the International Atomic Energy Agency Doubly Labeled Water Database to derive a predictive equation for TEE using 6,497 measures of TEE in individuals aged 4 to 96 years. The resultant regression equation predicts expected TEE from easily acquired variables, such as body weight, age and sex, with 95% predictive limits that can be used to screen for misreporting by participants in dietary studies. We applied the equation to two large datasets (National Diet and Nutrition Survey and National Health and Nutrition Examination Survey) and found that the level of misreporting was >50%. The macronutrient composition from dietary reports in these studies was systematically biased as the level of misreporting increased, leading to potentially spurious associations between diet components and body mass index.

Diet is a major modifiable factor implicated in many chronic diseases. A persistent problem, however, is accurate quantification of what people eat. Without this information, it is impossible to link nutritional exposures to disease outcomes[1]. The commonest tool for assessing diet is the food frequency questionnaire, which asks individuals to recall frequencies of intake of various foods over protracted periods. Shorter-term instruments used to identify detailed dietary intake require individuals to estimate and record the amount of food that they are eating (for example, food intake diaries) or recall what they ate in the recent past (for example, 24 h recall)[2]. All these methods are prone to 'misreporting' because people cannot accurately estimate the amount of food they are eating, have fallible memories for their intake[3,4] and may, in some cases, deliberately falsify reports[4–7]. In addition, for food intake diaries, people may react during the period of recording by changing their intake[7]. 'Misreporting' also includes a range of other issues, such as how dietary intake reported by participants is converted into energy and nutrients by the investigator, for example, by assuming that all apples are the same size. Moreover, because food intake varies enormously on a day-to-day basis[8,9], individuals may faithfully report what they eat on a given day, but that day may be unrepresentative of what they routinely eat (often called 'under- or overeating')[10]. Making repeated measures using the same instrument on different days may minimize this last problem, but given the variability in daily intake, the number of days that would be required to reduce the variation to a reasonable level is unrealistic for most population survey studies[9,11]. The problems of misreporting and under- or overeating likely occur simultaneously in many situations. Henceforth, for brevity, we will refer to the phenomena of misreporting and under- or overeating as misreporting.

Misreporting has real negative consequences. For example, the failure to recognize these problems led to decades of thinking that people with obesity had very low energy intakes, and hence the positive energy balance leading to their obesity must be a defect in energy

e-mail: yyamada831@gmail.com; aluke@luc.edu; pontzer@gmail.com; Jennifer.rood@pbrc.edu; sagayama.hiroyuki.ka@u.tsukuba.ac.jp; dschoell@nutrisci.wisc.edu; k.westerterp@maastrichtuniversity.nl; wwong@bcm.edu; j.speakman@abdn.ac.uk

expenditure. It later turned out that measured energy expenditures among people with obesity are not low[12]. The problem of misreporting is so ubiquitous and severe that there have been calls for journals to stop publishing studies based on methods that depend on participants estimating their own dietary intake[13]. Yet, such studies continue to proliferate in the literature. This popularity is perhaps because these tools continue to be endorsed by various government bodies, such as the National Cancer Institute (https://epi.grants.cancer.gov/asa24/respondent/validation.html), people have been convinced by arguments that they do have utility[14] and because there are no other feasible, affordable and practical ways to assess total dietary intake.

When the problem of misreporting was first recognized in the late 1980s[15–17], an attempt was made to define cut-off limits by which intake records could be screened for credibility[18]. This was initially done by predicting a person's basal energy expenditure (BEE) using prediction equations based on height, body weight, sex and age. The estimated BEE was then multiplied by 1.35 on the presumption that a daily total energy expenditure (TEE) lower than 1.35 × BEE would be incompatible with survival. This limit is generally referred to as the 'Goldberg cut-off'. However, this approach is susceptible to two major problems: error in the predicted resting metabolic rate and the arbitrary nature of the 1.35 multiplier. Accordingly, the method can only detect and exclude very low reported intakes[19] and many other inaccurate estimates may evade detection. These problems were detailed by Black[9], who modified the cut-off taking into account levels of physical activity and measurement errors in the BEE. This led to the 'modified Goldberg cut-off'. Nevertheless, despite these improvements, the method still relies on the estimated BEE and requires some unverified assumption of the expected physical activity level (PAL).

The doubly labelled water (DLW) technique measures energy expenditure directly from the elimination of isotopes of oxygen and hydrogen introduced into the body in water[20]. The method has an analytical error of about 7% depending on the equation that is used[21]. McCrory et al.[22] introduced a new way to use measurements based on DLW to screen dietary recalls. This method was based on predicting TEE from regression equations based on earlier DLW measurements using age, sex, weight and height as predictors. The TEE estimate used by McCrory et al.[22] employed the equation of Vinken et al.[23]. The standard deviation (SD) of the prediction was then used to define cut-offs (at 1 and 2 SDs) to identify under- and over-reporters of food intake. Although the approach of McCrory et al.[22] has many benefits compared with the use of the Goldberg and modified Goldberg cut-offs, it is hampered by its reliance on equations derived from a relatively small sample of 93 individuals (44 males and 49 females), which included no men between the ages of 28 and 60, and no children or adolescents. Moreover, the cut-off limits of 1 and 2 SDs are also arbitrary.

In this context, we have assembled a database of DLW measurements of healthy individuals[24]. The database includes measurements of over 7,500 individuals of diverse ethnicity aged 8 days to 96 years. Hence, energy demands through the life course have been documented in unrivalled detail[25] and other factors, such as ambient temperature, that may influence TEE have been elucidated[26]. Although we previously published prediction equations for TEE using this database[25], these equations took as their inputs fat-free mass (FFM) and fat mass (FM), measures that are routinely unavailable in dietary surveys. In this study, we derived prediction equations for TEE and confidence limits based on easily measured input parameters using 6,497 available data divided into 5,899 individuals as the analysis set and 598 as the validation set. These equations allow the identification of individuals who may be under- or over-reporting intake in dietary surveys. We demonstrate their use in two publicly available dietary surveys, namely, the National Diet and Nutrition Survey (NDNS)[27] and the National Health and Nutrition Examination Survey (NHANES)[28], which include a total of 18,567 individuals, showing that the level of dietary under-reporting is underestimated by previous tools and that this introduces bias in evaluating dietary composition.

Results

Predictive models

We used two main approaches. The first was classical general linear regression modelling including putatively important factors and their two-way interactions as predictor variables. The variables included were body weight, height, age, age × age (age^2), self-reported ethnicity, sex and elevation above sea level of the measurement site. In the second approach, we used three machine learning models (Random Forest, XGBoost and Support Vector Regression) to derive predictions. These machine learning models did not improve on the classical general linear regression modelling (Supplementary Information and Supplementary Table 1), probably because the predictors in question were linearly related to the output variable. Hence, further treatment was based on only the general linear regression modelling.

The derived significant predictors and their regression coefficients are reported in Table 1. The most significant predictor was the natural logarithm of body weight (ln(BW)). The other primary variables, that is, height, age, age^2, elevation and sex, were all highly significant ($P < 10^{-6}$ in all cases). Females had lower TEE than males. White (non-Hispanic) participants tended to have slightly higher TEE and African participants living outside Africa (AA) slightly lower, and both were highly significant effects ($P < 10^{-9}$). The effects of other groups, however, did not reach significance. The final model explained 69.8% of the variation in ln(TEE).

In this analysis, we were able to derive a predictive equation with each coefficient reduced to four significant figures. The difference between the calculations conducted using this equation and an equation using full precision (10 decimal places) for the coefficients in Table 1 for a random sample of 250 measurements was 0.03%. Reducing the significant figures to three increased the discrepancy by a factor of ten (0.4%).

$$\begin{aligned}\ln(\mathrm{TEE}) = \\ &-0.2127 + 0.4167 \times \ln(\mathrm{BW}) + 0.006565 \times \mathrm{Height} \\ &-0.02054 \times \mathrm{Age} + 0.0003308\mathrm{Age}^2 - 0.000001852 \\ &\times \mathrm{Age}^3 + 0.09126 \times \ln(\mathrm{Elevation}) - 0.04092 \times \mathrm{Sex} \\ &+0.01940 \times \mathrm{A} - 0.03899 \times \mathrm{AA} + 0.006238 \times \mathrm{AS} \\ &+0.02626 \times \mathrm{W} - 0.0155 \times \mathrm{H} + 0.003589 \times \mathrm{NA} \\ &-0.0006759 \times \mathrm{Height} \times \ln(\mathrm{Elevation}) + 0.002018 \\ &\times \mathrm{Age} \times \ln(\mathrm{Elevation}) - 0.00002262 \times \mathrm{Age}^2 \\ &\times \ln(\mathrm{Elevation}) - 0.006947 \times \mathrm{Sex} \times \ln(\mathrm{Elevation})\end{aligned}$$

Here, TEE is in megajoules per day, BW is in kilograms, height is in centimetres, age is in years, sex is coded −1 for males and +1 for females, and the elevation of the measurement location is in metres. For the self-reported ethnicity codes, for African, A was 1 and 0 otherwise, for African individuals living outside Africa, AA was 1 and 0 otherwise, for Asian, AS was 1 and 0 otherwise, for white, W was 1 and 0 otherwise, for Hispanic, H was 1 and 0 otherwise, for not available, NA was 1 and 0 otherwise. Mixed race individuals were coded as NA (see Methods). Two worked examples for the calculation of TEE for two different individuals are provided in Supplementary Table 7.

The residuals of the prediction were well distributed with respect to the major predictors, suggesting that the prediction was not biased (Supplementary Fig. 1). In addition, there was no significant relationship between the residual of the prediction and weight change during the measurement period ($n = 3{,}088$ with reported weight change, $F = 0.19$ and $P = 0.665$; Supplementary Fig. 2a), suggesting that the

Table 1 | Significant terms in the general linear model analysis (10 decimal places) predicting TEE

Term	Coefficient	SE coefficient	*T* value	*P* value
Constant	−0.21723930921	0.0757	−2.87	0.0041
ln[BW (kg)]	0.41666419569	0.00958	43.51	$<10^{-9}$
Height (cm)	0.00656496388	0.000618	10.62	$<10^{-9}$
Age (yr)	−0.02054339322	0.00218	−9.41	$<10^{-9}$
Age^2 (yr^2)	0.00033079019	0.000037	9.06	$<10^{-9}$
ln[Elevation (m)]	0.09126350903	0.0186	4.89	0.000001
Sex	−0.04091711710	0.00769	−5.32	0.00000011
Ethnicity[a]				
A	0.01939639976	0.00749	2.59	0.0096
AA	−0.03899332615	0.00544	−7.17	$<10^{-9}$
AS	0.00623768257	0.00808	0.77	0.44
W	0.02625775059	0.00397	6.62	$<10^{-9}$
H	−0.01554772302	0.00982	−1.58	0.11
NA	0.00358921276	0.00636	0.56	0.57
Height×ln[Elevation (m)] (cm)	−0.00067594646	0.000136	−4.98	0.00000066
Age×Age^2 (yr^3)	−0.00000185178	0.000000	−8.93	$<10^{-9}$
Age×ln[Elevation (m)] (yr)	0.00201815477	0.000383	5.28	0.00000014
Age^2×ln[Elevation (m)] (yr^2)	−0.00002262281	0.000004	−5.89	0.0000000041
ln[Elevation (m)]×Sex	−0.00694699228	0.00179	−3.87	0.00011

[a]A, African; AA, African living outside Africa; AS, Asian; W, White; H, Hispanic; NA, not available. SE, standard deviation.

energy expenditure from this predictive model is a good proxy for intake in individuals that are not attempting to lose or gain weight or suffering loss of appetite due to illness. That is because the metabolic rate generally declines when individuals are engaged in deliberate weight loss, and the opposite happens during overfeeding. Hence, if individuals were not in energy balance, we would expect a positive relationship between weight change and residual energy expenditure. Ninety-five per cent predictive intervals (95% PI) are the range of values that are 95% likely to contain the true value for a single new observation based on specific values of the predictor variables. The predictive interval depends on the *T*-critical value for the given confidence, the estimated mean and the standard error of the response variable, the sum of squares and the specific and mean values of the predictor variables, and the total sample size on which the prediction equation is based. For all of the test samples, we used standard statistical software (Minitab v19, https://www.minitab.com) to calculate the upper and lower predictive intervals and then defined two additional equations to identify the 95% PI around the predictions.

This gave

$$\text{Lower 95\% PI} = (p\text{TEE} \times 0.7466) - 1.5405$$

$$\text{Upper 95\% PI} = (p\text{TEE} \times 1.3395) + 2.7668$$

where *p*TEE is the predicted mean TEE (MJ d^{-1}). This interval provides an objective evaluation of the confidence that can be placed in any given prediction using the derived regression equation. Using this predictive interval to screen observations is a superior approach to previous attempts to screen dietary reports, which were all based on arbitrary cut-off points.

For the 598 individuals in the validation set, we derived the predicted TEE and the upper and lower 95% PI for the mean estimates using the equations derived above (Supplementary Fig. 2). We then counted the number of actual measurements of TEE in the validation set that fell outside the predictive interval for TEE (Supplementary Fig. 2b). In total, from 598 measurements in the validation set, 14 fell below the lower predictive interval (2.3%) and 20 were above the upper predicted limit (3.3%). The validation dataset confirmed that 94.6% of independent TEE measurements were within these 95% predictive limits (Supplementary Fig. 2b). We then explored whether the equations could produce credible predictions for groups that were not included in the original derivation but whose data were available in the database, specifically 246 athletes and individuals engaged in unusual levels of physical activity and 176 females during reproduction. The predictions significantly underestimated the observed expenditures of all these groups (Supplementary Information, Supplementary Fig. 3 and Supplementary Table 6). Therefore, the prediction equation derived here cannot be used for these populations.

Application to exemplary survey data

Demographic statistics for the individuals used in the comparison are presented in Supplementary Table 2. In total, there were 12,694 records available in NDNS and 5,873 in NHANES. On average, the individuals in NHANES were around 6–10 years older than those in NDNS. Twenty-five per cent of the sample in NHANES self-reported as African living outside Africa and 22% as Hispanic. In contrast, 94% of the participants in NDNS self-reported as white. We compared the net energy intake with the predicted TEE from the above equation. Using the predictive equations developed above, the number and percentage of individuals that fell outside the predicted limits (both over and under) and within the predicted limits are shown in Table 2, stratified by data source, age (adults versus children) and sex.

For adults in NHANES, approximately 43.7% of dietary reports were within the predictive interval (44.9% for males and 42.7% for females). For children, the percentage within range was considerably higher (by 13–14%) than for adults. A similar pattern was noted for the NDNS data. For adults, 36.6% of males and 38.1% of females were within the prediction interval. The percentage of children in range was about 20% higher than for adults. This means that a large percentage of data fell below the lower predictive interval due to either undereating

https://doi.org/10.1038/s43016-024-01089-5

Table 2 | Summary of observations inside and outside the tolerance limits in the NDNS and NHANES datasets

	Number underestimated	Percentage of total	Number within range	Percentage of total	Number overestimated	Percentage of total	Total
**NDNS**							
Male children	1,078	43.02	1,416	56.50	12	0.48	2,506
Female children	942	39.71	1,394	58.77	36	1.52	2,372
Male adults	2,068	62.8	1,204	36.56	21	0.64	3,293
Female adults	2,783	61.53	1,721	38.05	19	0.42	4,523
Male all	3,146	54.25	2,620	45.18	33	0.57	5,799
Female all	3,725	54.02	3,115	45.18	55	0.80	6,895
**NHANES**							
Male children	251	35.35	408	57.46	51	7.18	710
Female children	248	32.89	426	56.5	80	10.61	754
Male adults	1,063	50.40	947	44.90	99	4.69	2,109
Female adults	1,233	53.61	981	42.65	86	3.74	2,300
Male all	1,314	46.61	1,355	48.07	150	5.32	2,819
Female all	1,481	48.49	1,407	46.07	166	5.44	3,054

The data show the numbers and percentages of participants that fall inside and outside the tolerance limits in the NDNS dataset (years 1–11) and the NHANES dataset (2017–2018).

or misreporting. For NHANES, the figures for adults were 50.4% in males and 53.6% in females. For NDNS, the overall values for adults were 62.8% in males and 61.5% in females. In both surveys, children were less likely to under-report/undereat by 15–22%. We compared the detection of under-reporting using our equation with the previous models proposed by Goldberg et al.[18], Black[9] and McCrory et al.[22] (Supplementary Table 3). On average, the Goldberg and Black cut-offs indicated far less under-reporting (25–40% of individuals) than is indicated here. The level of under-reporting identified using the McCrory et al.[22] equation depended very heavily on whether 1 or 2 SDs were used as the cut-off. With 1 SD, the level of under-reporting was greater than we predicted (60–75%), but it was much less using 2 SDs (22–37%).

Effects of age and body mass index on under-reporting

We plotted the difference between the survey estimate of daily energy intake and the predicted TEE as a function of age and body mass index (BMI) for both the NDNS and NHANES datasets (Fig. 1). In adults, the extent of under-reporting was almost independent of age in both datasets, although there was a slight improvement with age in the NDNS dataset ($P < 0.001$). The average discrepancy in the NDNS was 3.5 MJ for both females and males. In NHANES, the average discrepancy for males was 1.8 MJ and for females it was 2.8 MJ. In both the NHANES and NDNS surveys, the data for the very young, whose surveys were generally completed by their care providers, were at or slightly above the expected intakes. There was a strong deterioration in the number of plausible estimates through childhood as the children started to complete their own surveys, until, by age 16, the discrepancies matched the adult levels (Fig. 1a). The deficit between reported intake and predicted expenditure was strongly negatively correlated with individual BMI (Fig. 1b). In both surveys, there was no discrepancy between what adults and children with a BMI of around 15–20 kg m^{-2} reported eating and their predicted expenditure. However, the discrepancy got larger as the BMI increased in both adults and children. The effect in children was greater than in adults. Hence, in NDNS, a child with a BMI of 40 kg m^{-2} had a discrepancy of 9 MJ d^{-1}, while an adult with a BMI of 40 kg m^{-2} had a discrepancy on average of only 5 MJ d^{-1}. In NHANES, for a BMI of 40 kg m^{-2}, the discrepancies were 8 MJ d^{-1} for children and 4 MJ d^{-1} for adults.

Under-reporting in relation to macronutrient intake

Next, we explored the relationship between the discrepancy in energy intake and the proportional macronutrient composition (percentage energy) of the reported diet (Table 3). If there was no bias in the under-reporting, then we would expect no differences in the coefficients with respect to the different macronutrients. Contrasting this prediction in the data that were not screened, there was a strong relationship between the reported percentage of energy as protein in the diet and the absolute size of the energy discrepancy (Fig. 2). As the level of protein in the diet increased, the discrepancy became more negative. For each 1.0% increase in reported protein energy, the difference between reported energy intake and actual intake decreased by around 200 kJ d^{-1} in both NDNS and NHANES (Table 3). Note that as most data fall below the line of equality, this negative relationship means that as the self-reported percentage of protein in the diet increased, the discrepancy between the self-reported total energy intake and the predicted total energy expenditure got larger (Fig. 2). In contrast, as the percentage of fat energy in the diet increased, the discrepancy between the reported and predicted intake became more positive and the discrepancy got smaller (Fig. 2). The effect was smaller than the impact of protein and was different between surveys. The effect in NDNS was approximately twice as large as that in NHANES. In NDNS, there was no significant effect of the percentage of carbohydrate energy in the diet on the discrepancy, but in NHANES, carbohydrates had a similar direction of effect as protein, but the effect size was about a tenth as large (Fig. 2 and Table 3). These differences indicate that the assumption in dietary surveys that diet composition is independent of the extent of misreporting is likely to be false. Individuals who under-reported their total energy intake also reported a greater percentage of protein energy and a reduced percentage of fat in their diets (Fig. 2). These effects are unlikely to be limited to macronutrients as the total energy and macronutrient composition are derived from the self-reported list of food items consumed. The bias in macronutrient reporting found in this study strongly suggests corresponding recall bias in the types of food recalled and thus micronutrient intakes as well. The magnitude of this effect may well depend on the food and nutrient examined.

Screening the data using the tool presented here to remove those outside the predictive interval (that is, under- and over-reporters) massively attenuated these bias effects (Fig. 2 and Table 3). However, this course of action necessitates the removal of a large percentage of the collected dietary records and this is wasteful of the effort to collect such data. There are several possible alternative approaches. One potential method is to conduct the analysis including or excluding the data outside the predictive interval. If there are no biases, then

 https://doi.org/10.1038/s43016-024-01089-5

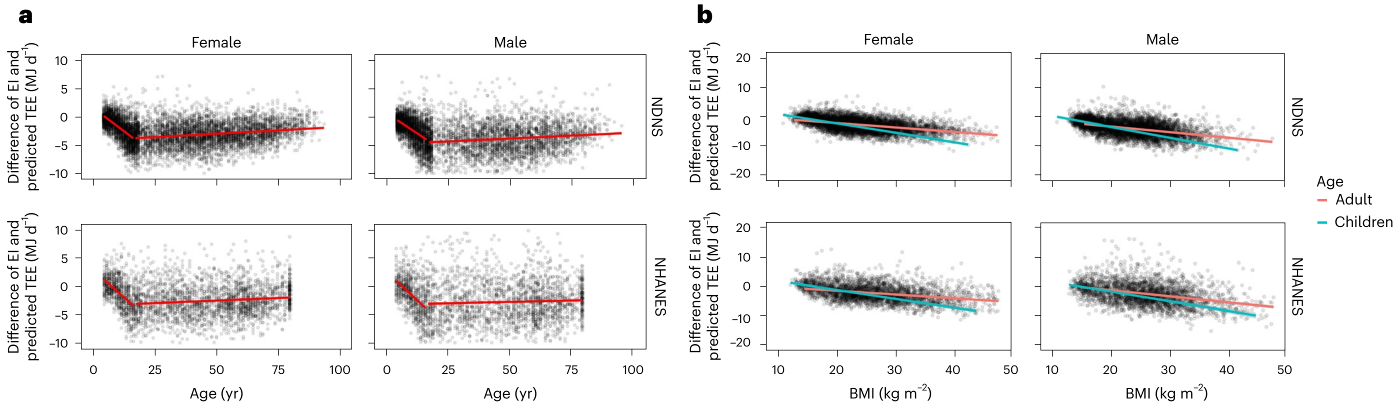

Fig. 1 | Misreporting in relation to age, BMI and sex. a, Comparison of the difference between predicted TEE and self-reported energy intake (EI) in the NDNS (n = 12,694) and NHANES (n = 5,873) datasets in relation to age for children (≤16 yr) and adults (>16 yr). **b**, Comparison of the difference between predicted TEE and self-reported energy intake in the same datasets in relation to BMI for children (≤16 yr) and adults (>16 yr). Negative values show observations lower than prediction and positive values show prediction higher than observation.

the outcomes should be the same and in such a case reverting to the full dataset would be appropriate. Another alternative is to model the factors that influence the differences between the data identified as implausible and attempt some form of correction of the problematical dietary records. Whatever the adopted approach, we suggest that by using the tool that we provide here, nutritional epidemiologists may enhance the quality of their work and have greater confidence in their conclusions.

As there is a systematic trend between macronutrient intake and the extent of under-reporting and because under-reporting is related to BMI, there was a strong positive relationship between the reported dietary intakes of protein and BMI in both surveys (Fig. 3 and Table 4). In contrast, there was a strong negative effect for carbohydrate intake (Fig. 3 and Table 4), while the relationship of fat intake to BMI differed between the surveys, being positive in NHANES and negative in NDNS. The strengths and gradients of these effects were significantly impacted by restricting the analysis to only those data within the acceptable range. The gradient and R^2 values of the relationship between BMI and protein were both strongly reduced (Fig. 3 and Table 4), while the negative gradient for the relationship between BMI and carbohydrates became more negative and the R^2 value approximately doubled. Higher carbohydrate intake was therefore strongly associated with a lower BMI. The relationship for fat content also became stronger (R^2 increased) and the gradients, previously showing different trends for the two surveys, were both positive. Higher reported fat and protein intakes were both strongly associated with a higher BMI. All the relationships were highly significant ($P < 10^{-4}$, Table 4).

Discussion

Impact of repeated recalls on survey validity

If the problem with misreported intakes reflects undereating rather than under-reporting, then making repeated surveys should alleviate the issue, unless the undereating is a direct response to the survey instrument. This could be an issue for food diaries, but should not be an issue with 24 h recall. However, if participants developed reporting fatigue, one might anticipate that the accuracy of reporting would decline as the number of surveys was increased. In NDNS, some participants completed four surveys, while in NHANES, some participants completed two. The number of individuals who fell within the expected range was independent of the day of survey in NDNS (Supplementary Table 4a). This suggested that there was no survey fatigue across the 4 days. When the average intakes were taken across multiple days, this did not improve the percentage that fell within the predicted range (Supplementary Table 4b). This indicates that the general problem of

Table 3 | Relationships between the discrepancy of intake to expenditure and self-reported dietary macronutrient composition

**NDNS (full data)**			
**Term**	**Coefficient**	**SE coefficient**	***P* value**
Constant	−1,360.8	366.3	0.0002
Percentage carbohydrate	0.64	3.86	0.87
Percentage protein	−207.3	6.42	<0.0001
Percentage fat	53.40	4.49	<0.0001
R^2 (%)			12.24
NDNS (screened)			
Term	Coefficient	SE coefficient	*P* value
Constant	−1,713	281	<0.0001
Percentage carbohydrate	16.6	2.92	< 0.0001
Percentage protein	−59.6	6.02	<0.0001
Percentage fat	26.43	3.59	<0.0001
R^2 (%)			3.84
NHANES (full data)			
Term	Coefficient	SE coefficient	*P* value
Constant	1,025.15	936.0	0.27
Percentage carbohydrate	−20.94	9.61	0.03
Percentage protein	−207.65	13.77	<0.0001
Percentage fat	25.27	10.47	0.02
R^2 (%)			5.85
NHANES (screened)			
Term	Coefficient	SE coefficient	*P* value
Constant	−447	656	0.496
Percentage carbohydrate	3.48	6.73	0.605
Percentage protein	−40.7	10.7	<0.0001
Percentage fat	14.3	7.4	0.053
R^2 (%)			0.77

Multiple regression analysis of the discrepancy between intake and predicted expenditure and the self-reported macronutrient composition of the diet in the NDNS and NHANES surveys. In both cases, 'full data' refers to the analysis of the whole dataset and 'screened' relates to the analysis of the screened data.

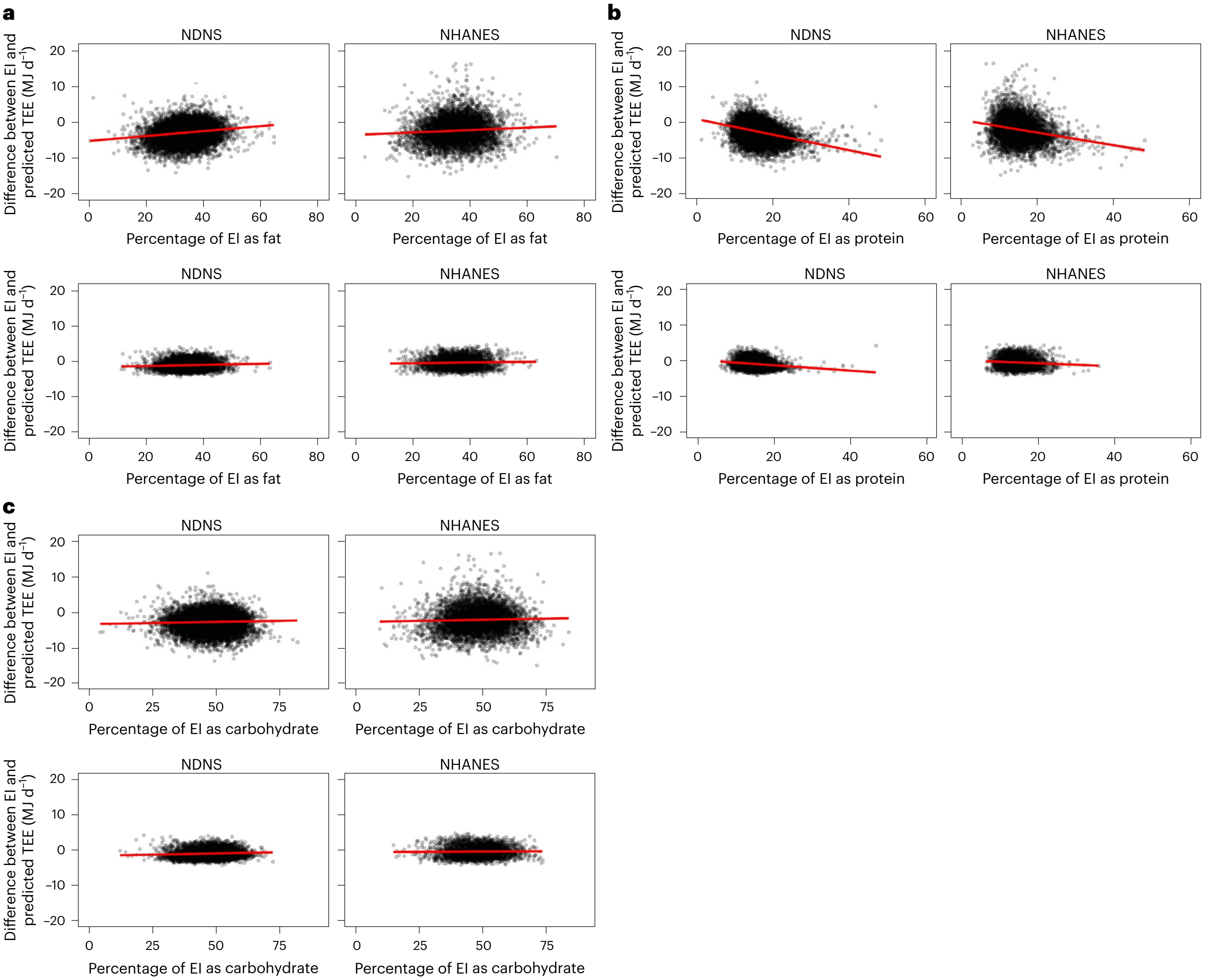

Fig. 2 | Misreporting and macronutrient intake. a–c, The discrepancy between the predicted TEE and the reported energy intake in the NHANES and NDNS surveys plotted against the self-reported intakes of fat (**a**), protein (**b**) and carbohydrates (**c**) as a percentage of the total energy. For each macronutrient, the top two plots show data from the whole sample (full data) and the bottom two plots show the data from the sample screened to include only those individuals within the predictive interval of the equation (screened). Significant effects in the whole sample were severely attenuated in the screened sample (see Table 3 for regression details).

misreporting is not undereating but under-reporting, and that such under-reporting was consistent across days. The consistent magnitude of misreporting suggests that there is little benefit of completing multiple surveys as a mechanism to eliminate misreporting. Similar patterns were found for the NHANES analysis, where the percentage of individuals in the expected range was not different between the two surveys and accuracy was not improved by taking the average (Supplementary Table 5).

The predictive equation based on general linear modelling explained >69% of the variation in TEE. This is less than was achieved with an equation based on fat-free mass, fat mass and age derived from the same dataset[25], which explained 83% of the variation (but in a sample restricted only to adults). The significant effects of additional variables beyond body weight, such as height, sex and self-reported ethnicity, therefore likely exert their effects because these traits also impact FFM as a component of body weight. For example, females of a given height and weight tend to have greater fat mass and lower FFM than males. Thus, when body weight rather than FFM is used as a predictor, sex also enters as a significant term; conversely, when FFM is used as a predictor, sex is no longer significant[24]. The effects of elevation were unanticipated and their numerous interactions with other variables suggest that this may also be related to trends in FFM with elevation. On average, it gets colder as the elevation increases. However, it is unlikely that the elevation effect is due to declining ambient temperature, as in a subset of the same data (restricted to the USA), we found no effect of ambient temperature on TEE[26]. Independent of body composition, it is established that elevation affects BEE, which is a major component of TEE[29]. The age effect included squared and cubed terms, also consistent with previous work suggesting nonlinear impacts of age on metabolic rate[25,30].

If FFM and FM explain more of the variation in TEE, then a valid question is why not use that equation on which to base the screening? The problem with such an approach, however, is the accuracy of the estimates of FFM and FM. In the equations derived previously, the percentage FFM and FM came from isotope dilution estimates of body water, which derive from the DLW method. Performing isotope dilution

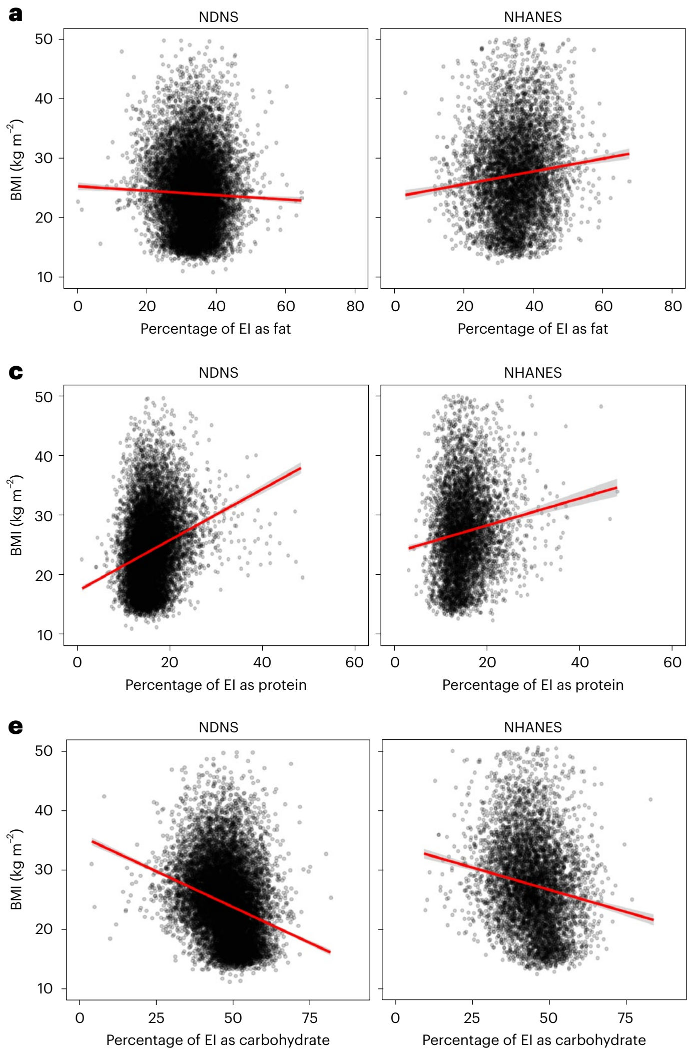

Fig. 3 | Relationships between the reported dietary intakes of macronutrients and BMI. a–f, Relationships between BMI and the intakes of fat (**a**,**b**), protein (**c**,**d**) and carbohydrate (**e**,**f**) for the NHANES and NDNS surveys. Panels **a**, **c** and **e** show the data for the whole sample and panels **b**, **d** and **f** show the data for those individuals whose total energy intake was within the predictive interval (that is, excluding under- and over-reporters).

on all survey participants in large surveys would be challenging and costly. Alternative approaches to measuring FFM in survey settings, however, are less accurate. Thus, the extra predictability of TEE afforded by having estimates of FFM and FM is negated by the reduced accuracy of cheap FFM and FM assessments. A second question is how do the equations take into account different levels of physical activity? The modified Goldberg approach accounts for this by using different levels of PAL (the ratio of TEE to BEE). The main problem with this is equating the PAL level to a level of physical activity[31] and the inaccuracies involved in people self-reporting how active they are. In the current approach, we included a large sample of individuals who have a diversity of PAL levels that make up the total TEE. By predicting TEE directly, we automatically account for the diverse effects that other factors may have on PAL and hence TEE, such as age, sex and ethnicity. The 95% prediction limits therefore cover the vast majority of individuals. The exceptions are groups who have particularly active lifestyles. We showed that the equations significantly underestimate the expenditure of such groups (Supplementary Fig. 3). In addition, the equations significantly underestimated TEE in pregnant and lactating females.

Detection of under- or over-reporting and under- or overeating

There was very little change in the level of undereating/under-reporting with age. In contrast, age has previously been identified as a strong factor for under-reporting energy intake[32–34]. In a previous study, 36% of women and 34% men aged 40–69 years underestimated energy intake[35]. Similarly, in a different study, among women and men ($n = 28$) aged between 35 and 67 years, the discrepancy was 19% (ref. 36). There was no increase in the level of under-reporting in individuals who were aged >70 years, where one might anticipate that memory functions might impair recall fidelity. In contrast, for young children, where intake diaries are generally completed by an adult, the agreement between expectation from the equation and the estimates from the survey report was much better.

It is often claimed that instruments in dietary survey work were designed to assess the types of food being consumed and not the total energy intake. Hence, reported total energy intake could be incorrect, but that does not necessarily mean that the percentage macronutrient compositions are erroneous because the error may be unbiased. If so, it would mean that dietary survey work might not be as flawed as is often claimed[13]. If misreporting was unbiased, then the discrepancy between the survey intake and the DLW prediction would be unrelated to the macronutrient composition of the reported diet. In other words, each macronutrient would have the same relationship to the level of misreporting. This was not the case. The level of reporting was strongly related to the reported protein intake, with lesser and opposite effects for fat. Carbohydrate had lower and contrasting effects

https://doi.org/10.1038/s43016-024-01089-5

Table 4 | Relationships between macronutrient intake and BMI in both datasets

Macronutrient	Survey	Whole data			Within 95% PI		
		Gradient	R^2	*P*	Gradient	R^2	*P*
Percentage fat	NHANES	+0.118	0.0109	$<10^{-15}$	+0.189	0.027	$<10^{-15}$
	NDNS	−0.0376	0.0011	0.000099	+0.0525	0.002	0.00016
Percentage carbohydrate	NHANES	−0.1498	0.0276	$<10^{-15}$	−0.2158	0.053	$<10^{-15}$
	NDNS	−0.2306	0.0709	$<10^{-15}$	−0.3224	0.156	$<10^{-15}$
Percentage protein	NHANES	+0.2309	0.0142	$<10^{-15}$	+0.1853	0.007	0.0000072
	NDNS	+0.4227	0.0578	$<10^{-15}$	+0.2654	0.018	$<10^{-15}$

Multiple regression analysis of macronutrient intake and BMI in the NHANES and NDNS datasets using the whole data and only the observations where the total dietary intake was within the predictive interval of the regression model. Gradient represents the change in BMI for each 1% change in macronutrient intake.

across surveys. When people under-reported their intake, they tended to also report an elevated percentage of protein intake and a lower percentage of fat intake. The relationship between misreporting and macronutrient composition is consistent with previous work showing that under-reporters of total intake also report consuming a greater percentage of protein[37–43]. In these previous studies, only Cook et al.[37], Bel-Serrat et al.[42] and Previdelli et al.[43] observed a contrasting effect for fat consistent with the magnitude of the different fat effects between surveys observed here[44]. Because under-reporting is also strongly linked to BMI (Fig. 1), there is enormous potential to misinterpret associations between dietary survey reports of macronutrient intake and BMI (Fig. 2). BMI-related biases in reporting could affect other analyses as well, for example, the relationship between particular food types and markers of inflammation. We show here that using our tool to identify misreporting individuals, the associations between dietary self-reported macronutrient intake and BMI were significantly modified, indicating the utility of the tool.

Limitations

We used estimates of TEE derived from the DLW method to infer food energy intake. There are several assumptions in this procedure. Converting CO_2 production into energy expenditure depends on knowledge of the respiratory quotient (RQ). In general, the RQ was not known in the studies submitted to the database and an assumed value of 0.83 was used. Deviations from that value due, for example, to having a diet particularly rich in fat or carbohydrates adds error into the estimated TEE. That might then complicate comparing the extent of misreporting with dietary composition. For example, if an individual had an RQ of 0.78, reflecting high fat intake, and we assumed an RQ of 0.83, then we would overestimate TEE and make under-reporting more likely to be detected. However, the difference in TEE in this instance would only be 4.5%, and hence this would have only a marginal impact on the detection of misreporting in relation to fat intake. In addition, converting energy expenditure into an estimate of food intake assumes that the individuals are in energy balance over the time course of the measurement. We consider that the individuals in the sample used to derive the equation were likely to be in energy balance because the residual TEE values were not related to weight change over the interval of the measurement. This is not necessarily the case for individuals involved in dietary surveys and one should always be cautious that deviations from the predictions are not due to misreporting, but because the person was under- or overeating. Although we had a large sample of TEE data, the predictive model explained only 69% of the variation in TEE and the resultant absolute error in the predicted values of the test set averaged 11.2%. Because we used the 95% PI around the average to define implausible records, then by definition 5% of such records will be erroneously identified and in fact be valid reports. In the future, this prediction may be improved by integrating independent measures of physical activity, for example, by accelerometry, into the model. However, the utility of this extra information in terms of detecting erroneous food intake reports in dietary survey work may be limited because few such surveys have objective measures of physical activity collected by, for example, accelerometry.

Implications and future directions

Accurately measuring what people eat is essential for understanding the consequences of components of food intake for health. It also contributes to our understanding of many other areas, including food security and quantifying food waste. The main tools that we currently use to do this were developed more than 50 years ago, they depend on self-report and are widely acknowledged to provide inaccurate information. Tools to identify misreported data already exist. In this study, we developed an enhanced approach to identify potentially erroneous and implausible reports. The tool is not perfect and it will itself misidentify about 5% of reports as wrong when they are in fact correct, but it improves on previous approaches to identify problematical data. Applying the tool to two large surveys suggested that more than 50% of the dietary reports had implausible energy intakes and probably therefore erroneous intake of macro- and micronutrients. Ultimately, the main benefit of this tool is that it may highlight the true level of dietary misreporting when using existing methods and drive us towards innovating radical approaches that do not rely so much (or at all) on self-report.

Methods

This is a retrospective analysis of cross-sectional data. Data collection started before establishment of the clinical trials registry. The goals of this analysis were pre-registered on the International Atomic Energy Agency (IAEA) DLW Database site in 2020. The original data on which it is based were subject to ethical review at diverse institutions.

Developing the prediction algorithm

The predictive algorithm was derived from an analysis of measurements submitted to the IAEA DLW Database (version 3.6; dlwdatabase.org). This included data derived from DLW studies in 32 countries with 7,646 male and female participants, compiled from 128 different published and unpublished studies. The measurements relate to individuals who were not engaged in dietary or exercise interventions. The component studies have generally screened out people who have specific diseases, such as type 2 diabetes or cancer, in their recruitment processes. Therefore, these groups are not represented in the data and may have different levels of energy expenditure and food intake from those predicted here. In addition, we further eliminated data relating to individuals engaged in unusual levels of physical activity (for example, participants in the Race Across America[45] or individuals climbing Mount Everest[46]), measurements of amateur or professional athletes (for example, professional footballers[47] and jockeys[48]) and females who were pregnant or lactating. We did not eliminate measurements of hunter–gatherer[49] and subsistence agriculture populations[50] as evidence suggests that these do not differ from westernized

https://doi.org/10.1038/s43016-024-01089-5

populations in their energy expenditures, once normalized for body weight. However, such measurements comprised less than 1% of the total and their inclusion or exclusion does not materially alter the predictive equations. In total, we had measurements for 7,441 individuals that met all the inclusion criteria.

The data in the database were all recalculated using a common equation that was shown in validation against chamber calorimetry to provide the most accurate and precise measure of CO_2 production[21]. These estimates were converted to TEE using the modified Weir equation[51] with either a known food quotient, a measured respiratory quotient derived from 24 h chamber calorimetry or, in the absence of other information, an assumed RQ of 0.85. An initial analysis suggested that deriving a common equation that covered all age classes had a high level of residual error. The structure of the residuals showed that most error was incurred among the youngest participants. We therefore restricted the final analysis to individuals aged ≥4 years. In total, for this age group, we had 6,497 measurements available. We assigned random numbers between 0 and 10,000 to the measurements and then sorted them in order of increasing random number. We then selected the first 90% of measures (n = 5,899) as the analysis set and retained the remainder as a validation set (n = 598). These data were derived principally from the USA and Western Europe (87.8%), measured mostly since 2000, with lesser contributions from other countries. They are dominated by white (56.5%) and African American (15%) ethnic groups, with lesser contributions by Hispanic, African and Asian ethnic groups (all ethnicities by self-report). We included the elevation of the study location, but did not include ambient temperature during the measurement period because a previous analysis has shown that this is not a significant predictor, at least for data from the USA[26]. Moreover, this is not generally available for survey work. We did not use date of measurement despite recently showing that TEE has declined over time in the USA and Europe in adults[52] because the current data include children between the ages of 4 and 16 as well as data from additional countries where this relationship to time does not necessarily apply. Moreover, we cannot be sure that this trend will continue into the future.

We combined the TEE measurements with additional information that can be routinely measured in survey work without the need for complex equipment. These extra variables (with the measurement units) were body mass (kg), height without shoes (cm), self-identified sex (m/f), age (yr) and self-reported ethnicity. Ethnicity included African, African living outside Afirca, Asian, white, Hispanic and not-available (10.4%). A small number of individuals identified as mixed race or 'other' (2.9%) and these were all coded as 'not available' as there were insufficient data to include different combinations separately. We are aware of the discussions regarding the inclusion of ethnicity into analyses of this type and of the history of their misuse in medicine and biology. By including self-reported ethnicity, we do not intend to imply that there is any fundamental physiological or genetic basis to these differences, or that any particular group has 'superior metabolism' compared to others. We emphasize that these are self-declared ethnicities and not attributed. If self-declared ethnicity was unavailable in a particular survey or if there were objections for whatever reason to the use of ethnicity as part of the prediction model, then the default was to use 'not available', which has a coefficient approximating to 0.

Because the relationship between body mass and TEE follows a power law[25], we log-converted TEE and body weight before analysis. We log-transformed other variables such as elevation because they were not normally distributed. Moreover, as there is a curvilinear relationship of the normalized TEE with age, we included both age and age^2 as predictors. We then fitted a generalized linear model to the data using the statistical program Minitab (v19), including all of the primary variables and all of the interaction terms (up to three way). We refined the model by retrospectively deleting non-significant terms, starting with the three-way interactions, and then non-significant two-way interactions. Seventy sets of data were eliminated because of incomplete predictor data (all missing the elevation of the measurement site). We plotted the residual variation against the original predictors to assess whether there was any bias in the predictions (Supplementary Fig. 1). This suggested that the predictors were not biased. Predicted TEE might not be a good estimator of energy intake if individuals are changing weight during the measurement period. That is because when individuals are gaining weight they may be consuming more than they expend, and vice versa when they are losing weight. However, there was also no significant relationship between weight change during the measurement period and the TEE (Supplementary Fig. 2a), suggesting that this did not compromise the predictions. This could be because the majority of weight difference over the 2-week measurement period is not stored energy (for example, most of it is water and perhaps differences in gut fill) and that the remaining energy storage is relatively small compared with total expenditure over a 2-week interval. We did not have information on weight change over longer periods to evaluate whether that influenced the measurements. A recent study found that eliminating individuals who had greater than 5% weight change over the 6 months preceding the TEE measure attenuated the relationship between TEE and all-cause mortality[53]. Childhood growth might also affect the assumption that TEE is equal to energy intake. If we take the extreme example of a rapidly growing adolescent gaining 10 kg per year, that would be equivalent to 0.38 kg over a typical 14-day DLW measurement. If we assume that this mass comprised 65% water, 20% lean tissue and 15% fat, then the extra energy intake above expenditure to deposit this tissue would be about 0.3 MJ, or about 3% of energy expenditure. The direction of this discrepancy would push participants towards over-reporting.

Validation

We compared the predicted TEE with the observed TEE for the randomly selected 598 data in the validation dataset (Supplementary Fig. 2a). There was a strong correspondence between the observations and the predictions (R^2 = 0.67), and 94.6% of the observations were within the 95% PI of the corresponding predictions. The average absolute deviation between the prediction and observation in this validation set was 11.2%. In addition, we explored whether the predictions from the equation might be valid for other groups not involved in the derivation of the equations, specifically athletes, individuals engaged in unusual activity and reproductive females. In all cases, the observed expenditures of these special groups exceeded the predictions. The average discrepancy across all of the athletes was 8.9 MJ d^{-1} (SD = 1.59) and across all of the reproductive females was 8.04 MJ d^{-1} (SD = 1.50). A more detailed breakdown is provided in Supplementary Table 6. This confirms that the prediction equation cannot be used in these unusual cases.

Sensitivity analysis

Survey work may not always have all the data available on which to make a prediction. We considered the impact of not having the elevation of the person's location and not having the person's self-reported ethnicity. For the sensitivity to elevation effects, we compared the predicted TEE in the validation set with the predicted TEE using a 'dummy' elevation of 100 m. The absolute error in the predicted TEE by using the dummy elevation in the validation dataset was 2.3%. We also explored the impact of not knowing the ethnicity on the predicted total energy expenditure (TEE). The ethnic category 'not available' was used as a standard to calculate the impact of knowing or not a person's ethnicity. The change in predicted TEE by knowing the person's ethnicity compared with 'not available' was 2.29% for white, −4.17% for African living in Africa, 1.59% for African living outside Africa, 0.27% for Asian, −1.9% for Hispanic and −0.36% for 'other'. In general, these errors were small relative to the predictive interval, but clearly having a complete predictor dataset provides a better prediction than incomplete data.

Machine learning approaches

We used three different machine learning approaches to analyse the data using the same predictor variables: Random Forest, XGBoost and Support Vector Regression. Random Forest is a model that uses multiple trees to train and predict samples. It builds multiple unrelated decision trees by randomly drawing samples and features to obtain predictions in parallel. Each decision tree yields a prediction from the samples and features drawn, and the regression prediction for the whole forest is obtained by combining the results of all the trees and taking the average. Features are randomly selected as the subset of features to be selected when building the tree. Random forests are resistant to overfitting and do not require feature selection. However, as Random Forest does not give continuous output values, it may not be as effective in solving regression problems as it is for solving classification problems. Moreover, if the noise level in the data is high, the performance of Random Forest may decay. XGBoost is a machine learning library that focuses on gradient-boosting algorithms. It was created in 2014 and has attracted much attention for its excellent learning results and efficient training speed. The XGBoost regression that we used is an optimization algorithm for Gradient Boosting Decision Tree (GBDT) regression. GBDT works by training a tree using the training set and the true values, then using this tree to predict the training set and obtain the predicted values for each sample. Hence, we obtained the residual, which was the difference between the true values and prediction. We can then train a second tree, at which point the true value is no longer used, but the residual is used as the standard answer. Once the two trees are trained, the residuals can be obtained again for each sample, then a third tree is further trained, and so on. In short, the GBDT will learn the residual based on previously built trees in each step. We can artificially specify the total number of trees or monitor certain parameters to stop the training procedure. XGBoost improves the GBDT by adding regularization, parallel processing and built-in cross-validation. XGBoost can automatically handle missing values of samples and it is much more stable than Random Forest. It also has the advantages of being highly flexible, efficient in execution and less prone to overfitting. One of its more significant disadvantages from our point of view is the very large number of parameters that can be tuned, making it more challenging to tune parameters in practice to activate the full potential of XGBoost. Support Vector Regression (SVR) is a vital application branch of Support Vector Machines (SVMs) and the basic idea behind it is to find the line of best fit. Here, we used the epsilon Support Vector Regression (Epsilon-SVR) to do the prediction. The advantages of SVR are its low computational complexity, robustness to outliers and excellent generalization ability. However, its disadvantages are that it is not suitable for large datasets and we found in experiments that the preprocessing procedures, such as standardizing, strongly influenced its performance. This makes SVR less easy to use than other methods. See the Code availability statement for details of the source code for the analyses.

Validation of the machine learning approaches

As detailed above, we used a randomly selected 10% of the original dataset as a validation set. We plotted the predicted energy expenditure from the three machine learning approaches against the actual measured energy expenditure and calculated the summed deviations to evaluate the performance of the different models (Supplementary Fig. 4). In all cases, there was a strong correlation between the predictions and the observations. The summed deviations were very similar between the different approaches, with the average absolute percentage error in the prediction being 11.6% for Random Forest, 11.4% for XGBoost and 11.5% for SVR. These are all very similar to the mean absolute error derived using the classical general linear modelling (11.2%). We then looked at the correlation of the deviations between predicted and actual data for all of the methods (the correlation matrix in Supplementary Table 1). This showed that all the approaches had correlations with the observation that were almost identical ($R = 0.82$) and the deviations between each method and the observation were very strongly correlated with each other ($R = 0.96–0.99$). In effect, the approaches were all extracting the same predictive information from the data. The error of around 11% independent of the approach exceeds the analytical error in the DLW method using the equation that we employed, which is 7.7% (ref. 21). There is consequently a gap of unexplained variation that may be possible to explain and refine the predictions. However, the similarities in the different analytical models suggest that additional predictor variables would be required to improve the model predictions.

Application to previous survey work

The NDNS is a UK government-commissioned rolling programme funded by Public Health England and the UK Food Standards Agency. The rolling programme from 2008 to 2019 is a continuous cross-sectional survey that assesses the diet, nutritional status and nutrient intake of individuals in the UK (England, Scotland, Wales and Northern Ireland) living in private households. The survey aims to collect around 1,000 samples each year, equally divided into 500 children and 500 adults, children aged 1.5–18 years and adults aged 19 years and over. There are two main stages of the survey, namely, interviewer visits and a nurse visit; all nutritional data are collected during the interviewer visits. This study used data from years 1–11 (2008–2009 to 2008–2019) for the population aged 4 and over between April 2008 and August 2019. The total number of eligible individuals included in this study was 12,694.

The NHANES database, used by the Centers for Disease Control and Prevention, contains data from a range of surveys on health and nutrition conducted since the 1960s. These surveys were initially carried out periodically between 1971 and 1994, but since 1999 the surveys have been continuous. Around 5,000 non-institutionalized US civilians are interviewed in their own homes each year and then complete a health examination. The participants fill in a questionnaire regarding their socio-economic, demographic, health-related and dietary information and are then subject to a medical examination that assesses anthropometric and laboratory measurements. A total of 5,873 participants in the NHANES (2017–2018) aged 4–80 were eligible for inclusion in the current study.

In the NDNS, the dietary intake of each participant was assessed through a 4-day food diary that measured their consumption of all foods and beverages during the study period. The respondents filled in information on their diets, including the brands of foods and drinks consumed, portion sizes, ingredients, leftovers, cooking methods and any dietary supplements that they may have taken. A parent or carer was asked to fill in the diary for children under 12 years. Sex refers to the sex of the person eating the food rather than the parent doing the coding. Older children completed the food diary themselves. Editors and coders from the NDNS team were trained to code the dietary intake information and portion sizes, and the 4-day food diaries were analysed using Diet In Nutrient Out, an integrated dietary assessment system, and the Public Health England NDNS Nutrient Databank food composition data[54,55].

The NHANES nutritional assessment included a 24-hour dietary recall interview with respondents across a range of ages. The assessments were carried out by a trained interviewer who was fluent in English and Spanish. A private room that contained a standard set of measurement guides was used for this first interview. These guides were used to assist the participants in estimating and reporting the portion sizes of their consumed foods. The measuring guides were specially designed to be used in the NHANES setting with a sample population of US civilian participants. A second dietary interview was carried out with all participants via a phone call within 3–10 days of the first interview. Spoons, measuring cups, rulers and food model booklets containing drawings of the measurement guides were provided for the participants to help them more accurately report their portion sizes during the telephone interview. Participants aged 12 years and older were able to record their intake without an assistant. For younger

persons, sex refers to the sex of the person eating the food rather than the assistant doing the coding. Food and beverages consumed were coded using the US Department of Agriculture's Food and Nutrient Database for Dietary Studies to process individuals intake (http://www.ars.usda.gov/nea/bhnrc/fsrg and https://www.cdc.gov).

Both nutritional datasets were screened to remove incomplete participant data and entered into the master spreadsheet so that the DLW equation could be applied. Children under the age of 4 were excluded and the cut-off age for classification as children was 16 years. Ethnicity data were classified according to specific categories: white, African, African living outside Africa, Asian, Hispanic, other and not available. The elevation of location was set as the average of data in the DLW dataset (158.5 m). The equation to calculate predicted TEE was applied. The tolerance interval was determined to calculate the upper and lower level of TEE to provide the accepted range within which daily energy intake must fall. Moreover, differences between energy intake and predicted energy expenditure were calculated and compared with the age and BMI of participants. Data on macronutrients (carbohydrate, protein, fat and alcohol) were converted to kilojoules and percentage of the total energy intake to compare individuals' consumption with the value of differences between energy intake and expenditure. Dietary intake from NHANES for both the first and second 24-h recall were used separately to determine whether there was an improvement in reporting with greater familiarity of the survey protocol. Then an average for both recalls was calculated and compared with the estimated energy intake. In addition, the participants of NDNS who completed all 4 days of the dietary intake survey were used to assess whether there was an improvement with time when repeating their energy intake survey.

Statistical analysis of the NDNS and NHANES datasets

Descriptive statistics for socio-demographic variables such as the mean and SD were conducted to describe both included males and females from the NDNS and NHANES participants. Data were further split into adults and children with a cut-off age of 16 years. The full DEE prediction equation will not be disclosed until publication. We calculated the number of participants whose energy intake fell within the expected variation around predicted energy expenditure. We then assessed whether there were differences in the ability to self-report energy intake with increasing age and BMI using linear regression. In addition, multiple regression was used to determine whether different dietary macronutrients (carbohydrate, protein and fat) were more likely to be under-reported. Statistical analyses were conducted using R (v4.1.3)[56] and Minitab (v19) and $p \leq 0.05$ was considered statistically significant.

Data availability

All of the data used in the derivation of the regression model are freely available via the IAEA DLW Database at https://doubly-labelled-water-database.iaea.org/home and www.dlwdatabase.org. Access to the full database must be made via an online application, but a subsample is available without restriction for free download. The NDNS data are subject to restrictions and are not available to the public. Requests to access these datasets should be directed to https://ukdataservice.ac.uk/. The NHANES data are freely available at https://wwwn.cdc.gov/nchs/nhanes/continuousnhanes/default.aspx?BeginYear=2017.

Code availability

The source code for the Python analysis is available in the Supplementary Information.

References

1. Paeratakul, S. et al. Measurement error in dietary data: implications for the epidemiologic study of the diet–disease relationship. *Eur. J. Clin. Nutr.* **52**, 722–727 (1998).
2. Hebert, J. R. et al. Systematic errors in middle-aged women's estimates of energy intake: comparing three self-report measures to total energy expenditure from doubly labeled water. *Ann. Epidemiol.* **12**, 577–586 (2002).
3. Black, A. E. & Cole, T. J. Biased over- or under-reporting is characteristic of individuals whether over time or by different assessment methods. *J. Am. Diet. Assoc.* **101**, 70–80 (2001).
4. Lutomski, J. E., van den Broeck, J., Harrington, J., Shiely, F. & Perry, I. J. Sociodemographic, lifestyle, mental health and dietary factors associated with direction of misreporting of energy intake. *Public Health Nutr.* **14**, 532–541 (2011).
5. Archer, E., Pavela, G. & Lavie, C. J. The inadmissibility of what we eat in America and NHANES dietary data in nutrition and obesity research and the scientific formulation of national dietary guidelines. *Mayo Clin. Proc.* **90**, 911–926 (2015).
6. Bernstein, D. M. & Loftus, E. F. The consequences of false memories for food preferences and choices. *Perspect. Psychol. Sci.* **4**, 135–139 (2009).
7. Speakman, J. R. Measuring energy balance: a weighty issue. *Brit. Med. J.* **371**, m4673 (2020).
8. Beaton, G., Milner, J., Mcguire, V. & Little, J. A. Sources of variance in one day dietary data—implications for design. *J. Nutr.* **109**, R22 (1979).
9. Black, A. E. Critical evaluation of energy intake using the Goldberg cut-off for energy intake:basal metabolic rate. A practical guide to its calculation, use and limitations. *Int. J. Obes. Relat. Metab. Disord.* **24**, 1119–1130 (2000).
10. Goris, A. H. & Westerterp, K. R. Underreporting of habitual food intake is explained by undereating in highly motivated lean women. *J. Nutr.* **129**, 878–882 (1999).
11. Marr, J. W. & Heady, J. A. Within- and between-person variation in dietary surveys: number of days needed to classify individuals. *Hum. Nutr. Appl. Nutr.* **40**, 347–364 (1986).
12. Prentice, A. M. et al. High levels of energy expenditure in obese women. *Br. Med. J.* **292**, 983–987 (1986).
13. Dhurandhar, N. V. et al. Energy balance measurement: when something is not better than nothing. *Int. J. Obes.* **39**, 1109–1113 (2015).
14. Hébert, J. R. et al. Considering the value of dietary assessment data in informing nutrition-related health policy. *Adv. Nutr.* **5**, 447–455 (2014).
15. Schoeller, D. A., Bandini, L. G. & Dietz, W. H. Inaccuracies in self-reported intake identified by comparison with the doubly labelled water method. *Can. J. Physiol. Pharmacol.* **68**, 941–949 (1990).
16. Black, A. E. et al. Measurements of total energy expenditure provide insights into the validity of dietary measurements of energy intake. *J. Am. Diet. Assoc.* **93**, 572–579 (1993).
17. Schoeller, D. A. Limitations in the assessment of dietary energy intake by self-report. *Metabolism* **44**, 18–22 (1995).
18. Goldberg, G. R. et al. Critical evaluation of energy intake data using fundamental principles of energy physiology: 1. Derivation of cut-off limits to identify under-recording. *Eur. J. Clin. Nutr.* **45**, 569–581 (1991).
19. Banna, J. C., McCrory, M. A., Fialkowski, M. K. & Boushey, C. Examining plausibility of self-reported energy intake data: considerations for method selection. *Front. Nutr.* **4**, 45 (2017).
20. Speakman, J. R. *Doubly Labelled Water: Theory and Practice* (Springer, 1997).
21. Speakman, J. R. et al. A standard calculation methodology for human doubly labeled water studies. *Cell Rep. Med.* **2**, 100203 (2021).
22. McCrory, M. A., Hajduk, C. L. & Roberts, S. B. Procedures for screening out inaccurate reports of dietary energy intake. *Public Health Nutr.* **5**, 873–882 (2002).

23. Vinken, A. G. et al. Equations for predicting the energy requirements of healthy adults aged 18–81 y. *Am. J. Clin. Nutr.* **69**, 920–926 (1999).
24. Speakman, J. R. et al. The International Atomic Energy Agency International Doubly Labelled Water Database: aims, scope and procedures. *Ann. Nutr. Metab.* **75**, 114–118 (2019).
25. Pontzer, H. et al. Daily energy expenditure through the human life course. *Science* **373**, 808–812 (2021).
26. Zhang, X. et al. Human total, basal and activity energy expenditures are independent of ambient environmental temperature. *iScience* **25**, 104682 (2022).
27. University of Cambridge, MRC Epidemiology Unit, NatCen Social Research. *National Diet and Nutrition Survey Years 1–11, 2008–2019* https://doi.org/10.5255/UKDA-SN-6533-19 (UK Data Service, 2021).
28. Centers for Disease Control and Prevention (CDC) & National Center for Health Statistics (NCHS). *National Health and Nutrition Examination Survey Questionnaire* https://wwwn.cdc.gov/nchs/nhanes/continuousnhanes/default.aspx?BeginYear=2017 (U.S. Depart. Health and Human Services & CDC, 2017–2018).
29. Butterfield, G. E. et al. Increased energy intake minimizes weight loss in men at high altitude. *J. Appl. Physiol.* **72**, 1741–1748 (1992).
30. Speakman, J. R. & Westerterp, K. R. Associations between energy demands, physical activity, and body composition in adult humans between 18 and 96 y of age. *Am. J. Clin. Nutr.* **92**, 826–834 (2010).
31. Speakman, J. R. & Pontzer, H. Quantifying physical activity energy expenditure based on doubly labelled water and basal metabolism calorimetry: what are we actually measuring? *Curr. Opin. Clin. Nutr. Metab. Care* **26**, 401–408 (2023).
32. Murakami, K. & Livingstone, M. B. Prevalence and characteristics of misreporting of energy intake in US children and adolescents: National Health and Nutrition Examination Survey (NHANES) 2003–2012. *Br. J. Nutr.* **115**, 294–304 (2016).
33. Berta Vanrullen, I., Volatier, J. L., Bertaut, A., Dufour, A. & Dallongeville, J. Characteristics of energy intake under-reporting in French adults. *Br. J. Nutr.* **111**, 1292–1302 (2014).
34. Kye, S. et al. Under-reporting of energy intake from 24-hour dietary recalls in the Korean National Health and Nutrition Examination Survey. *Osong Public Health Res. Perspect.* **5**, 85–91 (2014).
35. Subar, A. F. et al. Comparative validation of the Block, Willett, and National Cancer Institute food frequency questionnaires: the Eating at America's Table Study. *Am. J. Epidemiol.* **154**, 1089–1099 (2001).
36. Kroke, A. et al. Validation of a self-administered food-frequency questionnaire administered in the European Prospective Investigation into Cancer and Nutrition (EPIC) Study: comparison of energy, protein, and macronutrient intakes estimated with the doubly labeled water, urinary nitrogen, and repeated 24-h dietary recall methods. *Am. J. Clin. Nutr.* **70**, 439–447 (1999).
37. Cook, A., Pryer, J. & Shetty, P. The problem of accuracy in dietary surveys. Analysis of the over 65 UK National Diet and Nutrition Survey. *J. Epidemiol. Community Health* **54**, 611–616 (2000).
38. Lafay, L. et al. Does energy intake underreporting involve all kinds of food or only specific food items? Results from the Fleurbaix Laventie Ville Sante (FLVS) study. *Int. J. Obes. Relat. Metab. Disord.* **24**, 1500–1506 (2000).
39. Tomoyasu, N. J., Toth, M. J. & Poehlman, E. T. Misreporting of total energy intake in older African Americans. *Int. J. Obes. Relat. Metab. Disord.* **24**, 20–26 (2000).
40. Freisling, H. et al. Dietary reporting errors on 24 h recalls and dietary questionnaires are associated with BMI across six European countries as evaluated with recovery biomarkers for protein and potassium intake. *Br. J. Nutr.* **107**, 910–920 (2012).
41. Poppitt, S. D., Swann, D., Black, A. E. & Prentice, A. M. Assessment of selective under-reporting of food intake by both obese and non-obese women in a metabolic facility. *Int. J. Obes. Relat. Metab. Disord.* **22**, 303–311 (1998).
42. Bel-Serrat, S. et al. Correlates of dietary energy misreporting among European adolescents: the Healthy Lifestyle in Europe by Nutrition in Adolescence (HELENA) study. *Br. J. Nutr.* **115**, 1439–1452 (2016).
43. Previdelli, A. N. et al. Prevalence and determinants of misreporting of energy intake among Latin American populations: results from ELANS study. *Nutr. Res.* **68**, 9–18 (2019).
44. Goris, A. H. C., Westerterp-Plantenga, M. S. & Westerterp, K. R. Undereating and underrecording of habitual food intake in obese men: selective underreporting of fat intake. *Am. J. Clin. Nutr.* **71**, 130–134 (2000).
45. Thurber, C. et al. Extreme events reveal an alimentary limit on sustained maximal human energy expenditure. *Sci. Adv.* **5**, eaaw0341 (2019).
46. Westerterp, K. R., Kayser, B., Brouns, F., Herry, J. P. & Saris, W. H. Energy expenditure climbing Mt. Everest. *J. Appl. Physiol.* **73**, 1815–1819 (1992).
47. Anderson, L. et al. Assessment of energy expenditure of a professional goalkeeper from the English Premier League using the doubly labeled water method. *Int. J. Sports Physiol. Perform.* **14**, 681–684 (2019).
48. Wilson, G. et al. Energy expenditure in professional flat jockeys using doubly labelled water during the racing season: implications for body weight management. *Eur. J. Sport Sci.* **18**, 235–242 (2018).
49. Pontzer, H., Raichlen, D. A. & Sockol, M. D. Endurance versus efficiency in humans and chimpanzees: a new look at the old problem of becoming bipedal. *Am. J. Phys. Anthropol.* **27**, 173–173 (2008).
50. Gurven, M. D. et al. High resting metabolic rate among Amazonian forager–horticulturalists experiencing high pathogen burden. *Am. J. Phys. Anthropol.* **161**, 414–425 (2016).
51. Weir, J. B. New methods for calculating metabolic rate with special reference to protein metabolism. *J. Physiol.* **109**, 1–9 (1949).
52. Speakman, J. R. et al. Total daily energy expenditure has declined over the last 3 decades due to declining basal expenditure not reduced activity expenditure. *Nat. Metab.* **5**, 579–588 (2023).
53. Prentice, R. L. et al. Total energy expenditure as assessed by doubly labeled water and all-cause mortality in a cohort of postmenopausal women. *Am. J. Clin. Nutr.* **117**, 955–963 (2023).
54. Fitt, E. et al. DINO (Diet In Nutrients Out)—an integrated dietary assessment system. *Public Health Nutr.* **18**, 234–241 (2015).
55. Dikariyanto, V., Berry, S. E., Francis, L., Smith, L. & Hall, W. L. Whole almond consumption is associated with better diet quality and cardiovascular disease risk factors in the UK adult population: National Diet and Nutrition Survey (NDNS) 2008–2017. *Eur. J. Nutr.* **60**, 643–654 (2021).
56. R Core Team. R: a language and environment for statistical computing. https://www.R-project.org (R Foundation for Statistical Computing, 2022).

Acknowledgements

We are grateful to the IAEA, Taiyo Nippon Sanso and SERCON for their support and to T. Oono for his tremendous efforts at fundraising on our behalf. We are grateful to D. Tobias for many insightful comments on a previous draft of this paper. We also acknowledge contributors to the database whose data were not used in this compilation or who indicated they did not wish to be authors or who

could not be contacted. A list of these contributors is provided in the Supplementary Information. We also gratefully acknowledge funding from the Chinese Academy of Sciences (grant no. CAS 153E11KYSB20190015) and Shenzhen Key Laboratory of Metabolic Health (ZDSYS20210427152400001) awarded to J.R.S. and from the US National Science Foundation (BCS-1824466) awarded to H.P. The funders played no role in the content of this paper. The IAEA Doubly Labeled Water (DLW) Database is generously supported by the IAEA, Taiyo Nippon Sanso and SERCON.

Author contributions

R.B., C.Q.N., C.H. and J.R.S. analysed the data to derive the algorithm and performed the comparison with the NHANES and NDNS databases. Z.F.L. performed the machine learning analysis. H.A.M., L.J.A., L.A., I.B., L.B., K.B.A., E.E.B., C.V.C.B., S.B., M.S.B., N.F.B., S.G.J.A.C., R.C., G.L.C., J.A.C., R.C., S.K.D., P.S.W.D., P.D., L.R.D., S.E., U.E., S.E., T.F., B.W.F., M.G., A.H.G., M.G., A.E.H., H.H.H., D.H., M.B.H., S.M.H., N.J., A.M.J., P.K., M.K., W.E.K., W.K., R.K., R.F.K., E.V.L., P.L., C.L.L., W.R.L., N.L., M.L., C.K.M., E.M., A.C.M., J.C.M., J.P.M., A.M., M.L.N., T.A.N., C.D.N., R.M.O., K.H.P., Y.P.P., J.P.R., G.P., R.L.P, S. B. Racette, D.A.R., E.R., L.M.R., J.J.R., R.R., S. B. Roberts, D.S., L.B.S., A. M. Silva, A. M. Sjodin, M.S., E.S., S.S.U., L.M.V.E., E.G.A.H.V.M., G.W., J.A.Y., T.Y. and X.Y.Z. contributed data to the database used in the analyses. A.J.M.A., S.S., C.U.L., A.H.L., H.P., J.R., H.S., D.A.S., K.R.W., W.W.W., Y.Y. and J.R.S. contributed data to the database and were instrumental in its creation and ongoing management. R.B., C.Q.N., C.H. and J.R.S. wrote the paper. Y.Y., H.A.M., S.B., L.R.D., M.G., H.H.H., S.M.H., P.L., C.L.L., A.C.M., E.R., L.M.R., S. B. Roberts, A. M. Silva, X.Y.Z., H.P., J.R., A.M.L., H.S., D.A.S., K.R.W. and W.W.W. made extensive comments and revisions to the paper. All authors read and approved their inclusion and submission of the final version.

Competing interests

The authors declare no competing interests.

Additional information

Supplementary information The online version contains supplementary material available at https://doi.org/10.1038/s43016-024-01089-5.

Correspondence and requests for materials should be addressed to Yosuke Yamada, Amy H. Luke, Herman Pontzer, Jennifer Rood, Hiroyuki Sagayama, Dale A. Schoeller, Klaas R. Westerterp, William W. Wong or John R. Speakman.

Peer review information *Nature Food* thanks James Hébert, James Stubbs and the other, anonymous, reviewer(s) for their contribution to the peer review of this work.

Reprints and permissions information is available at www.nature.com/reprints.

Publisher's note Springer Nature remains neutral with regard to jurisdictional claims in published maps and institutional affiliations.

Open Access This article is licensed under a Creative Commons Attribution 4.0 International License, which permits use, sharing, adaptation, distribution and reproduction in any medium or format, as long as you give appropriate credit to the original author(s) and the source, provide a link to the Creative Commons licence, and indicate if changes were made. The images or other third party material in this article are included in the article's Creative Commons licence, unless indicated otherwise in a credit line to the material. If material is not included in the article's Creative Commons licence and your intended use is not permitted by statutory regulation or exceeds the permitted use, you will need to obtain permission directly from the copyright holder. To view a copy of this licence, visit http://creativecommons.org/licenses/by/4.0/.

© The Author(s) 2025

Rania Bajunaid[1,2,81], Chaoqun Niu[3,4,81], Catherine Hambly[1,81], Zongfang Liu[3,81], Yosuke Yamada[5,6] ✉, Heliodoro Aleman-Mateo[7], Liam J. Anderson[8], Lenore Arab[9], Issad Baddou[10], Linda Bandini[11], Kweku Bedu-Addo[12], Ellen E. Blaak[13], Carlijn V. C. Bouten[13], Soren Brage[14], Maciej S. Buchowski[15], Nancy F. Butte[16], Stefan G. J. A. Camps[13], Regina Casper[17], Graeme L. Close[18], Jamie A. Cooper[19], Richard Cooper[20], Sai Krupa Das[21], Peter S. W. Davies[22], Prasangi Dabare[23], Lara R. Dugas[20,24], Simon Eaton[25], Ulf Ekelund[26], Sonja Entringer[27,28], Terrence Forrester[29], Barry W. Fudge[30], Melanie Gillingham[31], Annelies H. Goris[32], Michael Gurven[33], Asmaa El Hamdouchi[10], Hinke H. Haisma[34], Daniel Hoffman[35], Marije B. Hoos[13], Sumei Hu[3], Noorjehan Joonas[36], Annemiek M. Joosen[13], Peter Katzmarzyk[37], Misaka Kimura[38], William E. Kraus[39], Wantanee Kriengsinyos[40], Rebecca Kuriyan[41], Robert F. Kushner[42], Estelle V. Lambert[43], Pulani Lanerolle[44], Christel L. Larsson[45], William R. Leonard[46], Nader Lessan[47,48], Marie Löf[49,50], Corby K. Martin[37], Eric Matsiko[51], Anine C. Medin[52,53], James C. Morehen[18], James P. Morton[18], Aviva Must[54], Marian L. Neuhouser[55], Theresa A. Nicklas[16], Christine D. Nyström[50], Robert M. Ojiambo[56,57], Kirsi H. Pietiläinen[58], Yannis P. Pitsiladis[59], Jacob Plange-Rhule[12], Guy Plasqui[60], Ross L. Prentice[55], Susan B. Racette[61], David A. Raichlen[62], Eric Ravussin[37], Leanne M. Redman[37], John J. Reilly[63], Rebecca Reynolds[64], Susan B. Roberts[65], Dulani Samaranayakem[66], Luis B. Sardinha[67], Analiza M. Silva[67], Anders M. Sjödin[68], Marina Stamatiou[1], Eric Stice[69], Samuel S. Urlacher[70,71], Ludo M. Van Etten[13], Edgar G. A. H. van Mil[72], George Wilson[18], Jack A. Yanovski[73], Tsukasa Yoshida[38,74], Xueying Zhang[1,3], Alexia J. Murphy-Alford[75], Srishti Sinha[75], Cornelia U. Loechl[75], Amy H. Luke[20] ✉, Herman Pontzer[76,77] ✉, Jennifer Rood[37] ✉, Hiroyuki Sagayama[78] ✉, Dale A. Schoeller[79] ✉, Klaas R. Westerterp[13] ✉, William W. Wong[16] ✉ & John R. Speakman[1,3,4,80,81] ✉

[1]School of Biological Sciences, University of Aberdeen, Aberdeen, UK. [2]Food and Nutrition Department, King Abdulaziz University, Jeddah, Saudi Arabia. [3]Shenzhen Key Laboratory of Metabolic Health, Center for Energy Metabolism and Reproduction, Shenzhen Institute of Advanced Technology, Chinese Academy of Sciences, Shenzhen, China. [4]State Key Laboratory of Molecular Developmental Biology, Institute of Genetics and Developmental Biology, Chinese Academy of Sciences, Beijing, China. [5]Graduate School of Biomedical Engineering, Tohoku University, Sendai, Japan. [6]Department of Medicine

and Science in Sports and Exercise, Graduate School of Medicine, Tohoku University, Sendai, Japan. [7]Department of Nutrition and Metabolism, Nutrition Coordination, Research Center for Food and Development (CIAD), Hermosillo, Mexico. [8]School of Sport, Exercise and Rehabilitation Sciences, University of Birmingham, Birmingham, UK. [9]David Geffen School of Medicine, University of California, Los Angeles, CA, USA. [10]Unité Mixte de Recherche en Nutrition et Alimentation, CNESTEN-Université Ibn Tofail URAC39, Regional Designated Center of Nutrition Associated with AFRA/IAEA, Rabat, Morocco. [11]University of Massachusetts Chan Medical School, Worcester, MA, USA. [12]Department of Physiology, Kwame Nkrumah University of Science and Technology, Kumasi, Ghana. [13]Department of Human Biology, NUTRIM, School for Metabolism and Translational Research in Metabolism, Maastricht University Medical Centre+, Maastricht, The Netherlands. [14]MRC Epidemiology Unit, University of Cambridge, Cambridge, UK. [15]Division of Gastroenterology, Hepatology and Nutritiion, Department of Medicine, Vanderbilt University, Nashville, TN, USA. [16]Department of Pediatrics, Baylor College of Medicine, USDA/ARS Children's Nutrition Research Center, Houston, TX, USA. [17]Department of Psychiatry and Behavioral Sciences, Stanford University School of Medicine, Stanford, CA, USA. [18]Research Institute for Sport and Exercise Sciences, Liverpool John Moores University, Liverpool, UK. [19]Nutritional Sciences, University of Wisconsin, Madison, WI, USA. [20]Department of Public Health Sciences, Parkinson School of Health Sciences and Public Health, Loyola University, Maywood, IL, USA. [21]USDA Human Nutrition Research Center on Aging at Tufts University, Boston, MA, USA. [22]Child Health Research Centre, Centre for Children's Health Research, University of Queensland, South Brisbane, Queensland, Australia. [23]Department of Physiotherapy, Faculty of Allied Health Sciences, General Sir John Kotelawala Defence University, Kandawala, Sri Lanka. [24]Division of Epidemiology and Biostatistics, School of Public Health, University of Cape Town, Cape Town, South Africa. [25]Developmental Biology and Cancer Department, UCL Great Ormond Street Institute of Child Health, London, UK. [26]Department of Sport Medicine, Norwegian School of Sport Sciences, Oslo, Norway. [27]Charité–Universitätsmedizin Berlin, corporate member of Freie Universität Berlin and HumboldtUniversität zu Berlin, Institute of Medical Psychology, Berlin, Germany. [28]Department of Pediatrics, School of Medicine, University of California Irvine, Irvine, CA, USA. [29]Solutions for Developing Countries, University of the West Indies, Kingston, Jamaica. [30]Department of Biomedical and Life Sciences, University of Glasgow, Glasgow, UK. [31]Department of Molecular and Medical Genetics, Oregon Health and Science University, Portland, OR, USA. [32]Imec, OnePlanet Research Center, Wageningen, The Netherlands. [33]Department of Anthropology, University of California Santa Barbara, Santa Barbara, CA, USA. [34]Population Research Centre, Faculty of Spatial Sciences, University of Groningen, Groningen, The Netherlands. [35]Department of Nutritional Sciences, Program in International Nutrition, Rutgers University, New Brunswick, NJ, USA. [36]Central Health Laboratory, Ministry of Health and Wellness, Candos, Mauritius. [37]Pennington Biomedical Research Center, Baton Rouge, LA, USA. [38]Institute for Active Health, Kyoto University of Advanced Science, Kyoto, Japan. [39]Department of Medicine, Duke University, Durham, NC, USA. [40]Institute of Nutrition, Mahidol University, Nakhon-Pathom, Thailand. [41]Division of Nutrition, St. John's Research Institute, Bangalore, India. [42]Feinberg School of Medicine, Northwestern University, Chicago, IL, USA. [43]Health through Physical Activity, Lifestyle and Sport Research Centre (HPALS), Division of Exercise Science and Sports Medicine (ESSM), FIMS International Collaborating Centre of Sports Medicine, Department of Human Biology, Faculty of Health Sciences, University of Cape Town, Cape Town, South Africa. [44]Department of Biochemistry and Molecular Biology, Faculty of Medicine, University of Colombo, Colombo, Sri Lanka. [45]Department of Food and Nutrition and Sport Science, University of Gothenburg, Gothenburg, Sweden. [46]Department of Anthropology, Northwestern University, Evanston, IL, USA. [47]Imperial College London Diabetes Centre, Abu Dhabi, United Arab Emirates. [48]Department of Metabolism, Digestion and Reproduction, Imperial College London, London, UK. [49]Department of Health, Medicine and Caring Sciences, Linköping University, Linköping, Sweden. [50]Department of Biosciences and Nutrition, Karolinska Institute, Stockholm, Sweden. [51]UR Sweden Program, University of Rwanda, Kigali, Rwanda. [52]Department of Nutrition, Institute of Basic Medical Sciences, University of Oslo, Oslo, Norway. [53]Department of Nutrition and Public Health, Faculty of Health and Sport Sciences, University of Agder, Kristiansand, Norway. [54]Tufts University School of Medicine, Boston, MA, USA. [55]Division of Public Health Sciences, Fred Hutchinson Cancer Center and School of Public Health, University of Washington, Seattle, WA, USA. [56]Kenya School of Medicine, Moi University, Eldoret, Kenya. [57]Rwanda Division of Basic Sciences, University of Global Health Equity, Kigali, Rwanda. [58]Obesity Research Unit, Research Program for Clinical and Molecular Metabolism, Faculty of Medicine, University of Helsinki and Abdominal Center, Obesity Center, HealthyWeightHub, Helsinki University Hospital and University of Helsinki, Helsinki, Finland. [59]School of Sport and Service Management, University of Brighton, Eastbourne, UK. [60]Department of Nutrition and Movement Sciences, Maastricht University, Maastricht, The Netherlands. [61]College of Health Solutions, Arizona State University, Phoenix, AZ, USA. [62]Biological Sciences and Anthropology, University of Southern California, California, CA, USA. [63]School of Psychological Sciences and Health, University of Strathclyde, Glasgow, UK. [64]Centre for Cardiovascular Sciences, Queen's Medical Research Institute, University of Edinburgh, Edinburgh, UK. [65]Department of Medicine and Department of Epidemiology, Geisel School of Medicine at Dartmouth College, Hanover, NH, USA. [66]Department of Community Medicine, Faculty of Medicine, University of Colombo, Colombo, Sri Lanka. [67]Exercise and Health Laboratory, CIPER, Faculdade Motricidade Humana, Universidade de Lisboa, Lisbon, Portugal. [68]Department of Nutrition, Exercise and Sports, Copenhagen University, Copenhagen, Denmark. [69]Department of Psychiatry and Behavioural Sciences, Stanford University, Stanford, CA, USA. [70]Department of Anthropology, Baylor University, Waco, TX, USA. [71]Child and Brain Development Program, CIFAR, Toronto, Ontario, Canada. [72]Maastricht University, Brightlands Campus Greenport Venlo and Lifestyle Medicine Center for Children, Jeroen Bosch Hospital, 's-Hertogenbosch, The Netherlands. [73]Section on Growth and Obesity, Division of Intramural Research, Eunice Kennedy Shriver National Institute of Child Health and Human Development, National Institutes of Health Bethesda, Bethesda, MD, USA. [74]National Institute of Health and Nutrition, National Institutes of Biomedical Innovation, Health and Nutrition, Osaka, Japan. [75]Department of Nutritional Sciences, International Atomc Energy Agency, Vienna, Austria. [76]Duke Global Health Institute, Duke University, Durham, NC, USA. [77]Department of Evolutionary Anthropology, Duke University, Durham, NC, USA. [78]Faculty of Health and Sport Sciences, University of Tsukuba, Ibaraki, Japan. [79]Biotechnology Center and Department of Nutritional Sciences, University of Wisconsin, Madison, WI, USA. [80]Institute of Health Sciences, China Medical University, Shenyang, China. [81]These authors contributed equally: Rania Bajunaid, Chaoqun Niu, Catherine Hambly, Zongfang Liu, John R. Speakman. ✉e-mail: yyamada831@gmail.com; aluke@luc.edu; pontzer@gmail.com; Jennifer.rood@pbrc.edu; sagayama.hiroyuki.ka@u.tsukuba.ac.jp; dschoell@nutrisci.wisc.edu; k.westerterp@maastrichtuniversity.nl; wwong@bcm.edu; j.speakman@abdn.ac.uk

nature food

Supplementary information https://doi.org/10.1038/s43016-024-01089-5

Predictive equation derived from 6,497 doubly labelled water measurements enables the detection of erroneous self-reported energy intake

In the format provided by the authors and unedited

Table of Contents

Supplementary Tables.......... 2
Supplementary Figures 11
Supplementary Methods 15
Contributing non-authors 19

Supplementary Tables

Table S1: Correlation matrix of the predictions against the observed data using the four different analytical approaches and the correlations between the deviations of the predictions from the actual observations across the four different analytical approaches.

	Multi Linear	Random Forest	XGBoost	SVM	Observed
Multi Linear	1	0.953468	0.976156	0.946645	0.815885
Rand Forest	0.953468	1	0.980513	0.904719	0.819497
XGBoost	0.976156	0.980513	1	0.926833	0.820515
SVM	0.946645	0.904719	0.926833	1	0.760951
Observed	0.815885	0.819497	0.820515	0.760951	1

Table S2. Descriptive statistics: sociodemographic characteristics of the study participants

		NDNS		NHANES	
**Age**		**Mean**	**±SD**	**Mean**	**±SD**
	**male**	30.8	23.3	39.5	23.7
	**female**	33.9	23.2	39.1	23.2
**Sex**		**N**	**%**	**N**	**%**
	**male**	5799	45.7	2819	48
	**female**	6895	54.3	3054	52.0
**BMI**		**Mean**	**±SD**	**Mean**	**±SD**
	**male**	23.605	6.135	26.991	7.436
	**female**	24.4	6.5	28.	8.7
**Ethnicity**		**N**	**%**	**N**	**%**
**White**		11914	93.86	2052	34.94
**African heritage persons living outside Africa**		-	-	1444	24.59
**African living in Africa**		-	-	-	-
**Asian**		600	4.73	680	11.58
**Hispanic**		-	-	1310	22.31
**Other**		180	1.42	387	6.59
**Not available**		-	-	-	-

SD= standard deviation, N= total number, %= percentage, BMI (Body Mass Index)

Table S3: The number of participants classified as under and over reporters% in the NDNS dataset years (1-11) and NHANES datasets year (2017-2018) using Goldberg, Black, and McCrory equations.

	NDNS				NHANES			
	Goldberg cut-off1				Goldberg cut-off1			
	Under%				Under%			
sex	M	F			M	F		
Day1	24.00	26.87			20.01	22.02		
Day2	25.63	27.60			27.54	28.90		
Day3	26.47	28.51						
Day 4	28.52	30.64						
	Goldberg cut-off 2				Goldberg cut-off 2			
Average days	39.36	41.42			24.17	27.13		
	Black				Black			
	Under%		Over%		Under%		Over%	
sex	M	F	M	F	M	F	M	F
Day1	32.22	36.26	1.35	1.08	26.05	28.51	6.00	3.67
Day2	34.12	36.53	1.31	1.11	34.74	36.24	3.51	2.62
Day3	35.05	37.78	1.38	1.14				
Day4	37.25	39.86	1.19	1.35				
Average days	29.84	33.33	0.40	0.34	25.87	28.44	2.98	1.80
	McCrory cut-off1				McCrory cut-off1			
	Under%				Under%			
sex	M	F			M	F		
Day1	66.24	68.59			73.64	72.51		
Day2	68.59	66.22			67.74	63.59		
Day3	65.13	65.77						
Day4	63.36	62.47						
Average days	71.12	70.53			76.66	74.3		
	McCrory cut-off2				McCrory cut-off2			
	Under%				Under%			
sex	M	F			M	F		
Day1	23.72	25.58			35.61	33.51		
Day2	23.29	25.10			29.32	29.09		
Day3	23.55	24.65						
Day4	22.08	22.61						
Average days	20.48	22.59			34.96	31.86		

Table S4A: Comparison between DEE calculated using the DLW equation and self-reported DEI in KJ in the NDNS dataset years (1-11) for subjects who completed 4 days. Energy intake refers to the intake on the particular comparison day.

Days		Averaged Energy intake	within range (N)	within range (%)
1 day	Male children	7338.59	1337	54.37
	Male adult	8655.91	1212	37.35
	Female children	6450.81	1308	56.02
	Female adult	6715.27	1675	37.84
2 day	Male children	7168.35	1296	52.70
	Male adult	8660.30	1199	36.95
	Female children	6339.73	1250	53.53
	Female adult	6669.65	1704	38.49
3 day	Male children	7094.99	1297	52.75
	Male adult	8623.95	1198	36.92
	Female children	6186.92	1208	51.73
	Female adult	6670.63	1667	37.66
4 day	Male children	6976.02	1218	49.53
	Male adult	8420.57	1117	34.42
	Female children	6136.06	1154	49.42
	Female adult	6533.40	1576	35.60

Table S4B: Comparison between DEE calculated using the DLW equation and self-reported DEI in KJ in the NDNS dataset years (1-11) for subjects who completed 4 days using averaged intake for days. Average intake refers to the average intake over the given day and all previous survey days.

Averaged days		Averaged Energy intake	within range (N)	within range (%)
1 day	Male children	7338.59	1337	54.37
	Male adult	8655.91	1212	37.35
	Female children	6450.81	1308	56.02
	Female adult	6715.27	1675	37.84
2 days	Male children	7253.47	1413	57.46
	Male adult	8658.10	1220	37.60
	Female children	6395.27	1385	59.31
	Female adult	6692.46	1730	39.08
3 days	Male children	7200.64	1387	56.41
	Male adult	8646.72	1215	37.44
	Female children	6325.82	1389	59.49
	Female adult	6685.19	1722	38.90
4 days	Male children	7144.49	1391	56.57
	Male adult	8590.18	1189	36.64
	Female children	6278.38	1377	58.97
	Female adult	6647.24	1691	38.20

Supplementary Table S5: Comparison between DEE calculated using the DLW equation and self-reported DEI in KJ in the NHANES dataset year (2017-2018) for subjects who completed 24-hour recalls and averaged two recalls.

Days		Averaged Energy intake	within range (N)	within range (%)
1 day	Male children	8754.90	376	52.96
	Male adult	10149.91	933	44.26
	Female children	7335.42	381	50.53
	Female adult	7668.29	991	43.12
2 day	Male children	7721.85	318	44.79
	Male adult	9184.49	809	38.38
	Female children	6846.01	359	47.61
	Female adult	6922.25	853	37.12
Average 2 days	Male children	8238.38	408	57.46
	Male adult	9667.20	947	44.92
	Female children	7079.49	426	56.50
	Female adult	7295.27	981	42.69

Table S6: Deviations between the predicted and observed energy expenditures for specific groups not included in derivation of the equation.

**Athletes and usual activity**	**mean diff (MJ/d)**	**sd**	**n**
Race across America participants	11.3	6.71	18
Cross country skiers	17.6	5.97	8
Flat course jockeys	3.34	1.96	15
Male rugby league players	10.7	2.31	6
Male football (soccer) players	4.31	2.62	8
Adolescent track and field amateurs	6.82	2.52	190
**Reproductive females**			
Pregnancy trimester 2	2.53	2.51	21
Pregnancy trimester 3	3.53	2.52	71
Lactation	5.25	3.28	86

Table S7 Example calculations for two hypothetical subjects

Subject 1: Hispanic male living in Boston, USA (elevation 14m). Age 41, weight 85 kgs, 1.83m tall. Self reported intake 12 MJ/day.

variable		coefficient	variable x coefficient	
constant			-0.2172	
primary variables				
Ln (BW) kg	4.442651	0.4167	1.851253	
Height cm	183	0.006565	1.201395	
age y	41	-0.02054	-0.84214	
age2	1681	0.0003308	0.556075	
age3	68921	-0.000001852	-0.12764	
Ln(elevation) m	2.639057	0.09126	0.24084	
Sex	-1	-0.04092	0.04092	
ethnicity				
A	0	0.0194	0	
AA	0	-0.03899	0	
AS	0	0.006238	0	
C	0	0.02626	0	
H	1	-0.0155	-0.0155	
NA	0	0.003598	0	
interactions				
height x Ln(elevation)	482.9475	-0.0006759	-0.32642	
age x Ln(elevation)	108.2014	0.002018	0.21835	
age2 x Ln(elevation)	4436.255	-0.00002262	-0.10035	
sex x Ln(elevation)	-2.63906	-0.006947	0.018334	
summed values			2.497913	
exponent of summed values			12.15709	MJ/day
			2905.615	kcal/day

lower 95% PI	7.535986	MJ/d
upper 95% PI	19.05123	MJ/d

Person reports eating 12 MJ/day in survey. Since this is inside the 95% predictive interval this person would not be classed as misreporting their food intake.

Subject 2; Asian female, living in Aberdeen UK (elevation 39m), 1.51m tall, 22 years old. Self reported intake 4.15 MJ/d

variable		coefficient	variable x coefficient	
constant			-0.2172	
primary variables				
Ln (BW) kg	3.839452	0.4167	1.5999	
Height cm	151	0.006565	0.991315	
age y	22	-0.02054	-0.45188	
age2	484	0.0003308	0.160107	
age3	10648	-0.000001852	-0.01972	
Ln(elevation) m	3.663562	0.09126	0.334337	
Sex	1	-0.04092	-0.04092	
ethnicity				
A	0	0.0194	0	
AA	0	-0.03899	0	
AS	1	0.006238	0.006238	
C	0	0.02626	0	
H	0	-0.0155	0	
NA	0	0.003598	0	
interactions				
height x Ln(elevation)	553.1978	-0.0006759	-0.37391	
age x Ln(elevation)	80.59836	0.002018	0.162647	
age2 x Ln(elevation)	1773.164	-0.00002262	-0.04011	
sex x Ln(elevation)	3.663562	-0.006947	-0.02545	
summed values			2.085358	
exponent of summed values			8.047471	MJ/d
			1923.392	kcal/d
lower 95% PI			4.467742	MJ/d
upper 95% PI			13.54639	MJ/d

Person reports eating 4.15 MJ/day in survey. This is below the lower 95% predictive interval and so this person would be excluded as an under-reporter.

Supplementary Figures

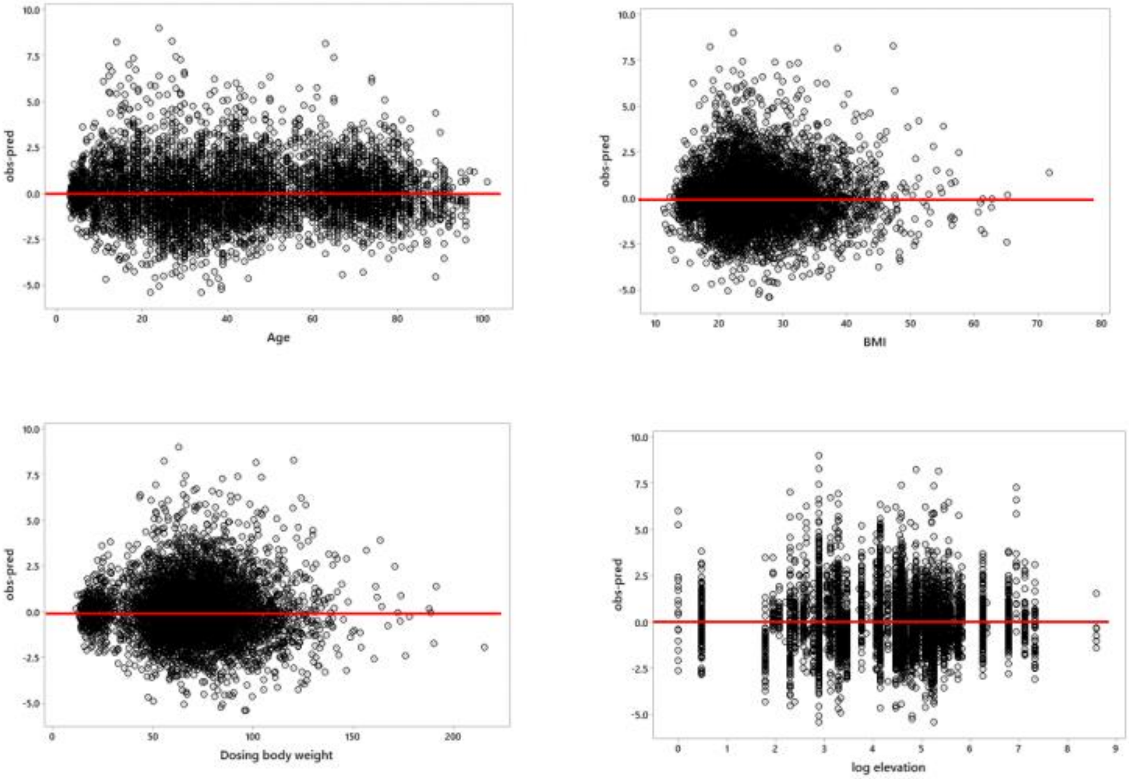

Figure S1 Residual error in the predicted energy expenditure plotted against A: age, B: BMI, C: body weight and D) log elevation for the 5828 data used in the derivation of the prediction. There was no indication of systematic bias with respect to any of the predictor variables. The line of no difference is shown in red.

A

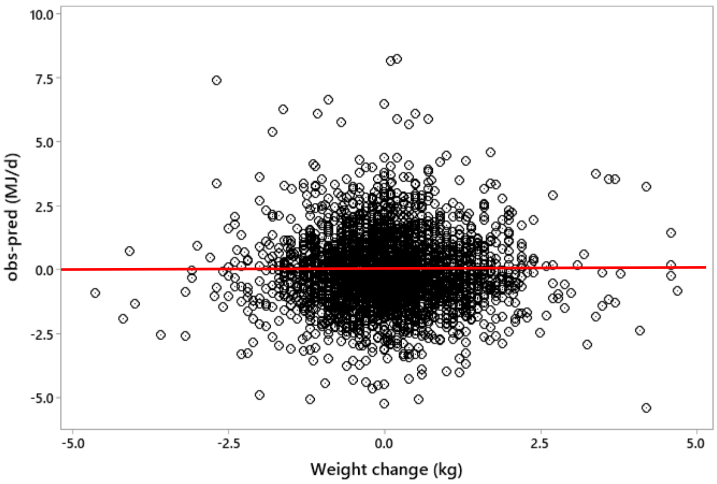

B

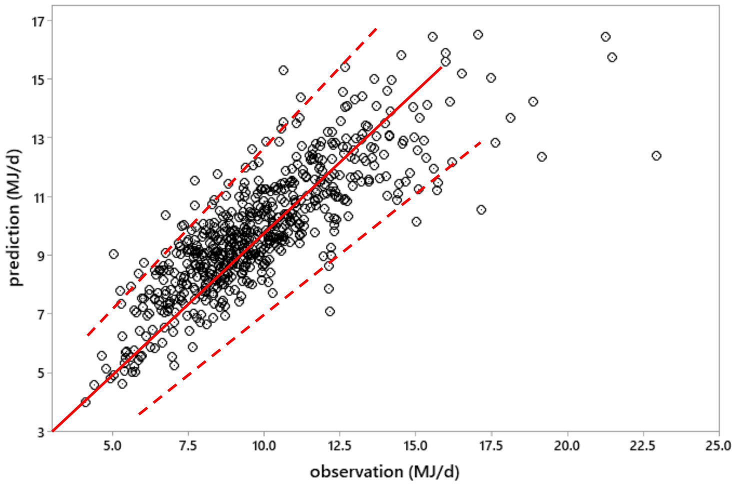

Figure S2: A: Residual error in the predicted energy expenditure plotted against change in body weight over the measurement period for 3088 individuals where weight change was reported. Seventeen individuals changed by more than 5 kgs and are not illustrated in this plot. There was no significant relationship whether these data were included or excluded. Line of no change in weight in red. B: observed TEE (MJ/d) plotted against the predicted TEE from the model for the 598 subjects in the test

data set. The line of identity is shown in red. The upper and lower 95% predictive intervals around the mean are shown dashed. 94.4% of the data fell inside the predictive interval.

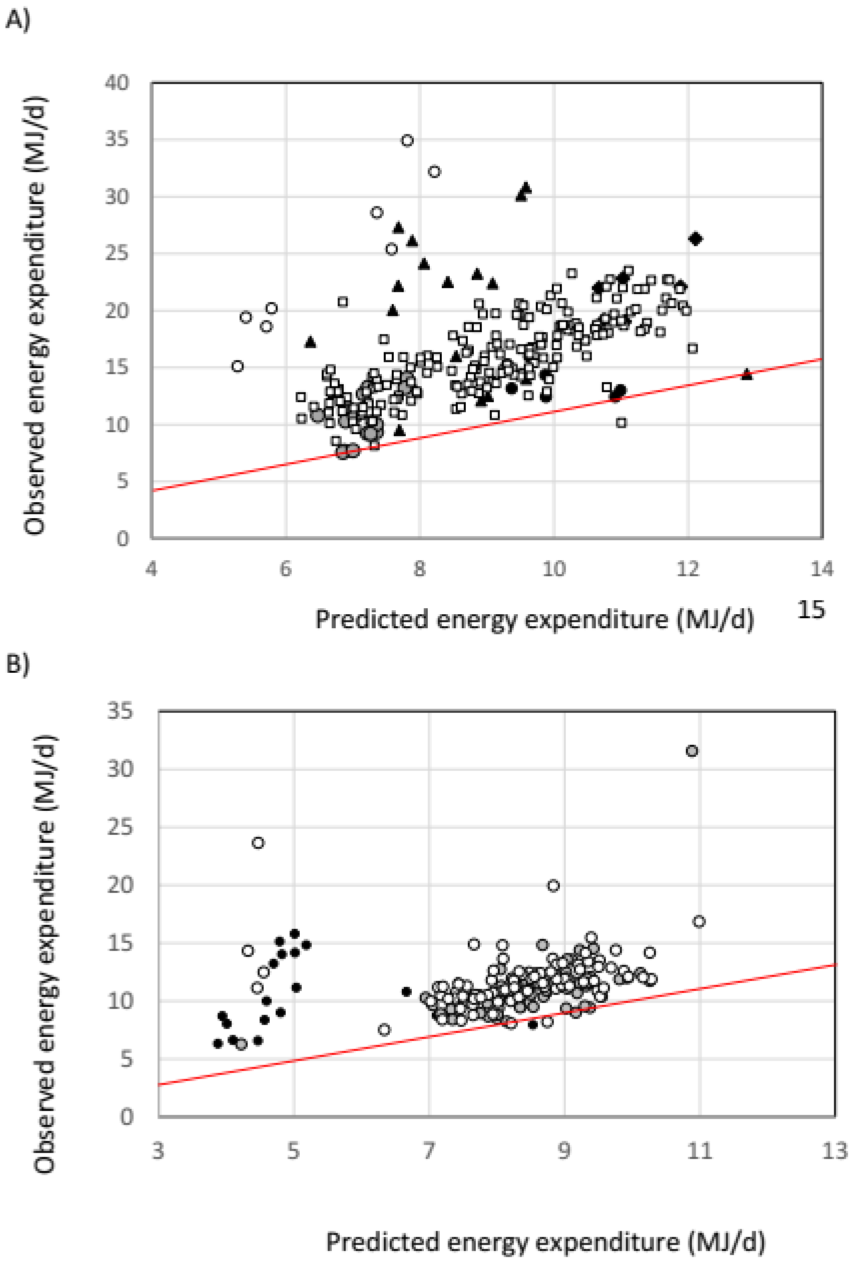

Figure S3: A: predicted and observed TEE for 246 athletes and individuals engaged in usual activities. Open circles cross country skiers, black triangles race across America participants, open squares, Portuguese track and field athletes, black circles male football (soccer) players, black diamonds male rugby league players, grey circles flat course jockeys. Line of identity shown in red. B: predicted and

observed TEE for 176 females in reproduction. Open circles pregnancy trimester 2, grey circles pregnancy trimester 3, black circles, lactation. Lactating sample mostly from rural Africa. Line of identity shown in red.

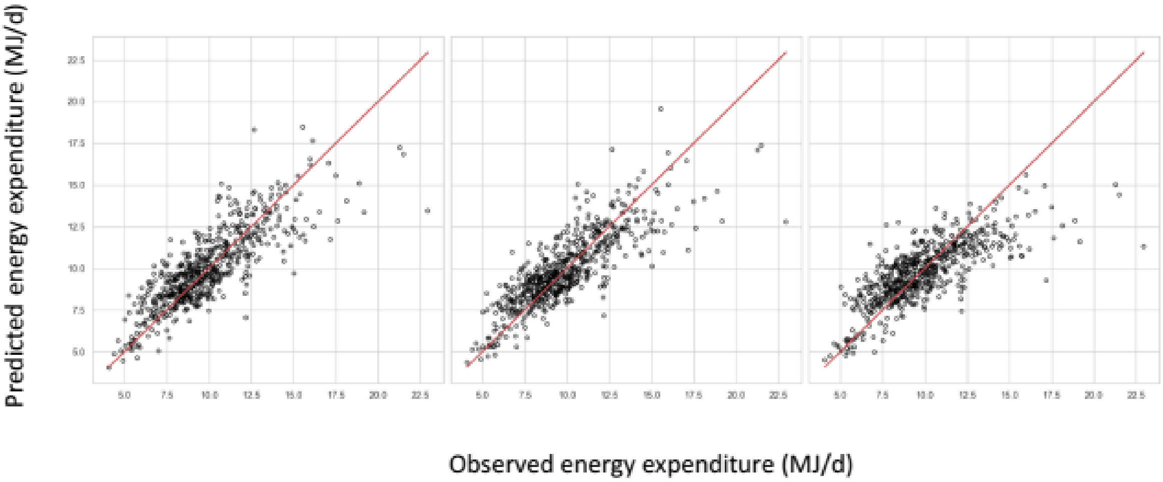

Figure S4: Predicted energy expenditure (MJ/d) from the three machine learning approaches A Random forest, B: XGBoost and C: SVR plotted against the actual observed expenditure (MJ/d) for a sample of 598 randomly selected individuals. The line of identity in each case is shown in red.

Supplementary Methods

Source code for machine learning analysis in Python

Data were pre-processed using the following script

```
#!/usr/bin/env python
# -*- encoding: utf-8 -*-
"""
@File    :   pre_processor.py
@Contact :   zongfangliu00@gmail.com
@License :   (C)Copyright 2022-2023, ZongfangLiu

@Modify Time      @Author    @Version    @Description
------------      -------    --------    -----------
2023/11/14 16:37   zfliu      1.0         None
"""
import pandas as pd


def standardize(all_features):

    numeric_features_index = all_features.dtypes[all_features.dtypes !=
'object'].index

    dic = 15
    for item in all_features[numeric_features_index]:
        dic[item] = (all_features[item].mean(), all_features[item].std())

    all_features[numeric_features_index] =
all_features[numeric_features_index].apply(
        lambda x: (x - x.mean()) / (x.std()))

    all_features[numeric_features_index] =
all_features[numeric_features_index].fillna(0)

    return all_features, dic


def standardize_test(all_features_test, parameter_dic):

    numeric_features_index =
all_features_test.dtypes[all_features_test.dtypes != 'object'].index

    for item in parameter_dic:
        all_features_test[item] = all_features_test[item].apply(
            (lambda x: (x - parameter_dic[item][0]) /
(parameter_dic[item][1])))

    all_features_test[numeric_features_index] =
all_features_test[numeric_features_index].fillna(0)

    return all_features_test
```

```
def one_hot(all_features):

    all_features = pd.get_dummies(all_features, dummy_na=True)

    return all_features


def shuffle(data):
    data = data.sample(frac=1, random_state=42).reset_index(drop=True)
    return data
```

Analysis was completed using the following script.

```
#!/usr/bin/env python
# -*- encoding: utf-8 -*-
"""
@File    :   Tee_data_analysis.py
@Contact :   zongfangliu00@gmail.com
@License :   (C)Copyright 2022-2023, ZongfangLiu

@Modify Time      @Author    @Version    @Description
------------      -------    --------    -----------
2023/11/14 16:37   zfliu      1.0         None
"""
from src.data_processing import pre_processor
import pandas as pd
from sklearn.linear_model import LinearRegression
import xgboost as xgb
from sklearn.ensemble import RandomForestRegressor
from sklearn.svm import SVR
import numpy as np
from matplotlib import pyplot as plt
import seaborn as sns


train_data =
pd.read_csv(r"/Users/liuzongfang/Documents/Codes/TEE_Prediction/codes/TEE_pap
er_exps/data/original_data/train_set.csv",
na_values="NaN")#.values.astype(np.float32)
test_data =
pd.read_csv(r"/Users/liuzongfang/Documents/Codes/TEE_Prediction/codes/TEE_pap
er_exps/data/original_data/test_set.csv",
na_values="NaN")#.values.astype(np.float32)

# Must do one hot
do_shuffle, do_standardize, do_onehot, do_drop = True, False, True, False


if do_drop:
    train_data = train_data.drop(['Sex', 'ethnicity'], axis=1, inplace=False)
    test_data = test_data.drop(['Sex', 'ethnicity'], axis=1, inplace=False)

all_features = train_data.iloc[:, :-1]
TEE = train_data.iloc[:, -1]
all_features_test = test_data.iloc[:, :-1]
TEE_test = test_data.iloc[:, -1]
```

```python
if do_standardize:

    all_features, parameter_dic = pre_processor.standardize(all_features)

    all_features_test = pre_processor.standardize_test(all_features_test,
parameter_dic)


if do_onehot:

    all_features = pre_processor.one_hot(all_features)
    all_features_test = pre_processor.one_hot(all_features_test)

# Replace the missing values as mean values
indexes = all_features.columns.values
for item in indexes:
    cur_mean = all_features[item].mean()
    all_features[item].fillna(value=cur_mean, inplace=True)
    cur_mean_test = all_features_test[item].mean()
    all_features_test[item].fillna(value=cur_mean, inplace=True)

train_data = pd.concat((all_features, TEE), axis=1)
test_data = pd.concat((all_features_test, TEE_test), axis=1)

if do_shuffle:

    train_data = pre_processor.shuffle(train_data)
#%%
train_data = train_data.values.astype(np.float32)
test_data = test_data.values.astype(np.float32)
x_train, y_train = train_data[:, :-1], train_data[:, -1]
x_test, y_test = test_data[:, :-1], test_data[:, -1]

random_forest = RandomForestRegressor()
xgboost = xgb.XGBRegressor(
    eta=0.05,
    max_depth=6,
    seed=42,
)
svr = SVR()

random_forest.fit(x_train, y_train)
xgboost.fit(x_train, y_train)
svr.fit(x_train, y_train)

rf_pred = random_forest.predict(x_test)
xgb_pred = xgboost.predict(x_test)
svr_pred = svr.predict(x_test)

sns.set(style='whitegrid')
colors = np.array([0, 0, 0])

fig, [ax0, ax1, ax2] = plt.subplots(1, 3, figsize=(18, 6), sharey=True,
constrained_layout=True)

ax0.scatter(y_test, rf_pred, s=18, facecolors='none', edgecolors=colors)
```

```
ax0.plot(y_test, y_test, 'r')

ax1.scatter(y_test, xgb_pred, s=18, facecolors='none', edgecolors=colors)
ax1.plot(y_test, y_test, 'r')

ax2.scatter(y_test, svr_pred, s=18, facecolors='none', edgecolors=colors)
ax2.plot(y_test, y_test, 'r')

plt.show()

def MAPE(y, y_pred):
    return sum(abs(y - y_pred) / y) / len(y)

mape_values = {}
mape_values["RandomForest"] = MAPE(y_test, rf_pred)
mape_values["Xgboost"] = MAPE(y_test, xgb_pred)
mape_values["SVR"] = MAPE(y_test, svr_pred)
print(mape_values)
```

The IAEA DLW database group authorship (database version 3.7).

This group authorship contains the names of some researchers who did not assent inclusion to the main authorship because they felt their contribution was not sufficient to merit authorship, people whose data were contributed into the IAEA DLW database by the analysis laboratory but they later could not be traced, or they did not respond to emails to assent inclusion among the authorship.

Dr Philip N. Ainslie
Research Institute for Sport and Exercise Sciences, Liverpool John Moores University, Liverpool, UK

Dr Lene F. Andersen
Department of Nutrition, Institute of Basic Medical Sciences, University of Oslo, 0317 Oslo, Norway.

Dr Stephane Blanc
Nutritional Sciences, University of Wisconsin, Madison, WI, USA
Institut Pluridisciplinaire Hubert Curien. CNRS Université de Strasbourg, UMR7178, France.

Dr Alberto G. Bonomi
Maastricht University, Maastricht, The Netherlands

Dr Pascal Bovet
Institute of Social and Preventive Medicine, Lausanne University Hospital, Lausanne, Switzerland.

Dr Stefan Branth
University of Uppsala, Uppsala, Sweden

Dr Niels C. De Bruin
Erasmus University, Rotterdam, The Netherlands

Dr Lisa H. Colbert
Kinesiology, University of Wisconsin, Madison, WI,

Dr Dan Cummings

Dr William Dietz
George Washington University, Washington DC, US

Dr Alice E. Dutman
TNO Quality of Life, Zeist, The Netherlands

Dr Cara B. Ebbeling
Boston Children's Hospital, Boston, Massachusetts, USA

Dr Sölve Elmståhl
Lund University, Lund, Sweden

Dr Mikael Fogelholm
Dept of Food and Nutrition, Helsinki, Finland

Dr Tamara Harris
Aging, NIH, Bethesda, MD,

Dr Rik Heijligenberg
Academic Medical Center of Amsterdam University, Amsterdam, The Netherlands

Dr Hans U. Jorgensen
Bispebjerg Hospital, Copenhagen, Denmark

Dr Kitty P. Kempen
Maastricht University, Maastricht, The Netherlands

Dr David S. Ludwig
Boston Children's Hospital, Boston, Massachusetts, USA

Dr Margaret McCloskey
Royal Belfast Hospital for Sick Children, Belfast, Northern Ireland

Dr Erwin P. Meijer
Phillips Research, Eindhoven, The Netherlands

Dr Gerwin A. Meijer
Maastricht University, Maastricht, The Netherlands

Dr Daphne L. Pannemans
Maastricht University, Maastricht, The Netherlands

Dr Renaat M. Philippaerts
Katholic University Leuven, Leuven, Belgium

Dr Roberto A. Rabinovich
The Queen's medical research Institute, University of Edinburgh, Edinburgh, UK.

Dr Elisabet M. Rothenberg
Göteborg University, Göteborg, Sweden

Dr Albertine J. Schuit
School of Social and Behavioral Sciences, University of Tilburg, Tilburg, Netherlands.

Dr Sabine Schulz
University of Maastricht, Maastricht, Netherlands

Dr Amy Subar
Epidemiology and Genomics, Division of Cancer Control, NIH, Bethesda, MD,

Dr Minna Tanskanen
University of Jyväskilä, Jyväskilä, Finland

Dr Ricardo Uauy
Institute of Nutrition and Food Technology (INTA), University of Chile, Santiago Chile.

Dr Mauro E. Valencia
Department of Nutrition and Metabolism, Nutrition Coordination, Research Center for Food and Development (CIAD), A.C. Hermosillo, Sonora, Mexico.

Dr Giulio Valenti
NUTRIM, Maastricht University, Maastricht, The Netherlands.
Phillips Research, Eindhoven, The Netherlands.

Dr Rita Van den Berg-Emons
Maastricht University, Maastricht, The Netherlands

Dr Wim G. Van Gemert
Maastricht University, Maastricht, The Netherlands

Dr Erica J. Velthuis-te Wierik
TNO Nutrition and Food Research Institute, Zeist, The Netherlands

Dr Wilhelmine W. Verboeket-van de Venne
Maastricht University, Maastricht, The Netherlands

Dr Jeanine Verbunt
NUTRIM, Maastricht University, Maastricht, The Netherlands.

Dr Jonathan C. K. Wells
Population, Policy and Practice Research and Teaching Department, UCL Great Ormond Street Institute of Child Health, London, UK.

Dr Brian M. Wood

Department of Anthropology, University of California Los Angeles, Los Angeles, USA.
Max Planck Institute for Evolutionary Anthropology, Department of Human Behavior, Ecology, and Culture
